# Effectiveness of birth plan counselling based on shared decision making: A cluster randomized controlled trial (APLANT)

**DOI:** 10.1371/journal.pone.0274240

**Published:** 2022-09-12

**Authors:** Encarnación López-Gimeno, Gloria Seguranyes, Mercedes Vicente-Hernández, Lucia Burgos Cubero, Griselda Vázquez Garreta, Gemma Falguera-Puig

**Affiliations:** 1 Midwife, Catalan Health Institute (ICS), Sexual and Reproductive Healthcare Services (ASSIR), Barcelona, Spain; 2 Research Group GRASSIR, Barcelona, Spain; 3 Faculty of Medicine and Health Sciences, Nursing School, University of Barcelona, Barcelona, Spain; 4 Midwife, Catalan Health Institute (ICS), Sexual and Reproductive Healthcare Services (ASSIR), Badalona, Spain; 5 Midwife, Catalan Health Institute (ICS), Sexual and Reproductive Healthcare Services (ASSIR), Mollet del Vallés, Spain; 6 Midwife, Catalan Health Institute (ICS), Sexual and Reproductive Healthcare Services (ASSIR), Barcelona, Spain; 7 Catalan Health Institute (ICS), Sexual and Reproductive Healthcare Services of Metropolitan North Area (ASSIR), Sabadell, Spain; PLOS: Public Library of Science, UNITED KINGDOM

## Abstract

**Background:**

A birth plan (BP) is a written document in which the pregnant woman explains her wishes and expectations about childbirth to the health professionals and aims to facilitate her decision-making. Midwives’ support to women during the development of the BP is essential, but it’s unknown if shared decision making (SDM) is effective in birth plan counselling. We hypothesized that women who receive counselling based on SDM during their pregnancy are more likely to present their BP to the hospital, more satisfied with the childbirth experience, and have better obstetric outcomes than women who receive standard counselling. We also aimed to identify if women who presented BP to the hospital have better obstetric outcomes and more satisfied with the childbirth experience.

**Methods:**

This was a randomised cluster trial involving four Primary Care Units. Midwives provided BP counselling based on SDM to the women in the intervention group (IG) during their pregnancy, along with a leaflet with evidence-based recommendations. Women in the control group (CG) only received the standard birth plan counselling from midwives. The primary outcomes were birth plan presentation to the hospital, obstetrics outcomes and satisfaction with childbirth experience. The Mackey Satisfaction with Childbirth Scale (MCSRS) was used to measure childbirth satisfaction.

**Results:**

A total of 461 (95.5%) pregnant women received BP counselling (IG n = 214 and CG n = 247). Fewer women in the intervention group presented their BP to the hospital compared to those in the control group (57.8% vs 75.1%; p <0.001). Mean satisfaction with childbirth experience was high in the IG as well as the CG: 150.2 (SD:22.6) vs. 153.4 (SD:21.8); p = 0.224). The information received about childbirth during pregnancy was high in both groups (95.1% vs 94.8%; p = 1.0). Fewer women in the IG used analgesia epidural compared to those in the CG (84.7% vs 91.7%; p = 0.034); women who combined non-pharmacological and pharmacological methods for pain relief were more in number in the IG (48.9% vs 29.5%; p = 0.001) and women who began breastfeeding in the delivery room were more in number in the IG (83.9% vs 66.3%; p = 0.001). Women who presented their BP had a greater probability of using combined non-pharmacological and pharmacological methods for pain relief aOR = 2.06 (95% CI: 1.30–4.30) and early skin-to-skin contact aOR = 2.08 (95% CI: 1.07–4.04).

**Conclusion:**

This counselling intervention was not effective to increase the presentation of the BP to the hospital and women’s satisfaction with childbirth; however, it was related to a lower usage of analgesia epidural, a higher combination of pharmacological and non-pharmacological methods for pain relief and the initiation of breastfeeding in the delivery room. Presenting the BP to the hospital increased the likelihood of using pharmacological and non-pharmacological methods for pain relief, and early skin-to-skin contact.

## Introduction

Midwife care is based on the recognition of the right of a woman to decide her own health- related choices; and promotes participation and improvement of shared decision making (SDM) through a collaborative relationship, information, and adequate advice [1]. In addition, SDM, a collaborative relationship between the woman and the midwife, [2, 3] and the continuity of care [4] are factors related to satisfaction with childbirth and some of the attributes which define woman-centred care [4, 5].

One of the objectives of the birth plan (BP) is to facilitate the autonomy and decision making of women [6]. The BP is a written document in which a woman states her wishes and expectations, as well as informs the professionals who accompany her during childbirth about her preferences regarding aspects related to the birth process [7].

Women consider that the BP improves their autonomy and decision making [8], and believe that the support of healthcare professionals, especially midwives, is essential for elaborating and using BPs [9]. The professionals must adopt strategies for focusing dialogue and thereby facilitate collaboration with women in decision making [10, 11]. Afshar et al. [12] proposed that professionals should use the Epstein [13] model to integrate SDM during the counselling of women and discussion regarding the BP. Epstein et al. propose five steps to communicate the evidence for shared decision making with patients, and that includes: understanding the patient’s (and family member’s) experience and expectations, building partnership, providing evidence (including a balanced discussion of uncertainties), presenting recommendations, and finally, checking for understanding and agreement.

SDM is characterised by a collaborative relationship between the patient and the professional for deciding jointly what option adapts better to the patient, considering their values and preferences after having received the scientific evidence-based information available [14].

With the high medicalization of childbirth and the variability in birth assistance in hospitals, the Ministry of Health of Spain defined the Normal Birth Strategy and introduced BPs to improve the healthcare provided to women with the objective of encouraging the participation of pregnant women in decision making and having autonomy in relation to the birth of their baby [15].

Since then, the percentage of women presenting their BP to the hospital, in Spain, is variable and not well-known, ranging from 2.8% to 69% according to some studies [16, 17].

To enable women to express their choices about childbirth, midwives carry out the BP counselling during prenatal care in the third trimester of pregnancy, and in primary health care. The birth is attended in the reference hospital. When women are in labour, they give their BP to the hospital midwives. The professionals providing care in the primary care units and in the hospital are different, and women meet the professionals who will attend them during childbirth on the same day of the birth.

In addition, the Department of Health of Catalonia (Spain) periodically evaluates the opinion of women about childbirth. This department also assesses the degree of women’s satisfaction with the information received from professionals about pregnancy and childbirth. At the last inquiry, 67% of women considered that they received sufficient information related to their pregnancy and childbirth [18].

We hypothesized that women who receive counselling based on SDM during pregnancy are more likely to present their BP to the hospital, more satisfied with the childbirth experience and have better obstetrics outcomes than women who receive standard counselling. We also aimed to identify whether women who present their BP to the hospital have better obstetric outcomes and are more satisfied with the childbirth experience.

The results of this study are part of a larger study which also evaluated if the counselling intervention changes the preferences of the women in their BP.

## Materials and methods

### Design and setting

This was a multicentre, cluster randomised parallel controlled trial including four Primary Care Units of the National Healthcare (NHC) of Catalonia (Spain). The design of the clusters was to mask the intervention to the professionals of the control group and avoid contamination of the information between the midwives and the women participating in the study. ASSIR health centres are placed in different cities and each one has a different referral hospital, thus minimising contact between women as well as between midwives.

The study was approved by the Ethical Committee of Clinical Investigation of the University Institute for Research in Primary Care, Barcelona in December 2016.

The study period lasted from 1 November 2017 to 8 July 2019.

Our clinical trial was registered in Clinical Trial.gov (NCT03744416) in November 2018. This later registration happened because we were not aware at the time whether this was the general practice for this type of intervention studies. The authors confirm that all ongoing and related trials for this intervention are registered.

### Participants

The inclusion criteria were women over the age of 18 years, with low-medium obstetric risk who underwent prenatal and postpartum controls in one of the participating Primary Care Units and underwent childbirth in an NHC reference hospital. All the women provided informed consent to participate in the study. Women with difficulties in understanding the Spanish language were excluded from the study.

#### Sample

Of the seven Primary Care Units which were eligible from a health sector of Catalonia, three were excluded for presenting noncomparable BPs. Randomisation was blinded in the University of Barcelona, with the Primary Care Units being randomised into four groups: 2 CG and 2 IG using the EPIDAT 4.0. programme.

Midwives from the participating Primary Care Units consecutively recruited the women during the prenatal care follow up.

Sample size calculation was based on the variable “presentation of the BP to the hospital” to detect a minimal difference of 20% between two groups, according to a previous descriptive pilot study (n = 211 women) with a prevalence of BP presentation of 48%. A higher prevalence of around 68% was estimated in the intervention group (IG). α risk of 0.05 and β risk of 0.2 were accepted in the bilateral contrast. It was calculated that 133 women were needed in the control group (CG) and 133 in the intervention group (IG). A loss to follow-up of 15% was estimated. The calculation was made using the macros of the SPSS Version 24 and the clusters design was contemplated.

### Intervention

#### Characteristics of the intervention in the intervention group

The intervention consisted of the training of midwives on SDM, midwife counselling to the pregnant women in relation to the elaboration of BPs based on SDM, and an information leaflet to pregnant women on recommendations related to childbirth.

Training of midwives in shared decision making: The research team elaborated a dossier with scientific evidence on different aspects related to childbirth. Afterwards, a 4 hour in-person training session was made for midwives including the following sections: 1. Scientific evidence on care during childbirth, the perception of women and professionals in relation to BPs. 2. Types of healthcare relationships. 3. Key elements for SDM according to the recommendations of Epstein [13]. A. Understanding the patient’s (and family members’) experience and expectations; B. Building partnership; C. Providing evidence, including a balanced discussion of uncertainties; D. Presenting recommendations; and E. Checking for understanding and agreement; and 4. Training in SDM and the presentation of an information leaflet.

Information leaflet on childbirth for pregnant women: A leaflet was elaborated for pregnant women on the scientific evidence-based recommendations of the different options available in the BPs. The reliability and the validity of the content of the information were evaluated by the technique of nominal consensus among experts. An intraclass correlation coefficient of 0.91 and a Cronbach alpha of 0.90 were obtained.

Midwife’s counselling regarding the BP, based on SDM, to the pregnant women: Between 24–28 weeks of gestation (WG), the women were given the first BP for completing at home. In the following prenatal visit, counselling was conducted by midwives regarding the BP, based on SDM; wherein their completed BP was collected and discussed, according to the steps laid out by Epstein on SDM [13], together with the information leaflet.

Following the counselling, the midwife gave the woman a new BP for completing at home. Between 34–40 WG the woman was told to present the BP to the hospital on the day of childbirth.

#### Characteristics of the intervention in the control group

The intervention of the CG consisted of standard counselling by midwives in relation to the BP in prenatal control visits.

The midwives did not receive any specific training on the BP. Furthermore, they were not informed about the training activity that midwives in the intervention group received, nor about the existence of the leaflet.

**Fig 1:**
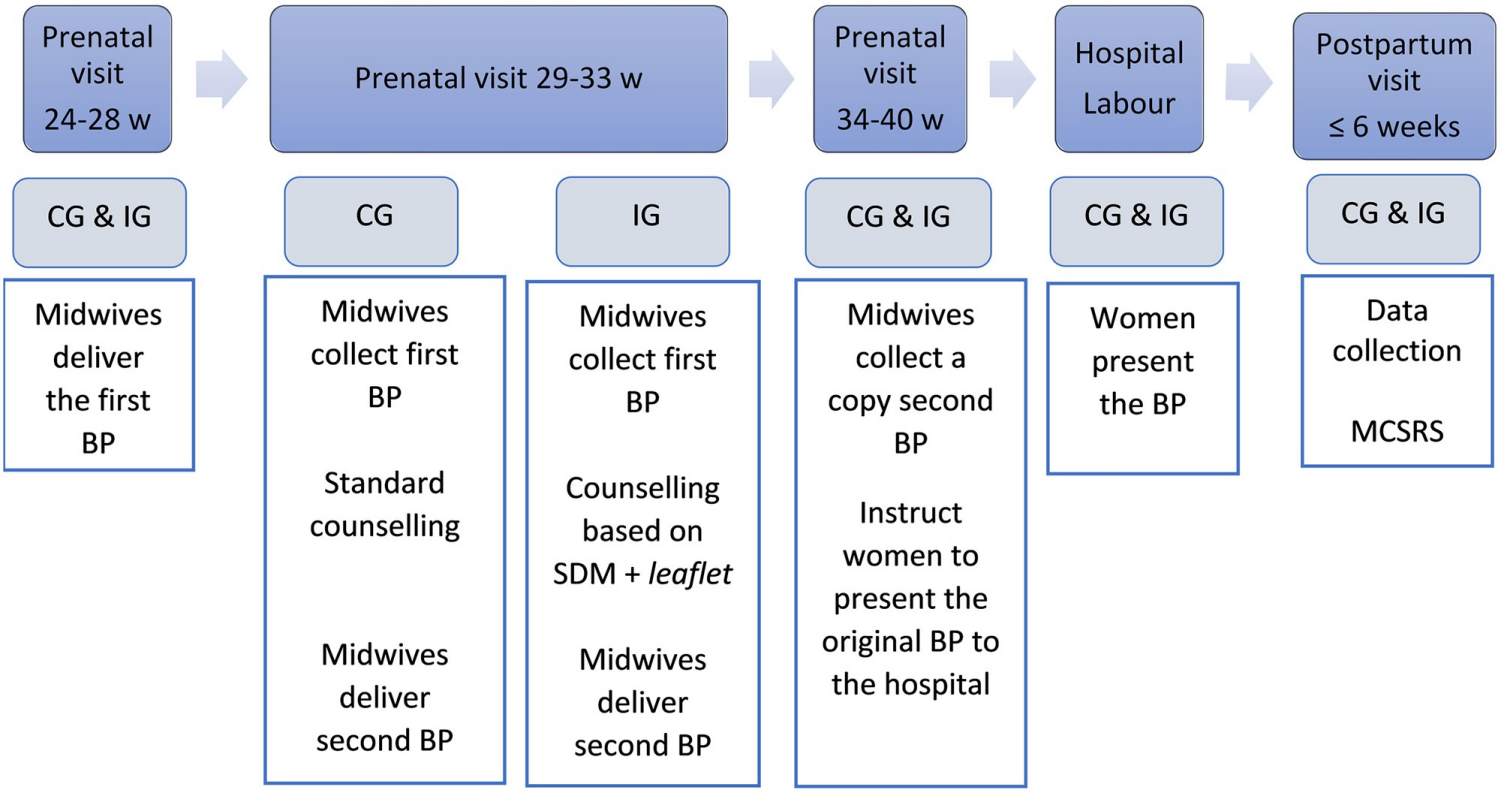
Intervention characteristics and follow up of the pregnant women. W = weeks; CG = control group; IG = intervention group; BP = birth plan; SDM = shared decision making; MCSRS = Mackey Satisfaction with Childbirth Rating Scale.

The pregnant women between 24–28 WG were given the first BP by the midwives for completion at home. In the following prenatal visit, the completed BP was collected, and the women received counselling according to routine midwife practice. Following the counselling, the midwife gave the woman a new BP for completing at home. Later, in the prenatal visit at 34–40 WG, the woman was told to present the BP to the hospital on the day of childbirth. Fig 1 shows intervention characteristics and the women’s follow-up in both groups.

### Outcome measures

The main outcome measures of the study were the presentation of the BP to the reference hospital, global satisfaction with childbirth, satisfaction with the participation in the decision making in relation to the first and second stage of birth, and the obstetric outcomes. Other secondary outcomes were the reasons for not presenting the BP, sufficient information on childbirth during pregnancy, the grade of utility of BP completion in decision making, and the intention of using the BP in subsequent pregnancies. Other secondary outcomes, related to the participants, were the obstetric outcomes (onset of labour, type of birth, episiotomy, early skin to skin contact, initiation of breastfeeding in delivery room, and neonatal and maternal complications), the methods of pain relief [non-pharmacological methods (relaxation and breathing techniques, massage, water use, local heat, birthing ball, others); and pharmacological methods (epidural, intravenous analgesia, nitrous oxide and local and general anaesthesia), and a combination of both methods].

### Data collection

During the recruitment of the study participants, demographic and obstetric data were collected by midwives in a data collection form which included age, country of origin, level of education, employment, partner, previous births and whether the BP had been presented in a previous childbirth.

In the postpartum check, between four and six weeks, midwives of the Primary Care Units collected information about pregnancy (level of obstetric risk, antenatal maternal education, and use of the internet for the BP and childbirth) and childbirth (onset of labour, type of birth, episiotomy, methods of pain relief, early skin-to-skin contact, initiation of breastfeeding in the birthing room, and maternal and neonatal complications). In the same postpartum check, midwives provided a questionnaire to the woman to determine whether she had received sufficient information about childbirth during pregnancy (yes, no), BP (presentation to the hospital, reasons for not presenting the BP, grade of utility of completion of the BP in decision making on a Likert scale [0-none to 5-very useful], if they would use the BP in a subsequent pregnancy (yes, no), and their satisfaction with the childbirth experience. The latter was graded using the Mackey Satisfaction with Childbirth Rating Scale (MCSRS) validated in Spanish for women giving with birth vaginally [19]. This is a self-reporting scale with 35 items. For each of the items a 5-point Likert scale is used and reported as “very dissatisfied” to “very satisfied”, respectively. The overall satisfaction is the sum of all the subscales, with a maximum score of 175 points, and higher scores indicating higher satisfaction. The MCSRS also includes two questions about women’s satisfaction with the participation in the decision making in relation to the first and second stage of birth, using the same 5-points Likert scale.

Women answered the questionnaire and MCSRS in the ASSIR’s waiting room and then handed it to the midwife.

There were no adverse effects during the study.

### Statistical analysis

The data collected were entered to the SPSS Version 24 statistical programme and were only accessible to the investigative team. Descriptive analysis of all the variables was performed. For the childbirth satisfaction outcome, women giving birth via caesarean section were excluded, because the scale was validated Is Spanish for women giving birth vaginally.

Bivariate analysis was performed using the following tests. For comparison of categorical variables, Fisher’s exact test was used; and for the ordinal ones, the Mann-Whitney U test was used. The Student’s t-test was used to compare quantitative continuous variables. A p value <0.05 was considered statistically significant.

Bivariate analysis was performed between the intervention groups and birth plan presentation to the hospital, childbirth satisfaction and obstetrical results. The analyses were performed per protocol basis, because we analysed only women who received intervention and followed-up in the study, according to the treatment group allocated at randomisation.

In addition, we analysed whether the women’s demographic and clinical characteristics were related to the presentation of the BP to the hospital. To evaluate the effect of the intervention counselling with the BP presentation to the hospital a multinomial logistic regression analysis model was performed, which was adjusted to the confounder demographic variables related to the intervention groups (age, country of origin, and education).

Another bivariate analysis was performed to assess whether the presentation of the BP to the hospital was related to the obstetrics outcomes and the grade of satisfaction of women.

Additionally, to determine which obstetric variables (type of birth, epidural, methods of pain relief, early skin-to-skin contact, breastfeeding in delivery room, and maternal and neonatal complications) could have a joint relation with presenting the birth plan, we carried out another multinomial logistic regression model. The model was adjusted by variables that were clinically relevant or those that had a level of significance <0.05 in the bivariate analysis (belonging to the control group or intervention group, age, country of origin, education, previous births).

Additionally, a multinominal logistic regression model was performed modelling the presentation of the birth plan, depending on the variable intervention groups and the variables of age, country of origin, education, previous births, type of birth, epidural, methods of pain relief, early skin-to-skin contact, breastfeeding in delivery room and maternal and neonatal complications, because they were bivariates related to the birth plan presentation, or as adjustment variables for their clinical interest.

Finally, the same model was made, but making an automatic selection of variables (stepwise) to identify which variables had a statistically significant relation with the fact of presenting the birth plan. Adjusted odd ratio (aOR) and the 95% CI were determined.

### Ethical approval and registration

The study was approved by the Ethical Committee of Clinical Investigation of the University Institute for Research in Primary Care (*IDIAP-Instituto Universitario de Investigación en Atención Primaria*) (P16/157) in December 2016. The women who wished to participate received oral and written information, and all the women recruited provided signed informed consent to participate in the study.

The anonymity of the participating women was always maintained, and strict confidentiality of data management was carried out following the prevailing legislation in Spain. The study was registered in ClinicalTrials.gov with the code NCT03744416.

## Results

**Fig 2:**
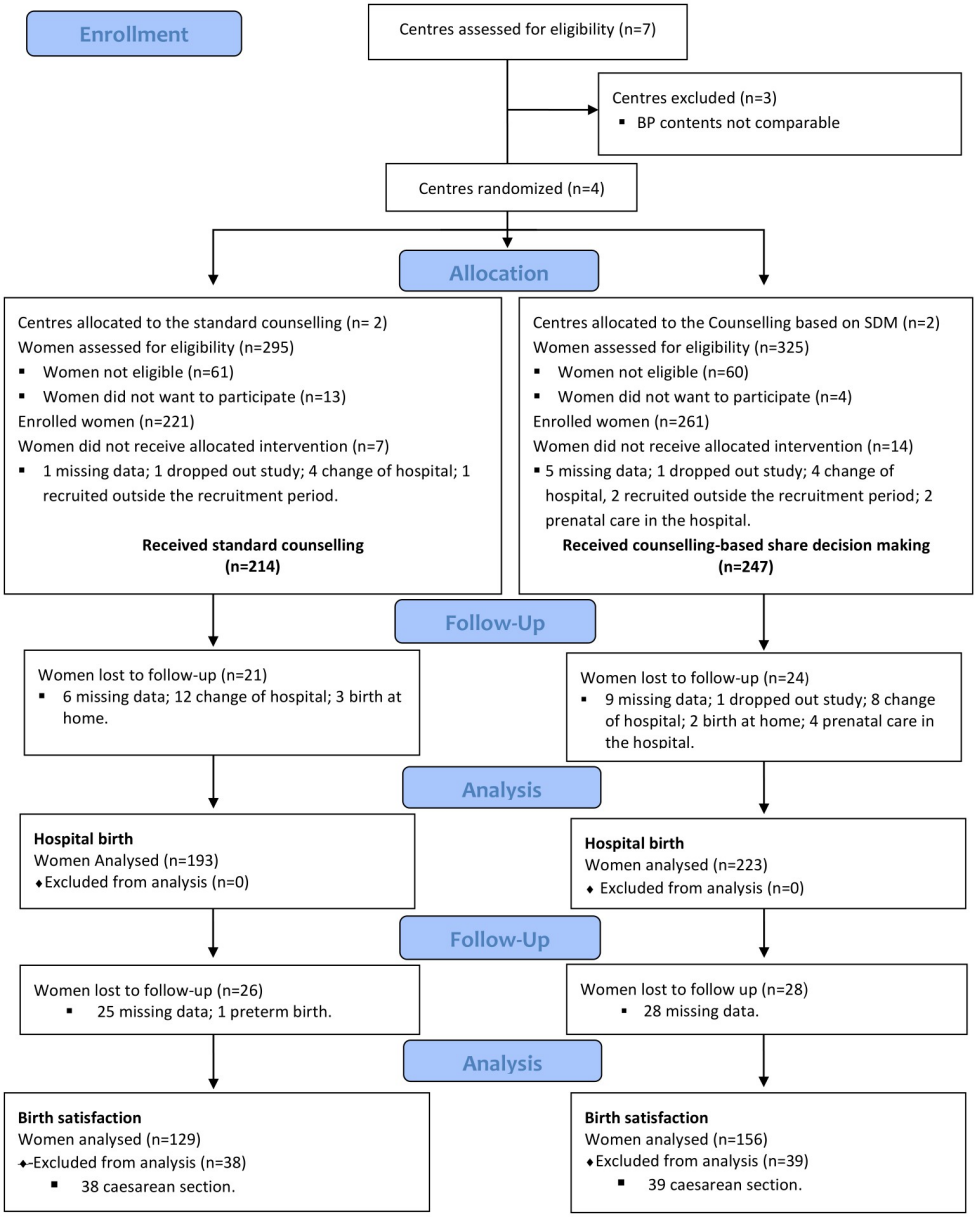
Flowchart of clusters and participants, inclusion, exclusion, and losses to follow-up. SDM: Shared decision making.

A total of 620 pregnant women were invited to participate in the study. Of these, 138 (22.2%) were excluded: 121 (87.7%) for not fulfilling the inclusion criteria and 17 (12.3%) for not wishing to participate. The four participating Care Units recruited a total of 482 (77.7%) pregnant women who initiated the study: 221 (45.9%) in the CG and 261 (54.1%) in the IG. A total of 461 (95.5%) women received BP counselling in both groups: 214 (96.8%) in the CG, and 247 (94.6%) in the IG. Fig 2 shows a flowchart of participant inclusion, exclusion, and losses to follow-up.

The baseline characteristics of the women are shown in Table 1. The occupational situation, having a partner, history of previous births, and elaboration of the BP in a previous pregnancy was similar between the two groups. However, in the IG, the age of the women was higher (mean = 32.4 vs. 31.2; p = 0.028), and there was a higher percentage of women from other countries (32.7% vs. 20.2%; p = 0.005) and with a university education (46.2% vs. 29.5%, p< 0.001).

**Table 1:** Baseline characteristics of the participants.

	Total	Control Group	Intervention Group	*P*
	N = 416	n = 193 (46.4)	n = 223 (53.6)	
	n (%)	n (%)	n (%)	
**Age**, Mean (SD)	31.9 (5.5)	31.2 (5.1)	32.4 (5.7)	0.028^1^
**Country of origin**				
Spain	304 (73.1)	154 (79.8)	150 (67.3)	0.005^2^
Other	112 (26.9)	39 (20.2)	73 (32.7)	
**Education**				
Primary school or less	79 (19)	47 (24.4)	32 (14.3)	<0.001^2^
High school	177 (42.5)	89 (46.1)	88 (39.5)	
University	160 (38.5)	57 (29.5)	103 (46.2)	
**Employment**				
No	99 (23.8)	44 (22.8)	55 (24.7)	0.729^2^
Yes	317 (76.2)	149 (77.2)	168 (75.3)	
**Partner**				
No	22 (5.3)	10 (5.2)	12 (5.4)	1.0^2^
Yes	394 (94.7)	183 (94.8)	211 (94.6)	
**Previous birth(s)**				
No	236 (56.7)	103 (53.4)	133 (59.6)	0.233^2^
Yes	180 (43.3)	90 (46.6)	90 (40.4)	
**Previous birth plan**	N = 180	n = 90	n = 90	
No	100 (55.6)	51 (56.7)	49 (54.4)	0.881^2^
Yes	80 (44.4)	39 (43.3)	41 (45.6)	

Data are expressed as n (%); SD: standard deviation;

^1^ = Student’s t-test;

^2^ = Fisher’s exact test

### Counselling intervention and BP presentation to the hospital, women’s satisfaction with childbirth experience, information received and obstetric outcomes

Information on the presentation of the BP to the hospital was available for 416 women (n = 193; 86.3%) of the CG and (n = 223; 85.4%) IG. A lower percentage of BPs were presented to the hospital among the women in the IG who received counselling based on SDM (n = 129; 57.8%) compared to women in the CG who received standard counselling (n = 145; 75.1%; p < 0.001) (Table 2). Analysis of the presentation of the BP in each of the hospitals showed a lower proportion of the presentations of the BP in hospital III (IG) (46.6%, p < 0.001). When analysing the reasons for not presenting the BP, the main reason was because the professionals had not asked for it (n = 106; 74.7%), with a high percentage in the IG compared to the CG (77.6% vs 68.8%; p = 0.039) (Table 2).

**Table 2:** Counselling intervention and BP presentation to the hospital, women’s satisfaction with childbirth, experience, information received, and secondary outcomes in both study groups.

	Total N = 416	Control Group n = 193 (46.4)		Intervention Group n = 223 (53.6)		*p*
	n (%)	n (%)		n (%)		
**Birth plan presentation to the hospital**						
No	142 (34.1)	48 (24.9)		94 (42.2)		
Yes	274 (65.9)	145 (75.1)		129 (57.8)		<0.001^1^
**Birth plan presentation by hospital**	N = 416	**Hospital I** n = 87 (45)	**Hospital II** n = 106 (55)	**Hospital III** n = 118(52.9)	**Hospital IV** n = 105(47.1)	
No	142(34.1)	26 (29.9)	22 (20.7)	63 (53.4)	31 (29.5)	<0.001^1^
Yes	274 (65.9)	61 (70.1)	84 (79.3)	55 (46.6)	74 (70.5)	
**Reason for not presenting the BP to the hospital**	N = 142	48 (33.8)		94 (66.2)		
Professionals did not ask me for it	106 (74.7)	33 (68.8)		73 (77.6)		0.039^1^
Did not think it was necessary	4 (2.8)	4 (8.3)		0 (0)		
I forgot	10 (7)	4 (8.3)		6 (6.4)		
Other	22 (15.5)	7 (14.6)		15 (16)		
**Childbirth Satisfaction-MCSRS**	N = 285 mean (SD)	n = 129 mean (SD) (CI 95%)		n = 156 mean (SD) (CI 95%)		*P*
**Overall satisfaction**	151.9 (22.3)	153.4 (21.8) (149.6–157.2)		150.2 (22.6) (146.6–153.8)		0.224^2^
**Participation in decision making: first stage**	4.13 (1)	4.22 (4.05–4.38)		4.06 (3.89–4.23)		0.190^2^
**Participation in decision making: second stage**	4.22 (0.9)	4.22 (4.06–4.39)		4.22 (4.07–4.38)		0.996^2^
**Sufficient information on childbirth during pregnancy**	N = 416	n = 193 (46.4)		n = 223 (53.6)		
No	21 (5)	10 (5.2)		11 (4.9)		
Yes	395 (95)	183 (94.8)		212 (95.1)		1.0^1^
**Grade of utility of BP in decision making**	N = 416	n = 193		n = 223		
0	28 (6.7)	14 (7.3)		14 (6.3)		0.421^3^
1	16 (3.8)	9 (4.7)		7 (3.1)		
2	29 (7)	13 (6.7)		16 (7.2)		
3	71 (17.1)	28 (14.5)		43 (19.3)		
4	107 (25.7)	46 (23.8)		61 (27.3)		
5	165 (39.7)	83 (43)		82 (36.8)		
**Would use the BP again in a subsequent pregnancy**	N = 415	n = 192*		n = 223		
No	36 (8.7)	20 (10.4)		16 (7.2)		0.294^1^
Yes	379 (91.3)	172 (89.6)		207 (92.8)		

Data are expressed as n (%); SD = standard deviation;

^1^ = Fisher’s exact test;

^2^ = Student’s t-test;

^3^ = Mann-Whitney U-test;

Mean (95%CI); BP = birth plan; MCSRS = Mackey Satisfaction with Childbirth Rating Scale;

*1 missing value

Concerning the satisfaction with the childbirth experience, information was obtained from 285 (59.1%) MCSRS questionnaires, since women giving nonvaginal birth, birth at home, preterm birth < 36 WG, and incomplete information (Fig 2) were excluded. The mean global satisfaction was high in the control group and similar to the intervention group [153.4 (SD:21.8) vs 150.2 (SD:22.6); p = 0.224)]. Related to the question on the level of participation in decision making during labour and delivery of the MCSRS, the mean was similar in both groups (Table 2).

Sufficient information on childbirth was received during pregnancy according to 95.1% (n = 212) of the women in the IG, and 94.8% (n = 183) in the CG (Table 2).

Also, the BP was considered to be useful or very useful (score equal to 4 or 5) for decision making during childbirth by 64.1% (n = 143) of the women in the IG compared to 66.8% (n = 129) in the CG, with no statistically significant differences between the two groups (Table 2). Most of the women in the IG (n = 207; 92.8%) and in the CG (n = 172; 89.6%) reported that would use the BP in a subsequent pregnancy (Table 2) (S1 Table).

The obstetric results were similar in both groups for onset of labour, type of birth, episiotomy, early skin-to-skin contact and neonatal and maternal complications. Regarding pain relief methods, a lower proportion of women in the intervention group used pharmacological methods than those in the control group (43.9% vs 65.3% respectively; p = 0.001), and a greater proportion of women in the IG combined pharmacological and non-pharmacological methods (48.9% vs. 29.5%, respectively; p = 0.001). Most of the women in both groups used epidural analgesia, although a larger proportion of women in IG did not use this type of analgesia (15.3% vs. 8.3%, respectively; p = 0.034). Moreover, a greater proportion of women in the IG initiated breastfeeding in the delivery room, compared to those in the CG (83.9% vs. 66.3%, respectively; p = 0.001), (Table 3) (S2 Table).

**Table 3:** Counselling intervention and obstetric results in both study groups.

	Total	Control Group	Intervention Group	*P*
	N = 416	n = 193(46.4)	n = 223(53.6)	
	n (%)	n (%)	n (%)	
**Onset of labour**	N = 403	191 (47.4)	212 (52.6)	
Spontaneous	273 (67.7)	125 (65.4)	148 (69.8)	0.393^1^
Induced	130 (32.3)	66 (34.6)	64 (30.2)	
**Type of birth**	N = 416	n = 193 (46.4)	n = 223(53.6)	
Spontaneous vaginal	291(70)	139 (72)	152(68.2)	0.150^1^
Operative vaginal	48(11.5)	16 (8.3)	32 (14.3)	
Caesarean section	77(18.5)	38 (19.7)	39(17.5)	
**Episiotomy**				
No	308 (74)	141 (73)	167 (74.9)	0.736^1^
Yes	108 (26)	52 (27)	56 (25.1)	
**Epidural**				
No	50 (12)	16 (8.3)	34 (15.3)	0.034^1^
Yes	366 (88)	177 (91.7)	189 (84.7)	
**Methods of pain relief**				
Non- pharmacological	26 (6.3)	10 (5.2)	16 (7.2)	0.001^1^
Pharmacological	224 (53.8)	126 (65.3)	98 (43.9)	
Both	166 (39.9)	57 (29.5)	109 (48.9)	
**Early skin-to-skin contact**				
No	43 (10.3)	25 (13)	18 (8.1)	0.109^1^
Yes	373 (89.7)	168 (87)	205 (91.9)	
**Initiation of breastfeeding**				
No	101 (24.3)	65 (33.7)	36 (16.1)	0.001^1^
Yes	315 (75.7)	128 (66.3)	187 (83.9)	
**Neonatal complications**				
No	382 (91.8)	177 (91.7)	205 (91.9)	1.0^1^
Yes	34 (8.2)	16 (8.3)	18 (8.1)	
**Maternal complications**				
No	383 (92.1)	176 (91.2)	207 (92.8)	0.587^1^
Yes	33 (7.9)	17 (8.8)	16 (7.2)	

Data are expressed as n (%);

^1^ = Fisher’s exact test.

### Demographic and clinical characteristics and the relationship with the presentation of the BP in the hospital

No relationship was found between the demographic, and clinical variables and whether the BP was presented in the hospital or not (Table 4).

**Table 4:** Demographic and clinical characteristics related to hospital birth plan presentation.

Birth plan presentation to the hospital	Total	No n (%)	Yes n (%)	*P*
	N = 416	n = 142 (34.1)	n = 274 (65.9)	
**Age**, Mean (SD)	31.9 (5.5)	32.3 (5.4)	31.7 (5.5)	0.312^1^
**Country of origin**				
Spain	304 (73.1)	99 (69.7)	205 (74.8)	0.295^2^
Other	112 (26.9)	43 (30.3)	69 (25.2)	
**Education**				
Primary school or less	79 (19)	24 (16.9)	55 (20.1)	0.577^2^
High school	177 (42.5)	65 (45.8)	112 (40.9)	
University	160 (38.5)	53 (37.3)	107 (39)	
**Previous birth(s)**				
No	236 (56.7)	76 (53.5)	160 (58.4)	0.350^2^
Yes	180 (43.3)	66 (46.5)	114 (41.6)	
**Previous birth plan**	N = 180	n = 66(36.7)	n = 114 (63.3)	
No	100 (55.6)	39 (59.1)	61 (53.5)	0.534^2^
Yes	80 (44.4)	27 (40.9)	53 (46.5)	
**Maternal education**	N = 416	n = 142 (34.1)	n = 274 (65.9)	
No	186 (44.7)	70(49.3)	116(42.3)	0.179^2^
Yes	230 (55.3)	72 (50.7)	158 (57.7)	
**Internet information**				
No	142 (34.1)	47 (33.1)	95 (34.7)	0.827^2^
Yes	274 (65.9)	95 (66.9)	179 (65.3)	
**Obstetric risk**				
Low-medium	297 (71.4)	95 (66.9)	202 (73.7)	0.170^2^
High-very high	119 (28.6)	47 (33.1)	72 (26,3)	

^1^ = Student’s t-test;

^2^ = Fisher’s exact test;

SD: standard deviation

To evaluate the effect of the intervention counselling with the BP presentation to the hospital, a logistic multinomial regression analysis model was performed, adjusted to the confounder demographic variables related to the intervention group. Women in the intervention group had lower likelihood of presenting the BP compared to the control group (OR = 0.45; 95%CI: 0.29–0.70) (Table 5).

**Table 5:** Analysis of demographic factors related to the birth plan presentation and intervention group. Multinomial logistic regression model.

Birth plan presentation to the hospital	*aOR* (95% CI)	*P*
**Intervention Group**		
Control	Ref.	<0.001
Intervention	0.45 (0.29–0.70)	
**Age** Mean (SD)	0.98(0.94–1.02)	0.286
**Country of origin**		
Spain	Ref.	0.495
Other	0.85(0.53–1.36)	
**Education**		
Primary school or less	Ref.	0.351
High school	0.82(0.46–1.46)	
University	1.16(0.62–2.19)	

aOR: adjusted odds ratio; **SD: standard deviation**; 95 CI%: 95% confidence interval

### Presentation of the BP to the hospital and its relation to obstetric outcomes and satisfaction with childbirth experience

Table 6 shows that more women who presented the BP had spontaneous vaginal birth (73.7% vs. 62.7%; p = 0.031) and initiated skin-to-skin contact early (92% vs. 85.2%; p = 0.041) compared to those who did not present the BP. In addition, more of these women combined non-pharmacological and pharmacological methods (44.2% vs. 31.7%; p = 0.048). There were no statistically significant differences between having presented the BP to the hospital and the grade of satisfaction with the childbirth experience.

**Table 6:** Birth plan presentation to the hospital and relation to obstetric results and childbirth satisfaction.

Birth plan presentation to the Hospital	Total	No n (%)	Yes n (%)	*P*	*OR* (95%CI)
	N = 416	n = 142 (34.1)	n = 274 (65.9)		
**Type of birth**					
Spontaneous vaginal	291 (70)	89 (62.7)	202 (73.7)	0.031^1^	Ref.
Operative vaginal	48 (11.5)	17 (12)	31 (11.3)		0.8 (0.42–1.53)
Caesarean section	77 (18.5)	36 (25.3)	41(15)		0.5 (0.3–0.84)
**Methods of pain relief**					
Non-pharmacological	26 (6.3)	10 (7)	16 (5.8)	0.048^1^	Ref
Pharmacological	224 (53.8)	87 (61.3)	137 (50)		1.01 (0.44–2.3)
Both	166 (39.9)	45 (31.7)	121 (44.2)		0.59 (0.25–1.4)
**Epidural**					
No	50 (12)	18 (12.7)	32 (11.7)		Ref.
Yes	366 (88)	124 (87.3)	242 (88.3)	0.753^1^	1.1 (0.59–2.03)
**Early skin-to-skin contact**					
No	43 (10.3)	21 (14.8)	22 (8)	0.041^1^	Ref.
Yes	373 (89.7)	121 (85.2)	252(92)		1.99 (1.05–3.76)
**Breastfeeding in delivery room**					
No	101 (24.3)	35 (24.7)	66 (24.1)	0.904^1^	Ref.
Yes	315 (75.7)	107 (75.3)	208 (75.9)		1.03 (0.64–1.65)
**Neonatal complications**					
No	382 (91.8)	134 (94.4)	248 (90.5)	0.192^1^	Ref.
Yes	34 (8.2)	8 (5.6)	26 (9.5)		1.75 (0.77–3.98)
**Maternal complications**					
No	383 (92.1)	130 (91.5)	253(92.3)	0.849^1^	Ref.
Yes	33 (7.9)	12(8.5)	21 (7.7)		0.9 (0.43–1.88)
**Total childbirth satisfaction**	N = 285	n = 89 (31.2)	n = 196 (68.8)		md (CI 95%)
Mean (SD)	147.7 (21.4)	145.7 (22.1)	148.6 (21.1)	0.295^2^	-2,8 (-8.2 to 2.5)

^1^ = Fisher ‘s exact test;

^2^ = t Student’s test;

md = mean difference; SD: standard deviation; CI 95% = Confidence interval 95%

The results of the multinominal logistic regression, modelling the presentation of the birth plan, depending on the variable intervention groups and the demographic, clinical and obstetric variables are presented in Table 7.

**Table 7:** Multinominal analysis of the birth plan presentation to the hospital and the intervention groups, demographic and obstetrical variables.

Birth plan presentation to the hospital	*OR*	*p*
	95%CI	
**Intervention Group**		
Control	Ref.	< .001
Intervention	0.38 (0.24–0.61)	
**Age Mean (SD)**	0.9 9(0.94–1.03)	0.514
**Country of origin**		
Spain	Ref.	0.298
Other	0.77 (0.47–1.26)	
**Education**		
Primary school or less	Ref.	0.613
High school	0.81(0.44–1.5)	
University	1.03(0.53–1.99)	
**Previous birth(s)**		
No	Ref.	0.351
Yes	0.79(0.49–1.29)	
**Type of birth**		
Spontaneous vaginal	Ref.	0.298
Operative vaginal	0.62(0.33–1.18)	
Caesarean section	0.75(0.37–1.52)	
**Epidural**		
No	Ref.	0.943
Yes	1.04 (0.4–2.68)	
**Methods of pain relief**		
Pharmacological	Ref	0.032
Non-pharmacological	1.08 (0.31–3.81)	
Both	1.9 (1.16–3.11)	
**Early skin-to-skin contact**		
No	Ref.	0.131
Yes	1.89(0.83–4.3)	
**Breastfeeding in delivery room**		
No	Ref.	0.989
Yes	1(0.58–1.73)	
**Maternal complications**		
No	Ref.	0.981
Yes	0.99(0.44–2.25)	
**Neonatal complications**		
No	Ref.	0.137
Yes	1.95 (0.81–4.7)	

aOR: adjusted odds ratio; 95% CI: 95% confidence interval

Finally, the logistic analysis model adjusted for confounders and clinical variables showed that women in IG had a lower likelihood of presenting the BP compared to women in the CG (aOR = 0.37; 95%CI: 0.24 to 0.58). Moreover, the women who presented the BP had double the probability of using non-pharmacological and pharmacological methods for pain relief (aOR = 2.06; 95% CI:1.30 to 4.30) and having early skin-to-skin contact (aOR = 2.08; 95% CI: 1.07 to 4.04) compared to those who did not present the BP (Table 8).

**Table 8:** Multinomial analysis of the birth plan presentation to the hospital with obstetrics results and intervention group. Stepwise logistic regression.

Birth plan presentation to the hospital	*aOR* (95% CI)	*P*
**Intervention Group**		
Control	Ref.	<0.001
Intervention	0.37(0.24–0.58)	
**Methods of pain relief**		
Non-pharmacological	Ref	0.008
Pharmacological	1.13 (0.47–2.69)	
Both	2.06 (1.30–4.30)	
**Early skin-to-skin contact**		
No	Ref.	0.031
Yes	2.08 (1.07–4.04)	

aOR: adjusted odds ratio; 95% CI: 95% confidence interval

## Discussion

In this cluster randomised controlled trial, the intervention of BP counselling based on SDM together with the presentation of a leaflet during pregnancy did not show to be effective for increasing the presentation of the BP to the hospital during childbirth, or raising the grade of satisfaction with the childbirth experience; however, it was related to lower usage of epidural analgesia and higher combination of pharmacological and non-pharmacological methods for pain relief, and early breastfeeding.

The BP was presented to the hospital by 65.9% of the women, being a higher value than that in other studies reporting 34.7% in Netherlands and 48.8% in USA [20, 21]; but being lower than a study conducted in Catalonia (Spain) in 2015 [17]. The percentage of women having received sufficient information about childbirth during pregnancy was higher (around 95%) than the survey carried out by the Catalan Health Department which was 67% in 2016 [18]. This higher percentage can be explained because all women in the present study received some BP counselling, standard or based on SDM. This result is in accordance with the Camacho study [22], which states that midwives are key professionals in advising women.

The degree of usefulness of the BP in decision-making about childbirth and the intention to use the BP was similar in the two groups. The women’s intention of using BP in a subsequent pregnancy was high and similar in the two groups; this is consistent with the results of the Pennell study [23]. Despite this, a third of the women did not present BP to the hospital, with a higher number of women who did not present it among those in the IG; particularly in hospital III where more than half of the women did not present it. Furthermore, the women reported that the main reason for not presenting BP was that the hospital professionals did not request it. The SDM-based counselling was not sufficient to improve the presentation of the BP to the hospital. As stated by Elwyn [24], in addition to SDM techniques and support tools, strategies at different organisational levels should be adopted, such as developing health policies to achieve routine collaboration and deliberation among professionals and patients, which could improve health results. Other factors which may be related to the implementation of the BP could be a possible imbalance of power between the women and the professionals [7], the negative perceptions of some professionals towards BPs [25], and determined hospital policies and protocols [26] which may have an impact on empowerment of the woman [27].

The grade of overall satisfaction with the childbirth experience was high (151.9 of a maximum of 175 points) and similar in both groups, and consistent with the results of the studies by Goodman [28] and Farahat [29]. There are different dimensions of satisfaction with childbirth and the aspects predicting these dimensions [30], such as the personal control, the perception of choice, self-efficacy, and fulfilment of expectations being of note [28, 30–32]. Some studies suggest that SDM and the use of support elements for their implementation are related to greater satisfaction with the decision making [33] in contrast with other studies that do not [34, 35]. It should be taken into account that the professionals who attended the childbirth of the women in the study (Hospital) were different from those who carried out the counselling (Primary Care Units), and this might explain the results obtained.

Regarding obstetric outcomes, SDM counselling influenced the use of pain relief methods and increased the initiation of breastfeeding. Possibly, by receiving this counselling, the participating women had more knowledge about the alternatives to pain relief and the benefits of early breastfeeding and decided to choose these options. These results agree with the statement that SDM and its help elements influence attitudes, knowledge [36, 37] and behaviour [38], but differs from the suggestions of Torigoe and Shorten [37], and Shay et al. [39], who said that these do not influence people’s behaviour.

More of the women who presented the BP combined non-pharmacological methods, although most women used epidural analgesia. This result differs from the Asfhar study [21], in which the use of epidural analgesia was lower among women with BP; but it is similar to another study conducted in Spain [40]. Likewise, presenting the BP was also associated with a higher probability of using combined non-pharmacological and pharmacological methods for pain and early skin-to-skin contact. These results differ from another study [41], but are consistent with the Suarez et al. [16] study in which women with a BP more frequently performed skin-to-skin.

The satisfaction with childbirth was similar between women who provided the BP and those who did not. This finding is similar to the study by Jolles et al. [20], but differs from Asfhar et al., [21] in which women who used the birth plan expressed significant less satisfaction.

### Strengths and limitations

To the best of our knowledge, this is the first study to evaluate the effectiveness of a specific counselling strategy for professionals and pregnant women to approach and discuss BPs. Another strength was the nature of the study. The sample size had adequate power for the principal study variable, and the randomised cluster design of the sample avoided contamination of the information between the midwives and the women.

The IG was made up of a larger number of women from different countries and with an older age and higher level of education, which may have influenced the results; although no relationship was found in the analysis performed. Another factor was that the training intervention of BP counselling by the midwives was only performed among those from primary care and not in midwives from hospitals, which may have had an impact on the presentation of the BP to the hospital. Another possible limitation of the study is that it did not assess satisfaction in the fulfilment of the preferences indicated in the BP.

### Implications in clinical practice and investigation

As a proposal for improvement, we believe that the development and evaluation of a new BP counselling intervention should be approached jointly by midwives in both the hospital and primary care settings to achieve optimum effectiveness. In addition, hospital midwives should routinely determine whether pregnant women have completed a BP in order to discuss it in the childbirth process. Future studies are needed to investigate the effectiveness of SDM interventions in BP counselling with the incorporation of tools to aid in decision making. Qualitative investigation in women and professionals can provide information on the aspects that favour, or limit BP counselling based on SDM.

## Conclusions

An SDM-based counselling intervention during pregnancy was not effective in increasing the presentation of BP to the hospital. It was related to lower usage of analgesia epidural, a higher combination of pharmacological and non-pharmacological methods for pain relief, and initiation of breastfeeding in the delivery room. Satisfaction related to the birth experience of women was high and similar in both groups. In addition, the presentation of BP was associated with the use of pharmacological and non-pharmacological methods for pain relief and early skin-to-skin contact.

## Supporting information

S1 Table Counselling intervention: Main and secondary outcomes by hospitals. (DOCX)

S2 Table Counselling intervention: Obstetric results by hospitals. (DOCX)

S1 Checklist CONSORT 2010 Checklist of information to include when reporting a cluster randomised trial. (DOCX)

S1 File Protocol (Spanish). (PDF)

S2 File Protocol of study. (PDF)
