# Supplementary material for: Effectiveness of birth plan counselling based on shared decision making: A cluster randomized controlled trial (APLANT)
Source: PLoS One. 2022 Sep 12;17(9):e0274240. doi: 10.1371/journal.pone.0274240 (PMC9467369; doi:10.1371/journal.pone.0274240)
Supplement: S1 Table — (DOCX) [file pone.0274240.s001.docx]

**STable 1. Counselling intervention: main and secondary outcomes by hospitals**

|  | **Total**  N=416 | **Control Group**  n=193 (46.4) | | **Intervention Group**  n=223 (53.6) | | ***p*** |
| --- | --- | --- | --- | --- | --- | --- |
|  | n (%) | n (%) | n (%) | n (%) | n (%) |  |
| **Birth plan presentation** | N=416 | **Hospital I**  n=87 (45**)** | **Hospital II**  n=106 (55) | **Hospital III**  n=118 (52.9) | **Hospital IV**  n=105 (47.1) |  |
| No | 142(34.1) | 26 (29.9) | 22 (20.7) | 63 (53.4) | 31 (29.5) | <0.001^1^ |
| Yes | 274 (65.9) | 61 (70.1) | 84 (79.3) | 55 (46.6) | 74 (70.5) |  |
| **Reason for not presenting the BP** | N=142 | n=26 (18.3) | n=22(15.5) | n=63 (44.4) | n=31(21.8) |  |
| Professionals did not ask me for it | 106 (74.7) | 21 (80.8) | 12 (54.5) | 51 (81) | 22 (71) |  |
| Did not think it was necessary | 4 (2.8) | 0 (0) | 4 (18.2) | 0 (0) | 0 (0) |  |
| I forgot | 10 (7) | 2 (7.7) | 2 (9.1) | 4 (6.3) | 2 (6.4) |  |
| Other | 22 (15.5) | 3 (11.5) | 4 (18.2) | 8 (12.7) | 7 (22.6) | 0.029^1^ |
| **Childbirth Satisfaction-MCSRS** | N=285  mean (SD) | Mean  (CI 95%) | mean  (CI 95%) | mean  (CI 95%) | mean  (CI 95%) | *P* |
| Overall satisfaction | 151.9 (22.3) | 148.8  (142.4-155.2) | 157.7  (153.5-161.9) | 148.4  (142.9-153.9) | 152.5  (148.4-156.7) | 0.047^2^ |
| **Participation in decision making: first stage** | 4.13  (1) | 4  (3.7-4.3) | 4.4  (4.2-4.6) | 4  (3.7-4.2) | 4.2  (3.9-4.4) | 0.075^2^ |
| **Participation in decision making: second stage** | 4.22  (0.9) | 4.1  (3.8-4.3) | 4.4  (4.2-4.6) | 4.1  (3.8-4.3) | 4.4  (4.2-4.6) | 0.057^2^ |
| **Sufficient information on childbirth during pregnancy** | N=416 | n=87 (20.9) | n=106 (25.5) | n=118 (28.4) | n=105 (25.2) |  |
| No | 21 (5) | 6 (6.9) | 4 (3.8) | 8 (6.8) | 3 (2.9) | 0.430^1^ |
| Yes | 395 (94.5) | 81 (93.1) | 102 (96.2) | 110 (93.2) | 102 (97.1) |  |
| **Grade of utility of BP in decision making** | N=416 | n=87 (20.9) | n=106 (25.5) | n=118 (28.4) | n=105 (25.2) |  |
| 0 | 28 (6.7) | 7 (8.1) | 7 (6.6) | 9 (7.6) | 5 (4.7) | NA |
| 1 | 16 (3.8) | 4 (4.6) | 5 (4,7) | 5 (4.3) | 2 (1.9) |  |
| 2 | 29 (7) | 9 (10.3) | 4 (3,8) | 9 (7.6) | 7 (6.7) |  |
| 3 | 71 (17.1) | 11 (12.6) | 17 (16) | 30 (25.4) | 13 (12.4) |  |
| 4 | 107 (25.7) | 12 (13.8) | 34 (32.1) | 23 (19.5) | 38 (36.2) |  |
| 5 | 165 (39.7) | 44 (50.6) | 39 (36.8) | 42 (35.6) | 40 (38.1) |  |
| **Would use the BP again in a subsequent pregnancy** | N=415 | n=86 (20.7) | n=106 (25.6) | n=118 (28.4) | n=105 (25.3) |  |
| No | 36 (8.7) | 9 (10.5) | 11 (10.4) | 10 (8.5) | 6 (5.7) | 0.577^1^ |
| Yes | 379 (91.3) | 77 (89.5) | 95 (89.6) | 108 (91.5) | 99 (94.3) |  |

Data are expressed as n (%); 1=Fisher test;2= ANOVA test; 3= Mann-Whitney U test; mean (95%CI); BP= birth plan; MCSRS=Mackey Satisfaction with Childbirth Rating Scale; NA= not applicable
