# Supplementary material for: Effectiveness of birth plan counselling based on shared decision making: A cluster randomized controlled trial (APLANT)
Source: PLoS One. 2022 Sep 12;17(9):e0274240. doi: 10.1371/journal.pone.0274240 (PMC9467369; doi:10.1371/journal.pone.0274240)
Supplement: S2 Table — (DOCX) [file pone.0274240.s002.docx]

**STable 2. Counselling intervention: obstetric results by hospitals**

|  | **Total**  N=416 | **Control Group**  n=193 (46.4) | | **Intervention Group**  n=223 (53.6) | | | ***p*** | |
| --- | --- | --- | --- | --- | --- | --- | --- | --- |
|  | n (%) | n (%) | n (%) | n (%) | n (%) |  | |  |
| **Onset of labours** | N=403 | **Hospital I**  n=85 (21.1**)** | **Hospital II**  n=106 (26.3) | **Hospital III**  n=111 (27.5) | **Hospital IV**  n=101 (25.1) |  | |  |
| Spontaneous | 273 (67.7) | 57 (67.1) | 68 (64.1) | 75 (67.6) | 73 (72.3) | 0.656^1^ | |  |
| Induced | 130 (32.3) | 28 (32.9) | 38 (35.9) | 36 (32.4) | 28 (27.7) |  | |  |
| **Type of birth** | N=416 | n=87 (20.9) | n=106 (25.5) | n=118 (28.4) | n=105 (25.2) |  | |  |
| Spontaneous vaginal | 291 (70) | 68 (78.2) | 71 (67) | 81 (68.6) | 71 (67.6) | 0.330^1^ | |  |
| Operative vaginal | 48 (11.5) | 5 (5.7) | 11 (10.4) | 16 (13.6) | 16 (15.2) |  | |  |
| Caesarean vaginal | 77 (18.5) | 14 (16.1) | 24 (22.6) | 21 (17.8) | 18 (17.2) |  | |  |
| **Episiotomy** |  |  |  |  |  |  | |  |
| No | 308 (74) | 66 (75.9) | 75 (70.8) | 80 (67.8) | 87 (82.9) | 0.060^1^ | |  |
| Yes | 108 (26) | 21 (24.1) | 31 (29.2) | 38 (32.2) | 18 (17.1) |  | |  |
| **Epidural** |  |  |  |  |  |  | |  |
| No | 50 (12) | 3 (3.5) | 13 (12.3) | 16 (13.6) | 18 (17.1) | 0.016^1^ | |  |
| **Yes** | 366 (88) | 84 (96.5) | 93 (87.7) | 102 (86.4) | 87 (82.9) |  | |  |
| **Pain relief methods** |  |  |  |  |  |  | |  |
| Non-pharmacological | 26 (6.3) | 3 (3.5) | 7 (6.6) | 10 (8.5) | 6 (5.7) | <0.001^1^ | |  |
| Pharmacological | 224 (53.8) | 59 (67.8) | 67 (63.2) | 68 (57.6) | 30 (28.6) |  | |  |
| Both | 166 (39.9) | 25 (28.7) | 32 (30.2) | 40 (33.9) | 69 (65.7) |  | |  |
| **Early skin to skin** |  |  |  |  |  |  | |  |
| No | 43 (10.3) | 13 (14.9) | 12 (11.3) | 10 (8.5) | 8 (7.6) | 0.347^1^ | |  |
| Yes | 373 (89.7) | 74 (85.1) | 94 (88.7) | 108 (91.5) | 97 (92.4) |  | |  |
| **Initiation of breastfeeding** |  |  |  |  |  |  | |  |
| No | 101 (24.3) | 27 (31) | 38 (35.9) | 15 (12.7) | 21 (20) | <0.001^1^ | |  |
| Yes | 315 (75.7) | 60 (69) | 68 (64.1) | 103 (87.3) | 84 (80) |  | |  |
| **Neonatal complications** |  |  |  |  |  |  | |  |
| No | 382 (91.8) | 78 (89.7) | 99 (93.4) | 112 (94.9) | 93 (88.6) | 0.285^1^ | |  |
| Yes | 34 (8.2) | 9 (10.3) | 7 (6.6) | 6 (5.1) | 12 (11.4) |  | |  |
| **Maternal complications** |  |  |  |  |  |  | |  |
| No | 383 (92.1) | 84 (96.5) | 92 (86.8) | 113 (95.8) | 94 (89.5) | 0.021^1^ | |  |
| Yes | 33 (7.9) | 3 (3.5) | 14 (13.2) | 5 (4.2) | 11 (10.5) |  | |  |

Data are expressed as n (%); 1=Fisher test.
