# Supplementary material for: Effectiveness of birth plan counselling based on shared decision making: A cluster randomized controlled trial (APLANT)
Source: PLoS One. 2022 Sep 12;17(9):e0274240. doi: 10.1371/journal.pone.0274240 (PMC9467369; doi:10.1371/journal.pone.0274240)
Supplement: S1 File — (PDF) [file pone.0274240.s004.pdf]

Efectividad de una intervención de asesoramiento en el plan de nacimiento en mujeres gestantes

Equipo de investigación

Encarnación López-Gimeno

Gloria Seguranyes

Gemma Falguera Puig

Lucia Burgos Cubero

Mercedes Vicente Hernández

Meritxell Angelet Hidalgo

Judit López Pardo

Griselda Vázquez Garreta

Paula Amezcua La Torre

Eva Vela Martínez

Josep María Manresa Domínguez

Entidades colaboradoras

Instituto Catalán de la Salud (Cataluña, España)

La Fundación Instituto Universitario de Investigación en Atención Primaria de Salud Jordi Gol i Gurina (IDIAPJGol) (Cataluña, España)

Universidad de Barcelona (Barcelona, Cataluña, España)

Índice

	pág.
Resumen	4
Introducción	5
Hipótesis y objetivos	7
Metodología	8
Aspectos éticos	16
Dificultades y limitaciones	16
Aplicabilidad práctica	16
Cronograma	17
Bibliografía	17
Anexos	26

RESUMEN.

Introducción. El plan de nacimiento es la expresión por escrito de las preferencias de la madre sobre el manejo de su parto en aquellos aspectos en los que existen alternativas igual de eficaces y seguras. Su confección puede ayudar a la gestante a tener unas expectativas reales del parto, hacer que se involucre en la toma de decisiones, aumentando así, el control sobre el proceso del nacimiento y tener una experiencia satisfactoria con la experiencia del parto.

Objetivos.

Evaluar la efectividad de una intervención de asesoramiento en el plan de nacimiento, basado en la "toma de decisiones compartida", en mujeres gestantes en relación con la entrega del plan de nacimiento en el hospital y en las preferencias sobre los aspectos relacionados con el parto y la satisfacción con el parto, respecto al asesoramiento habitual.

Evaluar la efectividad de una intervención de asesoramiento en el plan de nacimiento, basado en la "toma de decisiones compartida", en mujeres gestantes en relación con el grado de información recibida en el embarazo sobre el parto, respecto al asesoramiento habitual.

Diseño. Se realizará un estudio experimental, multicéntrico, aleatorio en cluster y paralelo que permitirá conocer la efectividad de una intervención de asesoramiento del plan de nacimiento basado en "la toma de decisiones compartida."

Participantes: Gestantes con control prenatal en los centros de Atención a la Salud Sexual Reproductiva (ASSIR) que deseen participar en el estudio. Tamaño de la muestra: se estudiarán 266 gestantes, 133 en cada uno de los grupos.

Análisis: Se realizará un análisis descriptivo para todas las variables y análisis bivariante mediante pruebas de contraste de hipótesis. Se establecerá un nivel de confianza IC del 95%. Se realizará un análisis multivariante y se calculará el riesgo relativo y el número con IC de 95%.

Palabras clave: plan de nacimiento, asesoramiento, matronas, satisfacción, toma de decisiones.

ANTECEDENTES Y ESTADO ACTUAL DEL TEMA

La experiencia del nacimiento de un hijo es un evento importante en la vida de la mujer. El plan de nacimiento es la expresión por escrito de las preferencias de la madre sobre el manejo de su parto en aquellos aspectos en los que existen alternativas igual de eficaces y seguras [(1)]. Su finalidad principal, es promover la toma de decisiones no influenciadas por las emociones que surgen durante el proceso del parto y proveer de un vehículo de comunicación entre los futuros padres, el proveedor de cuidados y el equipo hospitalario [(2)]. Además, ayuda a la mujer embarazada a comprender sus valores personales, necesidades y preocupaciones en el proceso del nacimiento [(3)]. Su confección puede ayudar a la gestante a tener unas expectativas reales del parto, hacer que se involucre en la toma de decisiones, aumentando así, el control sobre el proceso del nacimiento y tener una experiencia satisfactoria con la experiencia del parto [(4,5)]. Este documento se utiliza para manifestar el conocimiento de sus derechos y deberes como usuaria, de ser responsable de su salud, de su capacidad legal para consentir o no ante determinados procedimientos y también pedir que sean respetadas sus decisiones tal y como recoge la Ley 41/2002 sobre la autonomía del paciente establecido en el art. 4.1[(6)].

El marco competencial de la especialidad de enfermería obstétrico-ginecológica (matronas) incluye la atención del embarazo y parto de bajo y medio riesgo, y en el mismo las matronas deben dar soporte a las gestantes en la confección del plan de nacimiento [(7).] Éste requiere ser abordado, por parte de las matronas, con estrategias que permitan a los futuros padres su cumplimentación [(8)]. Según Epstein et al, la toma de decisiones clínicas deben ser tomadas desde la creación de una relación colaborativa con el paciente y familia usando la mejor evidencia disponible, consistente con los valores, objetivos y capacidades del paciente [(9)].La utilización de herramientas de ayuda en la toma de decisiones de los pacientes respecto a la práctica habitual mejoran la percepción de las probabilidades de los resultados, la congruencia entre la opción escogida y los valores de la persona.[(10)]

La bibliografía disponible sobre la utilidad y la satisfacción de los planes de nacimiento es escasa y no concluyente. Un estudio retrospectivo realizado en Murcia reveló que solo el 2,6 % de las gestantes presentaron el documento del plan de nacimiento en el hospital, además se encontraron diferencias significativas en el

aumento del contacto piel con piel, en el pinzamiento tardío del cordón umbilical, en el aumento de partos eutócicos y en la disminución de la estancia en el hospital en las mujeres que utilizaron el plan de nacimiento (11). En un estudio con metodología cualitativa realizado en Méjico, todas las mujeres mostraron satisfacción con la experiencia de cumplimentar el plan de nacimiento a pesar de que su vivencia no se ajustara completamente a lo especificado en el plan de nacimiento. El mero hecho de escribir sus preferencias fue valorado como una experiencia más personal y gratificante (4). Un ensayo clínico en Taiwán sobre el plan de nacimiento encontró diferencias significativas en relación con el cumplimiento de las expectativas de las mujeres del grupo experimental que mostraron un incremento en el control sobre el proceso del parto, así como una mayor satisfacción en el mismo (5). En cambio, en un estudio experimental realizado en Suecia no encontraron que la realización del plan de nacimiento mejorara la experiencia del parto; aunque si pudiera tener efectos beneficiosos en relación con el miedo al parto y al dolor de las mujeres (12).

La bibliografía también destaca los diferentes puntos de vista que tienen los profesionales sanitarios sobre los planes de nacimiento, en algunos estudios los describen como útiles ya que ponen de relieve las preferencias de la mujer, mejoran la comunicación con las gestantes y abordan las preocupaciones de estas (13). En cambio, otros estudios definen que el término "plan de nacimiento" puede ser engañoso y crear falsas expectativas y ejercen presión sobre las matronas y el equipo multidisciplinar (14).

En España en el año 2007, el Sistema Nacional de Salud elaboró la "Estrategia de Atención al Parto Normal" para dar respuesta a la demanda de las mujeres de participar activamente de su parto (15) y en el mismo año en Cataluña, el Departamento de Salud Catalán publicó el "Protocolo a la asistencia natural del parto normal" (16), y ambos documentos incluyen el "plan de nacimiento".

Dentro de la cartera de servicios de las Unidades de Atención a la Salud Sexual y Reproductiva (ASSIR) en Atención Primaria de Cataluña las matronas realizan el control y seguimiento del embarazo y postparto de las gestantes de bajo y medio riesgo en los centros de salud (17) y la asistencia al parto de las mujeres se realiza en los hospitales de referencia.

En el año 2016 una encuesta realizada en Cataluña sobre la satisfacción de las mujeres en la atención en el proceso de maternidad mostró que el grado de satisfacción global con la atención recibida fue alta, 8,30 sobre 10. Sin embargo, las mujeres refieren que recibieron información suficiente en el embarazo, el parto y el posparto en un 67,2%, 64,2% y 57,6% respectivamente [(18)].

Los profesionales de los ASSIR y los Hospitales de referencia elaboraron conjuntamente el plan de nacimiento con el objetivo de permitir a la mujer la participación en la toma de decisiones, y ser un instrumento de comunicación que favorezca la transmisión de los deseos de la mujer entre atención primaria y hospital. Sin embargo, no se conoce el nivel de implantación del plan de nacimiento; puesto que ni en el ASSIR ni en las áreas obstétricas de los hospitales hay un registro sobre su entrega y utilización. En la revisión bibliográfica efectuada no se ha encontrado suficiente evidencia de la efectividad del asesoramiento que hacen las matronas sobre el plan de nacimiento a las gestantes. Es por ello por lo que se necesitan estudios que verifiquen si un asesoramiento estandarizado en la cumplimentación del plan de nacimiento, basado en la “toma de decisiones compartida”, influye en la entrega de los planes de nacimiento en los hospitales, en las preferencias sobre aspectos del parto, en la satisfacción de la información recibida de las gestantes y en la satisfacción en el parto.

HIPÓTESIS

-Un asesoramiento en el plan de nacimiento de las gestantes, basado en la “toma de decisiones compartida” aumentará la entrega de los planes de nacimiento en el hospital.

-Un asesoramiento en el plan de nacimiento de las gestantes, basado en la “toma de decisiones compartida” modificará: las preferencias sobre los aspectos relacionados con el parto, el grado de satisfacción con la información recibida en la gestación sobre el parto y el grado de satisfacción en el parto, en relación con las mujeres gestantes que reciben el asesoramiento habitual.

OBJETIVOS

Objetivo general

Evaluar la efectividad de una intervención de asesoramiento en el plan de nacimiento, basado en la “toma de decisiones compartida”, en mujeres gestantes en relación con la entrega del plan de nacimiento en el hospital, las preferencias sobre

los aspectos relacionados con el parto y la satisfacción con el parto, respecto al asesoramiento habitual.

Evaluar la efectividad de una intervención de asesoramiento en el plan de nacimiento, basado en la "toma de decisiones compartidas", en mujeres gestantes en relación con la información recibida en el embarazo sobre el parto, respecto al asesoramiento habitual.

Objetivos específicos

1.-Describir las características sociodemográficas y obstétricas de las mujeres gestantes.

2.- Analizar si existen diferencias entre las gestantes de ambos grupos de estudio en las preferencias en cuanto a: acompañante, espacio físico, procedimientos en el parto, métodos de alivio del dolor, segunda etapa del parto, el bebé y la lactancia.

3.-Averiguar la percepción de las gestantes sobre el grado de utilidad de la confección del plan de nacimiento.

4.- Averiguar el grado de satisfacción global de las gestantes en el parto, la satisfacción en la toma de decisiones en la dilatación y en el expulsivo en las gestantes de ambos grupos de estudio.

5.-Determinar los motivos de no entrega de los planes de nacimiento de las gestantes de ambos grupos de estudio.

6. Averiguar si hay diferencias, según las características sociodemográficas y obstétricas de las gestantes, en la entrega de planes de nacimiento en el hospital, en las preferencias de aspectos relacionados con el parto, el grado de la información recibida en el embarazo sobre el parto y en la satisfacción en el parto en ambos grupos de estudio.

METODOLOGÍA

Ámbito de estudio. El estudio se realizará en las unidades del ASSIR de Mollet del Vallés, Granollers, Badalona de la provincia de Barcelona, Área "Metropolitana Nord" y en el ASSIR Eixample Dret, Área "Barcelona Ciudad", en Atención Primaria del Instituto Catalán de Salud. En estas unidades se realizaron un total de 6496 primeras visitas de embarazo en el año 2015.

El estudio tendrá una duración de dos años.

Diseño. Se realizará un ensayo experimental, multicéntrico, aleatorizado por conglomerados y un estudio paralelo que permitirá conocer la efectividad de una intervención de asesoramiento del plan de parto basada en la “toma de decisiones compartida”.

Sujetos.

Población diana. Gestantes con control prenatal en los centros de salud de los ASSIR de Cataluña.

Criterios inclusión**.** Gestantes mayores de edad con control prenatal en los centros de estudio, con parto en los hospitales de referencia, que deseen participar en el estudio y quieran rellenar el plan de nacimiento.

Criterios de exclusión. Analfabetismo, barrera idiomática, drogadicción, alcoholismo, cardiopatía grado 2, 3 y 4, patología materna asociada grave, diabetes tipo I-II, malformación uterina, malformación fetal diagnosticada, amenaza de parto prematuro, gestación gemelar o múltiple, incompetencia cervical, muerte perinatal anterior, retraso de crecimiento intrauterino, placenta previa, isoinmunización, preeclampsia leve-grave, rotura prematura de membranas, infección materna.

Tamaño de la muestra. El cálculo de la muestra se ha realizado en base a la variable principal de estudio "entrega de los planes de nacimiento". Basado en el estudio descriptivo piloto (n=211 gestantes), un 48% de las mujeres que reciben el asesoramiento habitual entregan el plan de nacimiento. Se estima que esta prevalencia puede ser superior en las mujeres que reciban el asesoramiento, alrededor del 68%. En este estudio se plantea un diseño experimental aleatorio, multicéntrico y paralelo en clústers (participan 4 ASSIR). Aplicando un error alfa del 5%, un error beta del 20% y teniendo en cuenta la influencia de los clústers en el análisis, se precisan un total de 133 en el grupo control y 133 en el grupo experimental. Se han estimado un 15% de pérdidas durante el seguimiento. El cálculo se ha realizado mediante las macros del SPSS V22. Se realizará un análisis intermedio para valorar si es necesario recalcular el tamaño muestral para que haya una potencia suficiente para el análisis de los subgrupos.

Técnica de muestreo. En la asignación aleatoria de los ASSIR se tendrán en cuenta las características de los hospitales de referencia, ya que tienen diferentes niveles de asistencia. De forma que dos ASSIR serán asignados al grupo experimental y dos al grupo control. Para esta asignación se utilizará el programa Epidat 4.1. Se ha

optado por este tipo de asignación para evitar la contaminación entre las matronas que recibirán o no formación específica sobre asesoramiento del plan de nacimiento basado en la toma de decisiones compartida.

La técnica de muestreo de las gestantes dentro de cada ASSIR será consecutiva y proporcional al número de primeras visitas (ver tabla 3).

Tabla 3. Muestreo proporcional según el número de primeras visitas de los ASSIR

ASSIR	Primeras visitas	Proporción	Tamaño muestra
Mollet del Vallés	1017	15,1%	41
Badalona	1933	29,6%	79
Granollers	2260	35,7%	94
Dreta Eixample	1286	19,6%	52
Total	6496	100%	266

Intervención

Características de la intervención en el grupo control.

Las matronas en la visita de control gestacional entre 24 a 28 semanas de gestación entregaran el plan de nacimiento para que la gestante lo rellene en su domicilio. En la visita de control gestacional entre las 29 a 33 semanas de gestación las gestantes entregaran el plan de nacimiento a la matrona y las gestantes podrán preguntar sobre los aspectos que deseen. A continuación, las matronas entregarán el segundo plan de nacimiento a las gestantes para que estas lo rellenen en su domicilio y posteriormente deberán entregar una copia a la matrona en la visita de control prenatal entre las 34 a 40 semanas de gestación. La matrona indicará a la gestante que entregue el original en el hospital de referencia.

Características de la intervención en el grupo experimental

Fase I. Intervención formativa de las matronas. Se realizará un taller sobre asesoramiento en el plan de nacimiento de 4 horas, basado en la intervención comunicativa de negociación de opciones en la toma de decisiones compartida de Epstein et al que consiste en: comprensión de la experiencia y expectativas de la gestante, construcción de una relación colaborativa entre la gestante y la profesional, proporcionar información según el nivel de evidencia existente y las recomendaciones actuales y la comprobación de la comprensión y la conformidad[9].

Fase II. Intervención en las gestantes. Las gestantes recibirán por parte de las matronas el plan de nacimiento en la visita de control gestacional entre las 24-28 semanas de gestación que cumplimentarán en su domicilio. Posteriormente en la visita de control gestacional entre las 29-33 semanas de gestación las gestantes recibirán por parte de la matrona la intervención de asesoramiento que consistirá en un asesoramiento estandarizado del plan de nacimiento basado en la toma de decisiones compartida según Epstein et al [(9)] y en la entrega de un folleto informativo [(19)] basado en la evidencia científica existente de los aspectos expresados en el plan de nacimiento. El asesoramiento de la matrona de las gestantes comprenderá las siguientes actividades: explorará las experiencias previas y expectativas de la gestante sobre el parto; establecerá con la gestante una relación colaborativa en la toma de decisiones; explicará de cada aspecto del plan de nacimiento la evidencia existente; informará de las recomendaciones actualizadas sobre los aspectos especificados en el plan de nacimiento y comprobará la comprensión de la información suministrada a la gestante. A continuación, la matrona entregará un segundo plan de nacimiento que la gestante rellenará en su domicilio y que deberá entregar una copia a la matrona en la visita de control prenatal entre las 34-40 semanas de gestación. La matrona indicará que el original lo entregue en el hospital de referencia.

Variables

Variables independientes

Actividad de intervención de asesoramiento basado en la toma de decisiones compartida en el plan de nacimiento

Entrega de folleto informativo: si, no

Variables sociodemográficas y obstétricas

- Nacionalidad: país de origen.
- Edad calculada a partir de la fecha de nacimiento, años y meses.
- Nivel de instrucción: sin estudios, estudios primarios; estudios secundarios; estudios universitarios.
- Empleo: si, no.
- Convive con la pareja: si, no.
- Partos previos: si, no.

- Educación maternal si, no.
- Uso de internet para buscar información sobre el parto: si, no.

Variables de resultados

Variables relacionadas con el plan de nacimiento

- Entrega de plan de nacimiento en el hospital: si, no.
- Causa de no entrega: no lo creí necesario, se me olvidó, los profesionales que me atendieron no me lo pidieron, otros.
- Utilidad de la cumplimentación de plan de nacimiento en la toma de decisiones sobre aspectos del parto. En una escala de intervalo que va del 1 al 5. 0 es nada útil y 5 muy útil.
- Información suficiente recibida en el embarazo sobre el parto: si, no.
- Utilización del plan de nacimiento en el siguiente embarazo: si, no.
- Preferencias comunes expresadas en los planes de nacimiento: si, no
    - Acompañante
    - Espacio físico: espacio único, graduación luminosa.
    - Medidas de confort y apoyo para el parto: escuchar música, pelota de parto.
    - Beber líquidos durante el trabajo de parto.
    - Monitorización fetal: continua, intermitente.
    - Libertad de movimiento.
    - Métodos de alivio del dolor: probar parto sin anestesia, epidural, técnicas de relajación, técnicas de respiración, masajes, otros métodos alternativos no farmacológicos.
- Expulsivo: pujos dirigidos, pujos espontáneos, elegir la posición en el expulsivo, uso del espejo para el parto,
- Bebé: Inicio precoz de piel con piel en sala de partos, pinzamiento tardío del cordón umbilical, iniciar la lactancia materna en sala de partos, no quiero amamantar, consultar mi opinión antes de dar de comer al bebé
- Cambio de preferencias sobre aspectos del parto

Variables obstétricas:

- Inicio de parto: espontaneo, inducido.
- Tipo de parto: eutócico, instrumentado, cesárea.
- Episiotomía: si, no.
- Tipo de analgesia: no farmacológica, farmacológica, ambas.
- Analgesia no farmacológica: relajación, técnicas de respiración, masaje, uso del agua, calor local, TENS, otros: acupuntura, aromaterapia, homeopatía, flores de Bach
- Analgesia farmacológica: peridural.
- Realización de piel con piel en sala de partos: si, no.
- Inicio lactancia materna en sala de partos: si, no.
- Complicaciones perinatales: no, si; ingreso neonato, muerte perinatal, otros.
- Complicaciones maternas: si, no

Variables de satisfacción con la experiencia en el parto

Satisfacción en el parto. Mediante la escala MacKey de satisfacción en el parto (20). En una escala de 1 a 5. 1 muy insatisfecha, 2 poco satisfecha, 3 indiferente, 4 satisfecha, 5 muy satisfecha. Se considerará la puntuación total de la escala y en las subescalas de grado de participación en la toma de decisiones en la dilatación y en el expulsivo.

Recogida de datos e instrumentos

Plan de nacimiento. Es el documento incluido en el Protocolo a la asistencia natural al parto normal del Departamento de Salud de Cataluña [(16)]. Consta de ítems agrupados en apartados que hacen referencia a preferencias sobre acompañante, el espacio físico, procedimientos, métodos del alivio de dolor, aspectos relacionados con la dilatación, el expulsivo, postparto y lactancia. La mujer cumplimenta el plan de nacimiento y puede elegir más de una opción en cada apartado. Los planes de nacimiento son consensuados entre el ASSIR y el hospital de referencia según las características específicas de cada Hospital.

Escala de satisfacción del parto de MacKey. Cuestionario validado al español con coeficiente de interclase de 0,93 para la escala global y con una fiabilidad alfa de Cronbach superior a 0,7 en todas las subescalas [(20)]. Consta de 35 ítems. Cada ítem se valora en escala Likert, 1 como muy insatisfecha, 2 poco satisfecha, 3 indiferente,

4 satisfecha, 5 muy satisfecha. La puntuación global se obtiene sumando los valores asignados a cada ítem. Una mayor puntuación significa una mayor satisfacción.

Hoja de datos sociodemográficos y obstétricos. Confeccionado por el equipo investigador consta de los datos sociodemográficos y variables relacionadas con el parto.

Hoja sobre entrega del plan de parto e información recibida: consta de cuatro preguntas cerradas: recibió información suficiente en el embarazo sobre el parto, entregó el plan de nacimiento, si no lo entregó cual fue la causa, una pregunta de utilidad de la cumplimentación del plan de nacimiento en la toma de decisiones en el parto que se valorará en una escala de 0 a 5, y una pregunta si utilizaría el plan de nacimiento en la siguiente gestación.

Recogida de datos

Las matronas de los ASSIR realizarán la captación de las gestantes del grupo control e intervención en la visita prenatal de las 24-28 semanas de gestación y se les explicará en qué consiste el estudio verbalmente y por escrito. Si desean participar se recogerá el consentimiento informado y a continuación la matrona recogerá los datos sociodemográficos y obstétricos y le entregará el plan de nacimiento para que las gestantes lo cumplimenten en su domicilio.

En la visita prenatal de las 29-33 semanas de gestación las matronas recogerán los planes de nacimiento rellenados y les entregarán un nuevo plan de nacimiento que las mujeres rellenaran en su domicilio y se les indicará que entreguen una copia en la visita prenatal comprendida entre las 34-40 semanas de gestación. La matrona se quedará con una copia e indicará a las gestantes que el original deberán entregarlo en el hospital. En las mujeres del grupo experimental las matronas realizaran la intervención de asesoramiento estandarizado en la confección del plan de nacimiento basado en la toma de decisiones compartida de Epstein.et al y en las gestantes del grupo control las matronas realizaran la práctica habitual de asesoramiento del plan de nacimiento.

En la visita de puerperio comprendida entre el alta hospitalaria y la 6ª semana de postparto las matronas del grupo control e intervención recogerán los datos del parto y entregarán en un sobre cerrado el cuestionario de preguntas sobre la entrega del plan de nacimiento en el hospital, la satisfacción de la información suficiente recibida durante el embarazo sobre el parto, la utilidad de la cumplimentación plan de

nacimiento, la intención de uso en el siguiente embarazo y el cuestionario MacKey sobre la satisfacción del parto.

Prueba piloto. Se realizará una prueba piloto con 10 gestantes en cada ASSIR.

Análisis.

Se realizará un análisis descriptivo de todas las variables. Las cualitativas con frecuencias y porcentajes; las cuantitativas con media, y desviación estándar y si no cumplen una distribución normal se observará la mediana, mínimo y máximo. Se utilizará el paquete de programas estadístico SPSS 22.0. Se realizarán pruebas de homogeneidad entre los grupos para las principales variables sociodemográficas para analizar la comparabilidad de los grupos. Se realizarán las pruebas de contraste de hipótesis, prueba de la Ji al cuadrado y t de Student. Se utilizará un grado de significación estadística del 5%. El nivel de confianza se fijará en el 95%. Se calculará un análisis multivariado, riesgos relativos y número de sujetos con un intervalo de confianza del 95%.

Búsqueda bibliográfica. Las bases de datos consultadas para la búsqueda han sido MEDLINE; Cochrane; EBSCO; CINAHL y CUIDEN entre los años 2000 hasta el 2016.Las palabras clave: planes de nacimiento (*birth plans*), valoración (*assesment*), matronas (*midwife*), satisfacción (*satisfaction*), toma de decisiones (*making decision*).

Aspectos éticos. Se tendrán en cuenta los principios de la Declaración de Helsinki [(21)] y se adoptarán las precauciones necesarias para reducir al mínimo las posibles repercusiones en las gestantes durante la investigación según las recomendaciones del informe Belmont [(22)]. Se solicitará la autorización al Comité de Ética de investigación Clínica de la *Fundació Jordi Gol i Gorina* para los ASSIR del Instituto Catalán de Salud. La hoja informativa y el consentimiento informado es común para los participantes del grupo control y experimental. Durante todo el proceso se garantizará la confidencialidad de los datos según la ley Orgánica15/1999, de protección de datos de carácter confidencial [(23)].

Dificultades y limitaciones. Las mujeres también pueden acceder a la información de plan de nacimiento a través de otras gestantes u otros medios como internet que podría modificar las decisiones respecto al mismo. Para evitar la contaminación entre las matronas en la actividad de asesoramiento se ha

optado por una aleatorización de los ASSIR. Se ha tenido en cuenta en el cálculo de la muestra las posibles pérdidas que podrán deberse a un cambio del riesgo de la gestación posterior a las 29 semanas que limiten seguir el plan de nacimiento cumplimentado y/o requieran un parto en un hospital diferente al de referencia. En situaciones que acontezcan muerte neonatal el grado de satisfacción no será valorable.

Aplicabilidad y utilidad práctica. El asesoramiento en la toma activa de decisiones en el plan de nacimiento puede incorporar mejoras en la toma de decisiones conscientes y mayor autonomía en las gestantes mejorando la información recibida en el embarazo sobre aspectos de su parto. Puede incorporar indicadores de calidad sobre la atención recibida y satisfacción en atención primaria y hospital.

Cronograma

	Setiembre-2016	Octubre-diciembre 2016	Febrero- abril 2017	Marzo- junio 2017	Mayo 2017	Junio-Julio 2017	Septiembre 2017	Octubre 2017	Noviembre 2017 -febrero 2019
Revisión bibliográfica									
Solicitudes y autorizaciones									
Elaboración de un dossier para las matronas del grupo intervención sobre la evidencia científica sobre aspectos que constan en los planes de nacimiento									
Elaboración del díptico provisional									
Reuniones de consenso del díptico									
Diseño definitivo del díptico									
Taller formativo a las matronas del grupo experimental									
Prueba piloto									
Recogida de datos									

Bibliografía.

1. Junta de Andalucía. Plan parto y nacimiento. Sevilla: Junta de Andalucía, Consejería de Salud; 2009.
2. Kaufman T. Evolution of the birth plan. J Perinat Educ. 2007; 16(3):47-52.
3. Bailey JM, Crane P, Nugent CE. Childbirth education and birth plans. Obstet Gynecol Clin North. 2008; 35(3): 497-509.
4. Yam EA, Grossman AA, Goldman LA, Garcia SG. Introducing birth plans in Mexico: an exploratory study in a hospital serving low-income Mexicans. Birth. 2007; 34(1):42-8.
5. Kuo SC, Lin KC, Hsu CH, Yang CC, Chang MY, Tsao CM, Lin LC. Evaluation of the effects of a birth plan on Taiwanese women's childbirth experiences, control and expectations fulfillment: A randomized controlled trial. Inter J Nurs Stud. 2010; 47(7): 806-14.

6. Ley 41/2002 de 14 noviembre básica reguladora de la autonomía del paciente y de derechos y obligaciones en materia de información y documentación clínica. Boletín oficial del estado nº 274, (15-11-2002).

7. Orden SAS/1350/2009, de 6 de mayo, por la que se aprueba y publica el programa formativo de la especialidad de Enfermería Obstétrico Ginecológica. Boletín oficial del estado nº129, (28-05-2009).

8. Anderson CJ, Kilpatrick. Supporting patients' birth plans: theories, strategies & implications for nurses. Nurs Womens Health. 2012; 16(3):210-8.

9. Epstein RM, Alper BS, Quill TE. Communicating evidence for participatory decision making. JAMA. 2004; 291(19): 2359-66.

10. Stacey D, Légaré F, Col NF, Bennett CL, Barry MJ, Eden KB, Holmes-Rovner M, Llewellyn-Thoma. Decision aids for people facing health treatment or screening decision. Cochrane Database of Systematic Reviews 2014, Issue 1.

11. Suarez M ,Armero D,Canteras, M, Martinez E. Use and influence of Deliery and Birth Plans in the humanizing delivery process. Rev. Latino-Am. Enfermagem. 2015; 23(3): 520-6.

12. Lundgren I, Berg M, Lindmarck G. Is the childbirth experience improved by a birth plan? J Midwifery Womens Health. 2003; 48 (5): 322-8

13. Whitford HM[1], Entwistle VA, van Teijlingen E, Aitchison PE, Davidson T, Humphrey T, Tucker JS. Use of a birth plan within woman-held maternity records: a qualitative study with women and staff in northeast Scotland. Birth. 2014;41(3):283-9.

14. Welsh JV, Symon AG. Unique and proforma birth plans: a qualitative exploration of midwives′ experiences. Midwifery. 2014;30(7):885-91

15. Ministerio de Sanidad y Consumo. Estrategia de Atención al Parto Normal en el Sistema Nacional de Salud. Madrid: Ministerio de Sanidad y Consumo; 2007.

16. Generalitat de Catalunya. Protocol per a l' assistència al part normal. Barcelona: Generalitat de Catalunya, Departament de Salut; 2007.

17. Direcció General de Planificació Sanitaria i Avaluació. Cartera de Serveis de les Unitats d'Atenció a la Salut Sexual i Reproductiva de suport a l' Atenció Primària. Barcelona: Generalitat de Catalunya, Departament de Salut; 2007.

18. Divisió d'Atenció al Ciutadà. Qualitat de servei i satisfacció. Estudi de qualitat de servei i satisfacció amb l'embaràs, part i postpart. Barcelona: Generalitat de Catalunya, Servei Català de la Salut; 2016.

19. Dugas M, Shorten A, Dubé E, Wassef M, Bujol E, Chaillet N. Decision aid tools to support women's decision making in pregnancy and birth: a systematic review and meta-analysis. Soc Sci Med. 2012 ;74(12):1968-78

20. Mas R, Barona C, Carregui S, Ibáñez N, Margaix L, Escriba V. Satisfacción de las mujeres con la experiencia del parto: validación de la Mackey Satisfacción Childbirth Rating Scales. Gac Sanit .2012; 26(3):236-42.

21. World Medical Association Declaration of Helsinki. Ethical Principles for Medical Research Involving Human Subjects. Seoul: 59th WMA General Assembly; 2008.

22. The National Commission for protection of Humans subjects of biomedical and Behavioral Research. Ethical principles and guidelines for the protection of human subject research. Belmont; 1978.

23. Ley Orgánica de Protección de Datos de Carácter Personal. Ley 15/1999, de 13 de diciembre. Boletín oficial del estado nº298, (14-12-1999).

24. Simkin P. Birth Plans: After 25 years, women still want to be heard. Birth. 2007; 34(1): 49-51.

25. Lothian J. Birth Plans: The good, the bad, and the future. J Obstet Gynecol Neonatal Nurs. 2006; 35 (2): 295-303.

26. Say R, Thomason R. The importance of patient preferences in treatment decisions challenges for doctors. BMJ. 2003; 327(6):542–5.

27. Woolf S, MD, Chan E C, Harris R, Sheridan S L, Braddock C.H, Kaplan R.M et al. Promoting Informed Choice: Transforming Health Care To Dispense Knowledge for Decision Making. Ann Intern Med. 2005; 143(4):293-300.

28. Hadjigeorgiou E, Kouta C, Papastavrou E, Papadopoulos I, Mårtensson LB. Women's perceptions of their right to choose the place of childbirth: an integrative review. Midwifery. 2012; 28(3):380-90.

29. Roberts CL, Raynes-Greenow CH, Nassar N, Trevena L, McCaffery K. Protocol for a randomised controlled trial of a decision aid for the management of pain in labour and childbirth. BMC Pregnancy Childbirth. 2004; 4(1): 24.

30. Eden KB, Dolan JG, Perrin NA, Kocaoglu D, Anderson N, Case J, Guise JM. Patients were more consistent in randomized trial at prioritizing childbirth preferences using graphic-numeric than verbal formats. J Clin Epidemiol. 2009; 62(4):415-424.

31. Melo-Martin I, Intemann K. Interpreting Evidence: Why values can matter as much as Science. Perspect Biol Med. 2012; 55(1):59-70.

32. Chin G, Warren N, Kornman L, Cameron P. Patients' perceptions of safety and quality

of maternity clinical handover. BMC Pregnancy Childbirth. 2011; 11:58.

33. PennellA ,Salo-Coombs V, Herring A, Spielman F, Fecho K. Anesthesia and analgesia-related preferences and outcomes of women who have birth plans. J Midwifery Womens Health. 2011;56

34. Grant R, Sueda A, Kaneshiro B. Expert opinion vs. patient perception of obstetrical outcomes in laboring women with birth plans. J Reprod Med. 2010; 55(1-2):31-5.

35. Carlton T, Callister L, Christiaens G, Walker D. Labor and delivery nurses' perceptions of caring for childbearing women in nurse-managed birthing units. MCN Am J Matern Child Nurs.2009; 34(1):50-6.

36. Demontis R, Pisu S, Pintor M, D' aloja E. Cesarean section without clinical indication versus vaginal delivery as a paradigmatic model in the discourse of medical setting decisions. J Matern Fetal Neonatal Med. 2011; 24(12):1470-5.

37. Romano AM, Lothian JA. Promoting, Protecting, and Supporting Normal Birth: A Look at the Evidence. J Obstet Gynecol Neonatal Nurs. 2008; 37(1):94-104.

38. National Institut for Health and Clinical Excellence Antenatal Care. Clinical guideline. Routine care for the healthy pregnant woman. London;

39. Raynes-Greenow CH, Roberts CL, McCaffery K, Clarke J. Knowledge and decision-making for labour analgesia of Australian primiparous women. Midwifery. 2007; 23(2):139-45.

40. Declercq E, Sakala C, Corry MP, Applebaum S, Herrich A. Listening to mothers III: Pregnancy and childbirth. Report. New York: Childbirth Connection;2013

[acceso 25 de mayo de 2013]. Disponible en: http://www.maternitywise.org.

41. Deering SH, Zaret J, McGaha K, Satin AJ. Patients presenting with birth plans in a military tertiary care hospital: a descriptive study of plans and outcomes.Mil Med. 2006; 171(8):778-80.

42. Melender HL. What constitutes a good childbirth? A qualitative study of pregnant finnish women. J Midwifery Womens Health.2006; 51:331-9.

43. Philepsen N, Haynes D. The similarities between birth plans and living wills. J Perinat Educ.2005; 14(4):46–8.

44. Chalmers B, Porter R. Assessing Effective Care in Normal Labor: The Bologna Score. Birth.2011; 28:79–83.

45. Cleveland G. Shields, PhD, Franks , Fiscella K MD, Meldrum S, Epstein RM. Rochester Participatory Decision-Making Scale (RPAD): Reliability and Validity. Ann Fam Med. 2005; 3(5): 436–42.

46. Henry A, Nand SL. Women's antenatal knowledge and plans regarding intrapartum pain management at the Royal Hospital for Women. Aust N Z J Obstet Gynaecol. 2004; 44(4):314-7.

47. Shorten A, Chamberlain M, Shorten B, Kariminia A. Making choices for childbirth: development and testing of a decision-aid for women who have experienced previous caesarean. Patient Educ Couns. 2004; 52(3):307-13.

48. Madi BC, Crow R. A qualitative study of information about available options for childbirth venue and pregnant women's preference for a place of delivery. Midwifery. 2003; 19(4):328-36

49. Berg M, Lundgren I, Lindmark G. Childbirth Experience in Women at High Risk: Is It Improved by Use of a Birth Plan? J Perinat Educ. 2003; 12(2):1-15

50. Brooks H, Sullivan W. The importance of patient autonomy at birth. Int J Obstet Anesth. 2002; 11(3):196-203.

51. Hodnett ED. Pain and women's satisfaction with experience of childbirth: a systematic review. Am J Obstet gynecol.2002;186:160-72

52. Lothian JA. Why Natural Childbirth? J Perinat Educ. 2000; 9 (4): 44-6.

53. Deering SH, Zaret J, McGaha K, Satin AJ. Patients presenting with birth plans: a case-control study of delivery outcomes. J Reprod Med. 2007 ;52(10):884-7.

54. Aragon M, Chhoa E, Dayan R, Kluftinger A, Lohn Z, Buhler K. Perspectives of expectant women and health care providers on birth plans. J Obstet Gynaecol Can. 2013 ;35(11):979-85.

55. Hadar E, Raban O, Gal B, Yogev Y, Melamed N.Obstetrical outcome in women with self-prepared birth plan. J Matern Fetal Neonatal Med. 2012;25(10):2055-7.

56. Whitford HM, Entwistle VA, van Teijlingen E, Aitchison PE, Davidson T, Humphrey T, Tucker JS. Use of a birth plan within woman-held maternity records: a qualitative study with women and staff in northeast Scotland. Birth. 2014;41(3):283-9.

57. Brauer S.Moral implications of obstetric technologies for pregnancy and motherhood. Med Health Care Philos. 2016 Mar;19(1):45-54

58. Mei JY, Afshar Y, Gregory KD, Kilpatrick SJ, Esakoff TF. Birth Plans: What Matters for Birth Experience Satisfaction. Birth. 2016 ;43(2):144-50.

59. Cook K. The impact of choice and conrol on women's childbirth experiences.J Perinat

Educ.,21(3),158-168

60. Beatriz Elena Delgado-García, M Isabel Orts-Cortés, Alberto Poveda-Bernabeu, Pablo Caballero-Pérez, Ensayo clínico controlado y aleatorizado para determinar los efectos del uso de pelotas de parto durante el trabajo de parto, Enfermería Clínica, Volume 22, Issue 1, January–February 2012, Pages 35-40, ISSN 1130-8621, http://dx.doi.org/10.1016/j.enfcli.2011.07.001.

61. Grupo de trabajo de la Guía de Práctica Clínica sobre la atención al parto normal. Guía de Práctica Clínica sobre la atención al parto normal. Plan de Calidad para el Sistema Nacional de Salud del Ministerio de Sanidad y Política Social. Agencia de Evaluación de Tecnologías Sanitarias del País Vasco (OSTEBA). Agencia de Evaluación de Tecnologías Sanitarias de Galicia (Avalia-t). 2010. Guías de Práctica Clínica en el SNS: OSTEBA Nº 2009/01.

62. Plan de parto y nacimiento. Agencia de Calidad del Sistema Nacional de Salud. 2012. [Acceso 1-07-2016](Disponible en: www.msssi.gob.es/organizacion/sns/planCalidadSNS/pdf/.../planPartoNacimiento.pdf

63. Guia NICE. Intrapartum care: care of healthy women and their babies during childbirth. [Acceso 1-07-2016](Disponible en: https://www.nice.org.uk/news/article/midwife-led-units-safest-for-straightforward-births

64. Lemos A, Amorim MMR, Dornelas de Andrade A, de Souza AI, Cabral Filho JE, Correia JB. Pushing/bearing down methods for the second stage of labour. *Cochrane Database of Systematic Reviews* 2015, Issue 10. Art. No.: CD009124. DOI:10.1002/14651858.CD009124.pub2

65. Romero ME, Carrizosa A, Francisco J. Las posturas de la mujer de parto en fase de expulsivo: revisión de la evidencia científica y recomendaciones. Med. Naturista. 2014; 8 (1): 23-30.

66. Becerra-Maya Emilio José, Lapuente-Jambrina Gloria, Alonso-Ortega Verónica María. Uso del espejo en el periodo expulsivo del parto: evaluación mediante diferencial semántico. Index Enferm [Internet]. 2011 Jun [citado 2016 Ago 09] ; 20(1-2): 46-50. Disponible en: http://scielo.isciii.es/scielo.php?script=sci_arttext&pid=S1132-12962011001100110&lng=es

67. Ballesteros-Meseguer C, Carrillo-García C, Meseguer-de-Pedro M, Canteras-Jordana M, Martínez-Roche ME. Episiotomy and its relationship to various clinical variables that influence its performance. Rev. Latino-Am. Enfermagem. 2016;24:e2793. [Access03/07/2016]; Available in: http://www.scielo.br/pdf/rlae/v24/es_0104-1169-rlae-24-02686.pdf . DOI: http://dx.doi. org/10.1590/1518-8345.0334.2686.

68. Carroli G, Mignini L. Episiotomía para el parto vaginal. Cochrane Database of Systematic Reviews 2012 Issue 11. Art. No.: CD000081. DOI: 10.1002/14651858.CD000081

69. Devane D, Lalor JG, Daly S, McGuire W, Smith V. Cardiotocography versus intermittent auscultation of fetal heart on admission to labour ward for assessment of fetal wellbeing. Cochrane Database Syst Rev. 2012 Feb 15; (5): CD005122.

70. Alfirevic Z, Devane D, Gyte GM. Continuous cardiotocography as a form of electronic fetal monitoring (EFM) for fetal assessment during labour. Cochrane Database Syst Rev. 2013 May 31; (5): CD006066.

71. Cahill AG, Spain J. Intrapartum fetal monitoring. Clinical Obstetrics and Gynecology. 2015 Jun; 58 (2): 263-8.

72. NICE guidelines. Intrapartum care pathway. Last updated: 09 December 2015

73. Michikata K, Sameshina H, Urabe H, Tokunaga S, Kodama Y, Ikenoue T. The regional centralization of Electronic Fetal Heart Rate Monitoring and its impact on Neonatal Acidemia and the Cesarean Birth Rate. J Pregnancy. 2016; 2016: 3658527.

74. Boatin AA, Wylie BJ, Goldfarb I, Azebedo R, Pittel E, Ng C, Haberer JE. Wireless vital sign monitoring in pregnant women: a functionality and acceptability study. Telemed J E Health. 2016 Jul; 22 (7): 564-71.

75. Iniciativa parto normal. Documento de consenso. 2007. FAME

76. Walter V, Nelly LF. Anales de la Facultad de Medicina. 2008; 69 (2): 127-9. Smyth RMD, Markham C, Dowswell T. Amniotomy for shortening spontaneous labour (Review). Cochrane Database of Systematic Reviews 2013, Isuue 6. Art. No.: CD006167.

77. Wei S, Wo BL, Qi HP, Xu H, Luo ZC, Roy C, Fraser WD. Early amniotomy and early oxytocin for prevention of, or therapy for, delay in first stage spontaneous labour compared with routine care (Review). Cochrane Database of Systematic Reviews 2013, Isuue 8. Art. No.: CD006794. Ghafarzadeh M, Moeininasab S, Namdari M. Effect of early amniotomy on dystocia risk and cesarean delivery in nulliparous women: a randomized clinical trial. Arch Gynecol Obstet. 2015 Aug; 292 (2): 321-5.

78. Reche DM, Guedes Ch, Galindo A. Factores que influyen en la distocia de un parto de inicio espontáneo. [monografía en Internet] *. Almería: Paraninfo Digital; 2015 [acceso 16 de Julio de 2016]. Disponible en: http://www.index-f.com/para/n22/129.php.

79. Harrison MJ, Kushner KE, Benzies K, Rempel G, Kimak C. Women's satisfaction with their involvement in health care decisions during a high-risk pregnancy. Birth. 2003; 30 (2): 109-15.

80. Biurrun-Garrido A, Goberna-Tricas J. La humanización del trabajo de parto: necesidad de definir el concepto (Revisión). Matronas Prof. 2013; 12 (2): 62-66.

81. Reveiz L, Gaitán HG, Cuervo LG.Enemas during labour. Cochrane Database Syst Rev. 2013 Jul 22;(7):CD000330. doi: 10.1002/14651858.CD000330.

82. Kelly AJ, Kavanagh J, Thomas J. Castor oil, bath and/or enema for cervical priming and induction of labour. Cochrane Database Syst Rev. 2013 Jul 24; (7):CD003099. doi: 10.1002/14651858.CD003099.

83. Kovavisarach E, Sringamvong W.Enema versus no-enema in pregnant women on admission in labor: a randomized controlled trial. J Med Assoc Thai. 2005 Dec; 88(12):1763-7.

84. Tzeng YL, Shih YJ, Teng YK, Chiu CY, Huang MY. Enema prior to labor: a controversial routine in Taiwan.J Nurs Res. 2005 Dec;13 (4):263-70.

85. Barbosa da Silva, Flora Maria et al. Prácticas para estimular el parto normal. *Index Enferm*, Set 2011, vol.20, no.3, p.169-173. ISSN 1132-1296

86. Kovavisarach E, Jirasettasiri P. Randomised controlled trial of perineal shaving versus hair cutting in parturients on admission in labor. J Med Assoc Thai. 2005 Sep;88 (9):1167-71.

87. Basevi V, Lavender T.Routine perineal shaving on admission in labour. Cochrane Database Syst Rev. 2014 Nov 14;(11):CD001236. doi: 10.1002/14651858.CD001236.pub2.

88. Gupta JK, Hofmeyr GJ. Posición de la mujer durante el período expulsivo del trabajo de parto (Revisión Cochrane traducida). En: La Biblioteca Cochrane Plus. 2008; 4. Oxford: Update Software Ltd.

89. Roberts CL, Algert CS, Olive E. Impact of first-stage ambulation on mode of delibery among women with epidural analgesia. Aust N Z J Obstet Gynaecol 2004;446:489-94.

90. Souza JP, Miquelutti MA, Cecatti JG, Makuch MY. Maternal position during the first stage of labor: A systematic review. Reproductive Health 2006; 310.

91. Lawrence A, Lewis L, Hofmeyr GJ, Dowswell T, Styles C. Maternal positions and mobility during first stage labour. Cochrane Database Syst Rev 2009

92. Ben Regaya L, Fatnassi R, Khlifi A, Fékih M, Kebaili S, Soltan K, Khairi H, Hidar S Role of deambulation during **labour**: A prospective randomized study. J Gynecol Obstet Biol Reprod (Paris). 2010 Dec;39(8):656-62. doi: 10.1016/j.jgyn.2010.06.007

93. Souza JP, Miquelutti MA, Cecatti JG, Makuch MY Maternal position during the first stage of labor: a systematic review.Reprod Health. 2006 Nov 30; 3:10.

94. O ‘Sullivan G, Liu B, Hart D, Seed P, Shennan A. Effect of food intake during labour on obstetrics outcome: randomised controlled trial. BMJ 2009;338-b784.

95. Singata M,Tranmer J, Gyte GM. Restricting oral fluid and food intake during labour. Cochrane Database Syst Rev 2010;(1):CD003930.

96. Scheepers HC, De Jong PA, Essed GG, et.al. Carbohydrate solution intake during labour just before the start of the second stage: a double-blind study on metabolic effects and clinical outcome. BJOG 2004; 111(12):1382-7.

97. Smith RB, Toledano MB, Wright J, Raynor P, Nieuwenhuijsen MJ.Tap water use amongst pregnant women in a multi-ethnic cohort.Environ Health. 2009 Dec 21;8 Suppl 1:S7. doi: 10.1186/1476-069X-8-S1-S7.

98. Saxton A, Fahy K. Effects o skin-to.skin contact and brestfeeding at birth on the incidence of PPH: A physiologically bases theory.Volume 27, Issue 4, Desember 2014, Pages 250-253.

99. Redshaw M, BA, PhD, Hennegan J, Kruske S.Holding the baby: Early mother-infant contact after childbirth and outcomes. Midwifery 30 (2014) 177-187. doi: 10.1016/j.midw.2014.02.003. Epub 2014 Feb 22

100. Phillips R Sacred Hour: Uninterrupted Skin-to-Skin Contact Immediately After Birth Newborn and Infant Nursing Reviews 13 (2013) 67-72

101. Moore ER, Anderson Gc, Bergman N, Dowxwell T. Early skin-to-skin contact for mother s and their healthy nexborn infants (review)Cochrane Database of Systematic Reviews 2012, Issue 5. Art. Nº : CD 003519. DOI: 10.1002/14651858. CD003519. Pub3

102. Dois A C, Luchhchini C, Villarroel L, Uribe C. Efecto del contacto piel con piel sobre la presencia de síntomas depresivos post parto en mujeres de bajo riesgo obstétrico. Rev Chil Pediatr 213; 84 (3) : 285-292

103. Lucchini C, Marquez F , Uribe C.Efectos del contacto piel con piel del recién nacido con su madre.Index Enferm [Internet]. 2012 Dic [citado 2016 Sep 26] ; 21(4): 209-213. Disponible en: http://scielo.isciii.es/scielo.php?script=sci_arttext&pid=S1132-12962012003300007&lng=es.

Anexos

Anexo 1. Protocol study flow chart

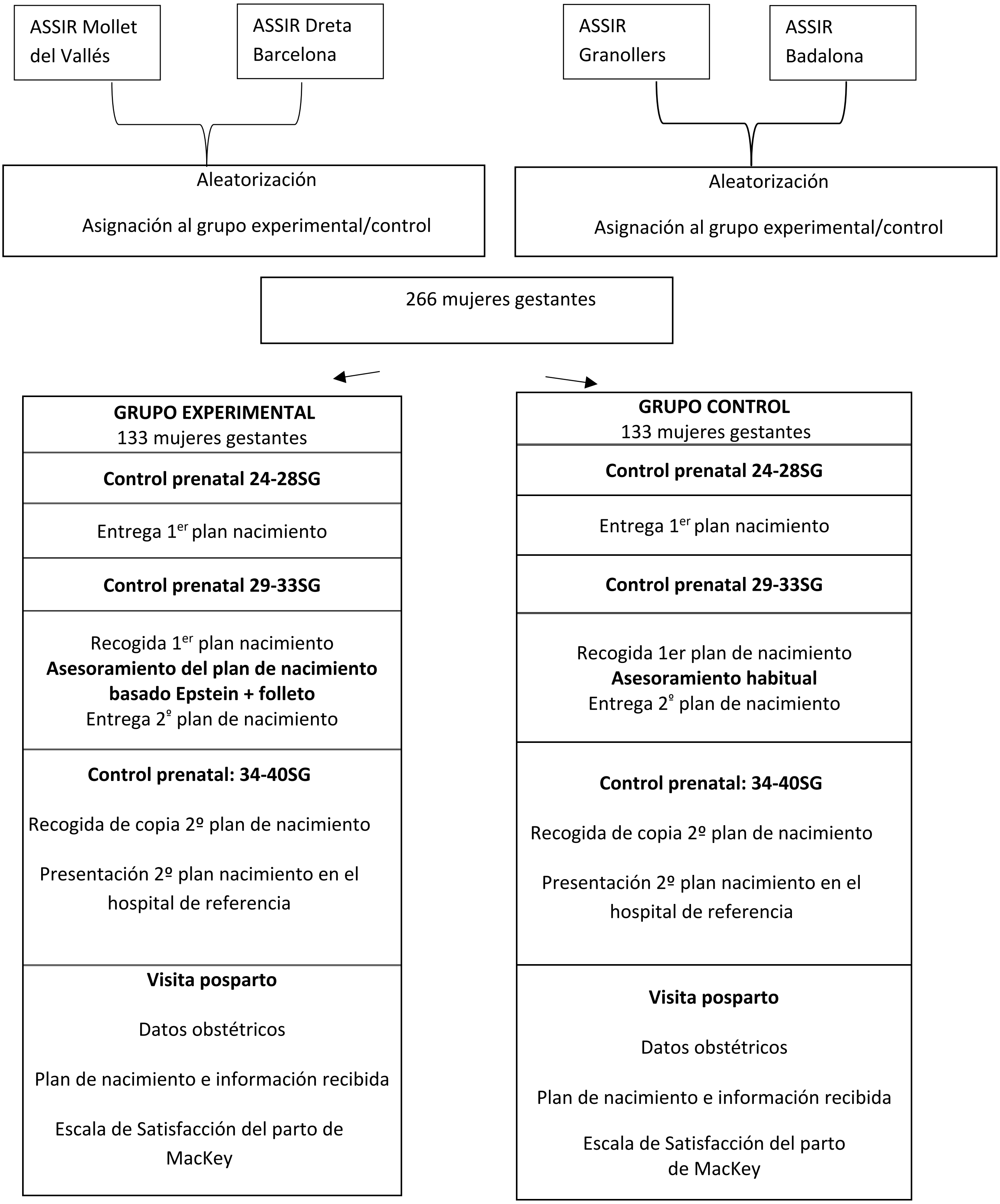

Anexo 2. Hoja informativa para la participante en el estudio "Efectividad de una intervención de asesoramiento del plan de nacimiento en mujeres gestantes"

Este documento tiene por objeto ofrecerle información sobre un estudio de investigación en el que se le invita a participar. Se realiza en diferentes centros de *Atención a la Salud Sexual y Reproductiva* (ASSIR) de la Región y fue aprobado por el Comité Ético en Investigación Clínica del Institut de Investigación en Atenció Primària (IDIAP) Jordi Gol. Si decide participar en el mismo, debe recibir información personalizada por la matrona, leer antes este documento y hacer todas las preguntas que necesite para comprender los detalles sobre el mismo. Si así lo desea, puede llevar el documento, consultarlo con otras personas, y tomarse el tiempo necesario para decidir si participar o no. La participación en este estudio es completamente voluntaria. Puede decidir no participar o, si acepta hacerlo, cambiar de opinión retirando el consentimiento en cualquier momento sin obligación de dar explicaciones. Le aseguramos que esta decisión no afectará a la relación con su matrona ni a la asistencia sanitaria a la que tiene derecho.

¿Cuál es el propósito del estudio? Este estudio el propósito de evaluar la efectividad de una intervención de asesoramiento del plan de nacimiento en mujeres gestantes. Se le invita a participar para poder evaluar la efectividad de una intervención de asesoramiento en el plan de nacimiento en mujeres gestantes. La colaboración y la participación en dicho estudio tienen como finalidad la mejora de la atención recibida de los servicios sanitarios.

¿Por qué me ofrecen participar a mí? La selección de las personas invitadas a participar depende de unos criterios que están descritos en el protocolo de la investigación. Estos criterios sirven para seleccionar a la población en la que se responderá el interrogante de la investigación. Vd. es invitada a participar porque cumple esos criterios. Se espera que participen un total de 266 mujeres.

¿En qué consiste mi participación? Su participación consistirá en rellenar los planes de nacimiento durante la gestación y unas encuestas anónimas que le serán entregadas por la matrona que realice la visita de puerperio sobre aspectos relacionados sobre su parto y el plan de nacimiento. En ningún caso disminuirá el control protocolizado de su atención en el embarazo y en el puerperio. En caso de que se necesitaran nuevos datos podremos ponernos en contacto con usted. La duración prevista de su participación en el estudio será aproximadamente 8 meses.

¿Qué riesgos o inconvenientes tiene? Dedicar su tiempo en la contestación de las encuestas

¿Obtendré algún beneficio por participar? No se espera que Vd. obtenga beneficio directo por participar en el estudio. Se desconoce el uso de los planes de nacimiento, por eso se quiere investigar. El único beneficio buscado es conocer la situación del uso de los planes de nacimiento con el fin de realizar en un futuro posibles mejoras en la asistencia sanitaria.

¿Recibiré la información que se obtenga del estudio? Si Vd. lo desea, se le facilitará un resumen de los resultados del estudio. También podrá disponer de los cuestionarios cumplimentados, si así lo solicita.

¿Se publicarán los resultados de este estudio? Los resultados de este estudio serán publicados en revistas científicas para su difusión, pero no se transmitirá ningún dato que pueda llevar a la identificación de los participantes.

¿Cómo se protegerá la confidencialidad de mis datos? El tratamiento, comunicación y cesión de sus datos se hará conforme a lo dispuesto por la Ley Orgánica 15/1999, de 13 de diciembre, de protección de datos de carácter personal. En todo momento, Vd. podrá acceder a sus datos, corregirlos o cancelarlos. Sólo el equipo investigador, los monitores del estudio en representación del promotor, y las autoridades sanitarias, que tienen deber de guardar la confidencialidad, tendrán acceso a todos los datos recogidos por el estudio. Se podrá transmitir a terceros información que no pueda ser identificada. En el caso de que alguna información sea transmitida a otros países, se realizará con un nivel de protección de los datos equivalente, como mínimo, al exigido por la normativa de nuestro país.

¿Qué ocurrirá con los cuestionarios obtenidos? Los cuestionarios serán guardados de forma codificada y solo pueden acceder los miembros del equipo investigador y los participantes de la recogida de la documentación y las autoridades sanitarias. Al terminar el estudio, los cuestionarios serán conservados. Si usted accede, los cuestionarios serán conservados para futuros estudios de investigación relacionados con el presente. Si el cuestionario pudiera ser utilizado por otros grupos de investigación, siempre en líneas de investigación relacionadas con la presente, se le solicitaría permiso a usted nuevamente.

¿Existen intereses económicos en este estudio? El investigador no recibirá retribución específica por la dedicación al estudio y usted no será retribuido por participar.

¿Quién me puede dar más información?

Encarnación López Gimeno.

Matrona. ASSIR Eixample Dret. Teléfono 608 16 40 18.

Muchas gracias por su colaboración.

Anexo 3.Consentimiento informado para la participación "Efectividad de una intervención de asesoramiento del plan de nacimiento en mujeres gestantes"

Yo, (Nombre completo) ____, con DNI, ____________________. He sido informada sobre el estudio "Efectividad de una intervención de asesoramiento del plan de nacimiento en mujeres gestantes" que se realiza en las unidades ASSIR Granollers, Mollet del Vallés, Badalona y Eixample Dreta.

He tenido oportunidad de efectuar preguntas sobre el estudio, he recibido respuestas satisfactorias y tengo suficiente información en relación con el estudio.

Entiendo que la participación es voluntaria y puedo abandonar el estudio cuando lo desee y sin tener que avisar previamente, sin que ello afecte a la calidad de mis cuidados. He sido informada de forma clara precisa y suficiente de los siguientes extremos que afectan a los datos personales que se contienen en este consentimiento y en la ficha o expediente que se abra para la investigación: Estos datos serán tratados y custodiados con respeto a mi intimidad y a la vigente normativa de protección de datos. Sobre estos datos me asisten los derechos de acceso, rectificación, cancelación y oposición que podré ejercitar mediante solicitud ante el investigador responsable en la dirección de contacto que figura en este documento. Estos datos no podrán ser cedidos sin mi consentimiento expreso y no lo otorgo en este acto.

Declaro que he leído y conozco el contenido del presente documento, comprendo los compromisos que asumo y los acepto expresamente. Y, por ello, firmo este consentimiento informado de forma voluntaria para manifestar mi deseo de participar en este estudio de INVESTIGACIÓN hasta que decida lo contrario. Al firmar este consentimiento no renuncio a ninguno de mis derechos. Recibiré una copia de este consentimiento para guardarlo y poder consultarlo en el futuro.

Nombre de la mujer   Nombre del/la investigador/a

DNI y Firma   DNI i Firma
