# Supplementary material for: Effectiveness of birth plan counselling based on shared decision making: A cluster randomized controlled trial (APLANT)
Source: PLoS One. 2022 Sep 12;17(9):e0274240. doi: 10.1371/journal.pone.0274240 (PMC9467369; doi:10.1371/journal.pone.0274240)
Supplement: S2 File — (PDF) [file pone.0274240.s005.pdf]

Effectiveness of a counselling intervention in the birth plan in pregnant women

Research Team

Encarnación López-Gimeno

Gloria Seguranyes

Gemma Falguera Puig

Lucia Burgos Cubero

Mercedes Vicente Hernández

Meritxell Angelet Hidalgo

Judit López Pardo

Griselda Vázquez Garreta

Paula Amezcua La Torre

Eva Vela Martínez

Josep María Manresa Domínguez

Collaborating Institutions

Catalan Institute of Health (Catalonia, Spain)

The Foundation University Institute for Primary Health Care Research Jordi Gol i Gurina (IDIAPJGol) (Catalonia, Spain)

University of Barcelona (Barcelona, Catalonia, Spain)

Index:

	page
Abstract	4
Introduction	5
Hypothesis and objectives	7
Methodology	8
Ethical aspects	14
Difficulties and limitations	14
Practical applicability	15
Timeline	16
Bibliography	16
Annexes	26

Abstract

Introduction. The birth plan is the written report of the mother preferences about the management of his childbirth in topics where choices are available because of safety and effectiveness. Birth plans may help to the pregnant women in reaching real expectations about her childbirth, improve her involvement on decision makings, increasing the control on the birth process and reaching a satisfying experience.

Objectives

To evaluate the effectiveness of a counselling intervention in the birth plan, based on “shared decision-making”, in pregnant women in relation to the presentation of the birth plan to the hospital and on preferences regarding aspects related to childbirth and satisfaction with the childbirth, compared to the standard counselling.

To evaluate the effectiveness of a counselling intervention in the birth plan, based on “shared decision-making”, in pregnant women in relation to the degree of information received in the pregnancy about the childbirth, with respect to the standard counselling

Design. A cluster randomised trial study, multicentre, parallel which will reveal the effectiveness of counselling intervention birth plan will be held based on "shared decision-making”.

Participants: Pregnant women with prenatal care in Sexual and Reproductive Health Care (ASSIR) centres and who wish to participate in the study. Sample size: 266 pregnant women will be studied, 133 in each of the groups.

Statistical analysis: Descriptive analysis will be performed in all the variables and a bivariant analysis through hypothesis testing. Confidence interval CI will be settled on 95%. A multivariate analysis, relative risks with a CI of 95%.

Keywords: birth plan, assessment, midwives, satisfaction, shared decision making.

BACKGROUND AND CURRENT STATUS OF THE TOPIC

The experience of the birth of a child is an important event in a woman's life. The birth plan is the written expression of the mother's preferences regarding the management of her childbirth in those aspects in which there are equally effective and safe alternatives (1). Its main purpose is to promote decision-making not influenced by the emotions that arise during the birth process and to provide a vehicle for communication between future parents, the care provider and the hospital team (2). In addition, it helps the pregnant woman to understand her personal values, needs and concerns in the birth process (3). Its preparation can help the pregnant woman to have real expectations of childbirth, involve her in decision-making, thus increasing control over the birth process and have a satisfactory experience with the birth experience (4,5).This document is used to express knowledge of your rights and duties as a user, to be responsible for your health, your legal capacity to consent or not to certain procedures and also ask that your decisions be respected as stated in Spanish Law 41 / 2002 on the autonomy of the patient established in art. 4.1 (6).

The competence framework of the gynaecological obstetric nursing specialty (midwives) includes the care of low and medium risk pregnancy and childbirth, and in its midwives must support pregnant women in the preparation of the birth plan (7). This needs to be approached, by midwives, with strategies that allow future parents to complete it (8). According to Epstein et al, clinical decision-making should be taken from the creation of a collaborative relationship with the patient and family using the best available evidence, consistent with the patient's values, objectives and capacities (9).The use of tools to help patients make decisions regarding routine practice improves the perception of the probabilities of the results, the congruence between the chosen option and the person's values (10).

The available literature on the usefulness and satisfaction of birth plans is scarce and inconclusive. A retrospective study carried out in Murcia revealed that only 2.6% of pregnant women presented the birth plan document to the hospital, and significant differences were also found in the increase in skin-to-skin contact, in delayed clamping of the umbilical cord, in the increase in spontaneous vaginal birth and in the decrease in hospital stay in women who used the birth plan (11). In a study with qualitative methodology carried out in Mexico, all women showed satisfaction with the experience of completing the birth plan even though their experience did not fully conform to what

was specified in the birth plan. The mere fact of writing down their preferences was valued as a more personal and rewarding experience [(4)]. A clinical trial in Taiwan on the birth plan found significant differences in relation to the fulfilment of the expectations of the women in the experimental group that showed an increase in control over the birth process, as well as greater satisfaction in it [(5)]. In contrast, in an experimental study carried out in Sweden, they did not find that carrying out the birth plan improved the birth experience; although it could have beneficial effects in relation to fear of childbirth and pain in women [(12)].

The bibliography also highlights the different points of view that health professionals have on birth plans, in some studies they describe them as useful since they highlight the preferences of women, improve communication with pregnant women and address the concerns of these [(13)].On the other hand, other studies define that the term "birth plan" can be misleading and create false expectations and put pressure on midwives and the multidisciplinary team [(14)].

In Spain in 2007, the National Health System drew up the "Strategy for Attention to Normal Childbirth" to respond to the demand of women to actively participate in their childbirth [(15)] and in the same year in Catalonia, the Catalan Health Institute published the “Protocol for the natural assistance of normal childbirth” [(16)], and both documents include the “birth plan”.

Within the portfolio of services of the Sexual and Reproductive Health Care (ASSIR) in Primary Care in Catalonia, midwives carry out the control and monitoring of pregnancy and postpartum of low and medium risk pregnant women in health centres [(17)] and the birth of women is attended in the reference hospitals.

In 2016, a survey carried out in Catalonia on the satisfaction of women in care in the maternity process showed that the degree of global satisfaction with the care received was high, 8.30 out of 10. However, women report that 67.2%, 64.2%, and 57.6% received sufficient information on pregnancy, birth and postpartum respectively [(18)].

The professionals of the ASSIR and the reference Hospitals jointly developed the birth plan with the aim of allowing women to actively participate in decision-making, and to be a communication instrument that favours the transmission of the wishes of women between primary care and hospital. However, the level of implementation of the birth plan is not known since neither in ASSIR nor in obstetric areas of hospitals is there a record on its presentation and use. In the bibliographic review carried out, not enough

evidence was found of the effectiveness of the advice given by midwives on the birth plan to pregnant women. That is why studies are needed to verify whether standardized counselling in the completion of the birth plan, or counselling based on “shared decision-making”, influences the presentation of birth plans to the hospitals, women’s preferences on aspects of childbirth, satisfaction with the information received and satisfaction with childbirth.

HYPOTHESIS

- Counselling on the birth plan for pregnant women, based on "shared decision making" will increase the presentation of birth plans to the hospital.

- Counselling on the birth plan for pregnant women, based on “shared decision-making” will modify: preferences on aspects related to childbirth, the degree of satisfaction with the information received in the pregnancy on the childbirth and the degree satisfaction in childbirth, in relation to the pregnant women who receive the usual counselling

OBJECTIVES

Main objectives

To evaluate the effectiveness of a counselling intervention in the birth plan, based on “shared decision-making”, in pregnant women in relation to the presentation of the birth plan to the hospital, the preferences regarding aspects related to childbirth and satisfaction with the childbirth, compared to the standard counselling.

To evaluate the effectiveness of a counselling intervention in the birth plan, based on “shared decision-making”, in pregnant women in relation to the information received during the pregnancy about the childbirth, with respect standard counselling.

Secondary objectives

1.- Describe the pregnant women's sociodemographic and obstetrics characteristics.

2.- Analyse if there are differences between the pregnant women of both study groups in the preferences regarding: companion, physical space, delivery procedures, pain relief methods, second stage of birth, the baby and breastfeeding.

3.-Find out the perception of pregnant women on the degree of usefulness of the preparation of the birth plan.

4.- To find out the degree of global satisfaction of pregnant women in childbirth, satisfaction in decision-making in first and second stage childbirth in pregnant women in both study groups.

5.-Determine the reasons for non-presentation of the birth plans to the hospitals of the pregnant women in both study groups.

6. Find out if there are differences, according to the sociodemographic and obstetric characteristics of the pregnant women, in the presentation of birth plans to the hospital, in the preferences of aspects related to childbirth, the degree of information received in the pregnancy about the childbirth and in satisfaction in childbirth in both study groups.

METHODOLOGY

Setting. The study will be carried out in the ASSIR units of Mollet del Vallés, Granollers, Badalona in the province of Barcelona, “Metropolitan Nord” Area and the ASSIR Eixample Dret, “Barcelona City” Area, in Primary Care of the Catalan Institute of Health. In these units, a total of 6,496 first pregnancy visits were made in the year 2015.

The study will last for two years.

Design. An experimental, multicentre, randomized cluster trial and parallel study will be carried out that will allow to know the effectiveness of a counselling intervention of the birth plan based on “shared decision making”.

Subjects

Target population Pregnant women with prenatal care in the health care centres of the ASSIR from Catalonia

Inclusion criteria. Older pregnant women with prenatal care in the centres study, with childbirth in the reference hospitals, who wish to participate in the study and want to fill in the birth plan

Exclusion criteria. Illiteracy, language barrier, drug addiction, alcoholism, grade 2, 3 and 4 heart disease, severe associated maternal pathology, type I-II diabetes, uterine malformation, diagnosed foetal malformation, threatened preterm birth, twin or multiple pregnancy, cervical incompetence, previous perinatal death, intrauterine growth retardation, placenta previa, isoimmunization, mild-severe preeclampsia, premature rupture of membranes, maternal infection.

Sample size. The sample calculation has been made based on the main variable of the study "presentation of birth plans". Based on the descriptive pilot study (n = 211 pregnant women), 48% of the women who receive the usual advice presented the

birth plan to the hospital. It is estimated that this prevalence may be higher in women who receive counselling, around 68%. In this study, a randomized, multicentre and parallel experimental design in clusters is proposed (4 ASSIRs participate). Applying an alpha error of 5%, a beta error of 20% and taking into account the influence of the clusters in the analysis, a total of 133 are required in the control group and 133 in the intervention group. 15% loss to follow-up has been estimated. The calculation has been carried out using the macros of SPSS V22. An interim analysis will be performed to assess whether the sample size needs to be recalculated so that there is sufficient power for the subgroup analysis.

Sampling technique. In the random assignment of ASSIR, the characteristics of the reference hospitals will be taken into account, since they have different levels of care. Thus, two ASSIRs will be assigned to the intervention group and two to the control. For this assignment, the Epidat 4.1 program will be used. This type of assignment has been chosen to avoid contamination among midwives who will or will not receive specific training on birth plan counselling based on shared decision making.

The sampling technique of pregnant women within each ASSIR will be consecutive and proportional to the number of first visits (see table 1).

Table 1. Proportional sampling according to the number of first ASSIR visits

ASSIR	First visits	Proportion	Sample size
Mollet del Vallés	1017	15.1%	41
Badalona	1933	29.6%	79
Granollers	2260	35.7%	94
Dreta Eixample	1286	19.6%	52
Total	6496	100%	266

Intervention

Characteristics of the intervention in the control group.

Midwives in the prenatal control visit between 24 to 28 weeks of gestation will deliver the birth plan so that the pregnant woman can fill it out at her home. In the prenatal control visit between 29 to 33 weeks of gestation, the pregnant women will deliver the birth plan to the midwife and the pregnant women will be able to ask about the aspects they want. The midwives will then deliver the second birth plan to the pregnant women so that they can fill it out at home and then they must deliver a copy

to the midwife at the prenatal visit between 34 and 40 weeks of gestation.

The midwife will instruct the pregnant woman to present the original to the reference hospital.

Characteristics of the intervention in the intervention group

Phase I. Training intervention for midwives. There will be a 4-hour workshop on counselling in the birth plan, based on the communicative intervention of negotiation of options in shared decision making by Epstein et al, which consists of: understanding the experience and expectations of the pregnant woman, construction of a collaborative relationship between the pregnant woman and the professional, provide information according to the existing level of evidence and current recommendations, and verification of understanding and compliance [(9)]

Phase II. Intervention in pregnant women. The pregnant women will receive the birth plan from the midwives in the prenatal control visit between 24-28 weeks of gestation that they will complete at home. Later, in the prenatal control visit between 29-33 weeks of gestation, the pregnant women will receive the counselling intervention from the midwife, which will consist of standardized advice on the birth plan based on shared decision making according to Epstein et al [(9)] and in the delivery of an informative leaflet [(19)] based on the existing scientific evidence of the aspects expressed in the birth plan. The counselling of the midwife of the pregnant women will include the following activities: it will explore the previous experiences and expectations of the pregnant woman regarding the childbirth; establish with the pregnant woman a collaborative relationship in decision-making; explain the existing evidence for each aspect of the birth plan; will inform of the updated recommendations on the aspects specified in the birth plan and will check the understanding of the information provided to the pregnant woman. The midwife will then deliver a second birth plan that the pregnant woman will fill out at her home and that she must deliver a copy to the midwife at the prenatal visit between 34-40 weeks of gestation. The midwife will instruct the pregnant woman to present the original to the reference hospital.

Variables

Independent variables

Counselling intervention activity based on shared decision making in the birth plan

Delivery of information leaflet: yes, no

Sociodemographic and obstetric variables

- Nationality: country of origin.
- Age calculated from the date of birth, years, and months.
- Educational level: without studies; primary studies; secondary studies; university studies.
- Employment: yes, no.
- Live with your partner: yes, no.
- Previous births: yes, no.
- Maternal education: yes, no.
- Internet use for seeking information: yes, no.

Outcome variables

Variables related to the birth plan

- Presentation of the birth plan to the hospital: yes, no.
- Reasons of non-presentation to the birth plan: I did not think it was necessary, I forgot, the professionals who attended me did not ask me, others.
- Usefulness of completing the birth plan in decision-making on aspects of childbirth. On an interval scale from 1 to 5. 0 is not useful at all and 5 is very useful.
- Sufficient information received during the pregnancy about the birth: yes, no
- Use of the birth plan in the next pregnancy: yes, no
- Common preferences on aspects of childbirth expressed in the birth plans: yes, no.
- Companion.
- Physical space: unique space, light graduation.
- Comfort measures and support for childbirth: listen to music, birthing ball.
- Drink fluids during labour.
- Foetal monitoring: continuous, intermittent.
- Freedom of movement.
- Pain relief methods: try labour without anaesthesia, epidural, relaxation techniques, breathing techniques, massage, others non-pharmacological

alternatives methods.

- Birth: directed pushing, spontaneous pushing, choose birthing position, use of the mirror for the birth,
- Baby: Early skin to skin in the delivery room, delayed clamping of the umbilical cord, breastfeeding delivery room, I do not want to breastfeed, consult my opinion before giving food to the baby
- Change of preferences regarding aspects of childbirth.

Obstetric variables:

- Onset of labour: spontaneous, induced
- Type of birth: spontaneous vaginal, operative birth, caesarean section
- Episiotomy: yes, no.
- Type of analgesia: non-pharmacological, pharmacological, both.
- Non-pharmacological analgesia: relaxation, breathing techniques, massage, use of water, local heat, birthing ball, TENS, others: acupuncture, aromatherapy, homeopathy, Bach flowers.
- Pharmacological: epidural
- Early of skin to skin in the delivery room: yes, no.
- Initiation of breastfeeding in the delivery room: yes, no.
- Perinatal complications: no, yes.
- Maternal complications: no, yes.

Satisfaction with the childbirth experience

- Satisfaction in childbirth. Using the MacKey scale of satisfaction at childbirth [20]. On a scale of 1 to 5. 1 very dissatisfied, 2 not very satisfied, 3 indifferent, 4 satisfied, 5 very satisfied. The total score on the scale and on the subscales of degree of participation in decision-making in dilation and delivery will be considered.

Data collection and tools

Birth plan. It is the document included in the Protocol for natural assistance to normal childbirth of the Catalan Health Department [16]. It consists of items grouped into sections that refer to preferences about companion, physical space, procedures, pain relief methods, aspects related to first and second stage of birth, postpartum and lactation. The woman completes the birth plan and can choose more than one option

in each section. The birth plans are agreed upon between ASSIR and the referral hospital according to the specific characteristics of each Hospital.

MacKey Childbirth Satisfaction Scale. Questionnaire validated into Spanish with an interclass coefficient of 0.93 for the global scale and with a Cronbach's alpha reliability greater than 0.7 in all subscales (20). It consists of 35 items. Each item is valued on a Likert scale, 1 as very dissatisfied, 2 not very satisfied, 3 indifferent, 4 satisfied, 5 very satisfied. The global score is obtained by adding the values assigned to each item. A higher score means greater satisfaction.

Sociodemographic and obstetric data sheet. Prepared by the research team, it consists of sociodemographic data and variables related to childbirth.

Presentation of the birth plan to the hospital data sheet. It consists of four closed questions: received enough information on the pregnancy about the birth, presented the birth plan to the hospital, if not, what was the cause, a useful question of completion of the birth plan in the decision-making in the childbirth that was valued on a scale of 0 to 5, and a question if they would use the birth plan in the next pregnancy.

Data collection procedure

The midwives of the ASSIR will carry out the recruitment of the pregnant women in the control and intervention group in the prenatal visit of 24-28 weeks of gestation and they will be explained what the study consists of verbally and in writing. If they wish to participate, the informed consent will be collected and then the midwife will collect the sociodemographic and obstetric data and will deliver the birth plan for the pregnant women to fill in at home.

In the prenatal visit of 29-33 weeks of gestation, midwives will collect the completed birth plans and will deliver a new birth plan that the women will fill out at home and will be instructed to deliver a copy at the prenatal visit between the 34-40 weeks of gestation.

The midwife will keep a copy and will indicate to the pregnant women that the original must be presented to the hospital. In the women in the intervention group, the midwives will carry out the standardized counselling intervention in the preparation of the birth plan based on the shared decision making of Epstein et al and in the pregnant women in the control group, the midwives will carry out the usual practice of counselling the plan of birth.

In the postpartum visit between hospital discharge and the 6th postpartum week, the midwives of the control and intervention group will collect the childbirth data and deliver an sealed envelope with the questionnaire of questions about the childbirth and the birth plan, sufficient information satisfaction received during pregnancy about the delivery, usefulness of completing the birth plan, intention to use it in the next pregnancy and the MacKey questionnaire on the satisfaction of childbirth.

Pilot test. A pilot test will be carried out with 10 pregnant women in each ASSIR.

Analysis.

A descriptive analysis of all variables will be carried out. The categorial ones with frequencies and percentages; the quantitative ones with mean and standard deviation and if they do not comply with a normal distribution, the median, minimum, and maximum will be observed. The statistical software package SPSS 22.0 will be used. Homogeneity tests between the groups will be performed for the main sociodemographic variables to analyse the comparability of the groups. Hypothesis contrast tests, Chi-square test and Student's t test will be performed. A statistical significance level of 5% will be used. Confidence level will be settled on 95%. A multivariate analysis, relative risks and number of subjects will be calculated with a confidence interval of 95%.

Bibliographic search. The databases consulted for the search were MEDLINE; Cochrane; EBSCO; CINAHL and CUIDEN from 2000 to 2016 Keywords: birth plans, assessment, midwife, satisfaction, decision making.

Ethical aspects. The principles of the Declaration of Helsinki (21) will be considered and the necessary precautions will be taken to minimize the possible repercussions on pregnant women during the investigation according to the recommendations of the Belmont report. (22) Authorization will be requested from the Committee on Clinical research ethics of the Jordi Gol i Gorina Foundation for the ASSIR of the Catalan Institute of Health. The information sheet and informed consent is common for participants in the control and intervention group. Throughout the process, the confidentiality of the data will be guaranteed according to Organic Law 15/1999, on the protection of confidential data (23).

Difficulties and limitations. Women can also access birth plan information through other pregnant women or other means such as the internet that could modify decisions regarding it. To avoid contamination among midwives in the counselling

activity, a randomization of ASSIR has been chosen. The possible losses that may be due to a change in the risk of gestation after 29 weeks that limit following the completed birth plan and/or require a childbirth in a hospital other than that of reference. In situations where neonatal death occurs, the degree of satisfaction will not be evaluated.

Applicability and practical utility. Advice on shared decision making in the birth plan can incorporate improvements in conscious decision-making and greater autonomy in pregnant women, improving the information received during pregnancy on aspects of their childbirth. It can incorporate quality indicators on the care received and satisfaction in primary care and hospital.

Timeline

	September 2016	October-December 2016	February- April 2017	March- June 2017	May 2017	June-July 2017	September 2017	October 2017	November 2017 -February 2019
Bibliographic review									
Requests and authorizations									
Preparation of a dossier for the midwives of the intervention group on the scientific evidence on aspects that appear in the birth plans									
Preparation of the provisional leaflet									
Leaflet consensus meetings									
Final design of the leaflet									
Training workshop for midwives of the intervention group									
Test pilot									
Data Collect									

Bibliography

1. Junta de Andalucía. Plan parto y nacimiento. Sevilla: Junta de Andalucía, Consejería de Salud; 2009.
2. Kaufman T. Evolution of the birth plan. J Perinat Educ. 2007; 16(3):47-52.
3. Bailey JM, Crane P, Nugent CE. Childbirth education and birth plans. Obstet Gynecol Clin North. 2008; 35(3): 497-509.
4. Yam EA, Grossman AA, Goldman LA, Garcia SG. Introducing birth plans in Mexico: an exploratory study in a hospital serving low-income Mexicans. Birth. 2007; 34(1):42-8.
5. Kuo SC, Lin KC, Hsu CH, Yang CC, Chang MY, Tsao CM, Lin LC. Evaluation of the effects of a birth plan on Taiwanese women's childbirth experiences, control and

expectations fulfillment: A randomized controlled trial. Inter J Nurs Stud. 2010; 47(7): 806-14.

6. Ley 41/2002 de 14 noviembre básica reguladora de la autonomía del paciente y de derechos y obligaciones en materia de información y documentación clínica. Boletín oficial del estado nº 274, (15-11-2002).
7. Orden SAS/1350/2009, de 6 de mayo, por la que se aprueba y publica el programa formativo de la especialidad de Enfermería Obstétrico-Ginecológica. Boletín oficial del estado nº129, (28-05-2009).
8. Anderson CJ, Kilpatrick. Supporting patients' birth plans: theories, strategies & implications for nurses. Nurs Womens Health. 2012; 16(3):210-8.
9. Epstein RM, Alper BS, Quill TE. Communicating evidence for participatory decision making. JAMA. 2004; 291(19): 2359-66.
10. Stacey D, Légaré F, Col NF, Bennett CL, Barry MJ, Eden KB, Holmes-Rovner M, Llewellyn-Thoma. Decision aids for people facing health treatment or screening decision. Cochrane Database of Systematic Reviews 2014, Issue 1.
11. Suarez M, Armero D,Canteras, M, Martinez E. Use and influence of Deliery and Birth Plans in the humanizing delivery process. Rev. Latino-Am. Enfermagem. 2015; 23(3): 520-6.
12. Lundgren I, Berg M, Lindmarck G. Is the childbirth experience improved by a birth plan? J Midwifery Womens Health. 2003; 48 (5): 322-8
13. Whitford HM1, Entwistle VA, van Teijlingen E, Aitchison PE, Davidson T, Humphrey T, Tucker JS. Use of a birth plan within woman-held maternity records: a qualitative study with women and staff in northeast Scotland. Birth. 2014;41(3):283-9.
14. Welsh JV, Symon AG. Unique and proforma birth plans: a qualitative exploration of midwives′ experiences. Midwifery. 2014;30(7):885-91
15. Ministerio de Sanidad y Consumo. Estrategia de Atención al Parto Normal en el Sistema Nacional de Salud. Madrid: Ministerio de Sanidad y Consumo; 2007.
16. Generalitat de Catalunya. Protocol per a l’ assistència al part normal. Barcelona: Generalitat de Catalunya, Departament de Salut; 2007.
17. Direcció General de Planificació Sanitaria i Avaluació. Cartera de Serveis de les Unitats d’Atenció a la Salut Sexual i Reproductiva de suport a l’ Atenció Primària. Barcelona: Generalitat de Catalunya, Departament de Salut; 2007.

18. Divisió d'Atenció al Ciutadà. Qualitat de servei i satisfacció. Estudi de qualitat de servei i satisfacció amb l'embaràs, part i postpart. Barcelona: Generalitat de Catalunya, Servei Català de la Salut; 2016.

19. Dugas M, Shorten A, Dubé E, Wassef M, Bujol E, Chaillet N. Decision aid tools to support women's decision making in pregnancy and birth: a systematic review and meta-analysis. Soc Sci Med. 2012 ;74(12):1968-78

20. Mas R, Barona C, Carregui S, Ibáñez N, Margaix L, Escriba V. Satisfacción de las mujeres con la experiencia del parto: validación de la Mackey Satisfacción Childbirth Rating Scales. Gac Sanit .2012; 26(3):236-42.

21. World Medical Association Declaration of Helsinki. Ethical Principles for Medical Research Involving Human Subjects. Seoul: 59th WMA General Assembly; 2008.

22. The National Commission for protection of Humans subjects of biomedical and Behavioral Research. Ethical principles and guidelines for the protection of human subject research. Belmont; 1978.

23. Ley Orgánica de Protección de Datos de Carácter Personal. Ley 15/1999, de 13 de diciembre. Boletín oficial del estado nº298, (14-12-1999).

24. Simkin P. Birth Plans: After 25 years, women still want to be heard. Birth. 2007; 34(1): 49-51.

25. Lothian J. Birth Plans: The good, the bad, and the future. J Obstet Gynecol Neonatal Nurs. 2006; 35 (2): 295-303.

26. Say R, Thomason R. The importance of patient preferences in treatment decisions challenges for doctors. BMJ. 2003; 327(6):542–5.

27. Woolf S, MD, Chan E C, Harris R, Sheridan S L, Braddock C.H, Kaplan R.M et al. Promoting Informed Choice: Transforming Health Care To Dispense Knowledge for Decision Making. Ann Intern Med. 2005; 143(4):293-300.

28. Hadjigeorgiou E, Kouta C, Papastavrou E, Papadopoulos I, Mårtensson LB. Women's perceptions of their right to choose the place of childbirth: an integrative review. Midwifery. 2012; 28(3):380-90.

29. Roberts CL, Raynes-Greenow CH, Nassar N, Trevena L, McCaffery K. Protocol for a randomised controlled trial of a decision aid for the management of pain in labour and childbirth. BMC Pregnancy Childbirth. 2004; 4(1): 24.

30. Eden KB, Dolan JG, Perrin NA, Kocaoglu D, Anderson N, Case J, Guise JM. Patients were more consistent in randomized trial at prioritizing childbirth preferences using graphic-numeric than verbal formats. J Clin Epidemiol. 2009; 62(4):415-424.

31. Melo-Martin I, Intemann K. Interpreting Evidence: Why values can matter as much as Science. Perspect Biol Med. 2012; 55(1):59-70.
32. Chin G, Warren N, Kornman L, Cameron P. Patients' perceptions of safety and quality of maternity clinical handover. BMC Pregnancy Childbirth. 2011; 11:58.
33. PennellA ,Salo-Coombs V, Herring A, Spielman F, Fecho K. Anesthesia and analgesia-related preferences and outcomes of women who have birth plans. J Midwifery Womens Health. 2011;56
34. Grant R, Sueda A, Kaneshiro B. Expert opinion vs. patient perception of obstetrical outcomes in laboring women with birth plans. J Reprod Med. 2010; 55(1-2):31-5.
35. Carlton T, Callister L, Christiaens G, Walker D. Labor and delivery nurses' perceptions of caring for childbearing women in nurse-managed birthing units. MCN Am J Matern Child Nurs.2009; 34(1):50-6.
36. Demontis R, Pisu S, Pintor M, D' aloja E. Cesarean section without clinical indication versus vaginal delivery as a paradigmatic model in the discourse of medical setting decisions. J Matern Fetal Neonatal Med. 2011; 24(12):1470-5.
37. Romano AM, Lothian JA. Promoting, Protecting, and Supporting Normal Birth: A Look at the Evidence. J Obstet Gynecol Neonatal Nurs. 2008; 37(1):94-104.
38. National Institut for Health and Clinical Excellence Antenatal Care. Clinical guideline. Routine care for the healthy pregnant woman. London;
39. Raynes-Greenow CH, Roberts CL, McCaffery K, Clarke J. Knowledge and decision-making for labour analgesia of Australian primiparous women. Midwifery. 2007; 23(2):139-45.
40. Declercq E, Sakala C, Corry MP, Applebaum S, Herrich A. Listening to mothers III:
41. Pregnancy and childbirth. Report. New York: Childbirth Connection;2013 [acceso 25 de mayo de 2013]. Disponible en: http://www.maternitywise.org.
42. Deering SH, Zaret J, McGaha K, Satin AJ. Patients presenting with birth plans in a military tertiary care hospital: a descriptive study of plans and outcomes.Mil Med. 2006; 171(8):778-80.
43. Melender HL. What constitutes a good childbirth? A qualitative study of pregnant finnish women. J Midwifery Womens Health.2006; 51:331-9.
44. Philepsen N, Haynes D. The similarities between birth plans and living wills. J Perinat Educ.2005; 14(4):46–8.
45. Chalmers B, Porter R. Assessing Effective Care in Normal Labor: The Bologna Score. Birth.2011; 28:79–83.

46. Cleveland G. Shields, PhD, Franks , Fiscella K MD, Meldrum S, Epstein RM. Rochester Participatory Decision-Making Scale (RPAD): Reliability and Validity. Ann Fam Med. 2005; 3(5): 436–42.
47. Henry A, Nand SL. Women's antenatal knowledge and plans regarding intrapartum pain management at the Royal Hospital for Women. Aust N Z J Obstet Gynaecol. 2004; 44(4):314-7.
48. Shorten A, Chamberlain M, Shorten B, Kariminia A. Making choices for childbirth: development and testing of a decision-aid for women who have experienced previous caesarean. Patient Educ Couns. 2004; 52(3):307-13.
49. Madi BC, Crow R. A qualitative study of information about available options for childbirth venue and pregnant women's preference for a place of delivery. Midwifery. 2003; 19(4):328-36
50. Berg M, Lundgren I, Lindmark G. Childbirth Experience in Women at High Risk: Is It Improved by Use of a Birth Plan? J Perinat Educ. 2003; 12(2):1-15
51. Brooks H, Sullivan W. The importance of patient autonomy at birth. Int J Obstet Anesth. 2002; 11(3):196-203.
52. Hodnett ED. Pain and women’s satisfaction with experience of childbirth: a systematic review. Am J Obstet gynecol.2002;186:160-72
53. Lothian JA. Why Natural Childbirth? J Perinat Educ. 2000; 9 (4): 44-6.
54. Deering SH, Zaret J, McGaha K, Satin AJ. Patients presenting with birth plans: a case-control study of delivery outcomes. J Reprod Med. 2007 ;52(10):884-7.
55. Aragon M, Chhoa E, Dayan R, Kluftinger A, Lohn Z, Buhler K. Perspectives of expectant women and health care providers on birth plans. J Obstet Gynaecol Can. 2013 ;35(11):979-85.
56. Hadar E, Raban O, Gal B, Yogev Y, Melamed N.Obstetrical outcome in women with self-prepared birth plan. J Matern Fetal Neonatal Med. 2012;25(10):2055-7.
57. Whitford HM, Entwistle VA, van Teijlingen E, Aitchison PE, Davidson T, Humphrey T, Tucker JS. Use of a birth plan within woman-held maternity records: a qualitative study with women and staff in northeast Scotland. Birth. 2014;41(3):283-9.
58. Brauer S.Moral implications of obstetric technologies for pregnancy and motherhood. Med Health Care Philos. 2016 Mar;19(1):45-54
59. Mei JY, Afshar Y, Gregory KD, Kilpatrick SJ, Esakoff TF. Birth Plans: What Matters for Birth Experience Satisfaction. Birth. 2016 ;43(2):144-50.

60. Cook K. The impact of choice and conrol on women's childbirth experiences.J Perinat Educ.,21(3),158-168

61. Beatriz Elena Delgado-García, M Isabel Orts-Cortés, Alberto Poveda-Bernabeu, Pablo Caballero-Pérez, Ensayo clínico controlado y aleatorizado para determinar los efectos del uso de pelotas de parto durante el trabajo de parto, Enfermería Clínica, Volume 22, Issue 1, January–February 2012, Pages 35-40, ISSN 1130- 8621, http://dx.doi.org/10.1016/j.enfcli.2011.07.001.

62. Grupo de trabajo de la Guía de Práctica Clínica sobre la atención al parto normal. Guía de Práctica Clínica sobre la atención al parto normal. Plan de Calidad para el Sistema Nacional de Salud del Ministerio de Sanidad y Política Social. Agencia de Evaluación de Tecnologías Sanitarias del País Vasco (OSTEBA). Agencia de Evaluación de Tecnologías Sanitarias de Galicia (Avalia-t). 2010. Guías de Práctica Clínica en el SNS: OSTEBA Nº 2009/01.

63. Plan de parto y nacimiento. Agencia de Calidad del Sistema Nacional de Salud. 2012. [Acceso 1-07-2016](Disponible en: www.msssi.gob.es/organizacion/sns/planCalidadSNS/pdf/.../planPartoNacimiento. pdf

64. Guia NICE. Intrapartum care: care of healthy women and their babies during childbirth. [Acceso 1-07-2016](Disponible en: https://www.nice.org.uk/news/article/midwife-led-units-safest-for-straightforward- births

65. Lemos A, Amorim MMR, Dornelas de Andrade A, de Souza AI, Cabral Filho JE, Correia JB. Pushing/bearing down methods for the second stage of labour. Cochrane Database of Systematic Reviews 2015, Issue 10. Art. No.: CD009124. DOI:10.1002/14651858.CD009124.pub2

66. Romero ME, Carrizosa A, Francisco J. Las posturas de la mujer de parto en fase de expulsivo: revisión de la evidencia científica y recomendaciones. Med. Naturista. 2014; 8 (1): 23-30.

67. Becerra-Maya Emilio José, Lapuente-Jambrina Gloria, Alonso-Ortega Verónica María. Uso del espejo en el periodo expulsivo del parto: evaluación mediante diferencial semántico. Index Enferm [Internet]. 2011 Jun [citado 2016 Ago 09] ; 20(1-2): 46-50. Disponible en: http://scielo.isciii.es/scielo.php?script=sci_arttext&pid=S1132-12962011001100110&lng=es

68. Ballesteros-Meseguer C, Carrillo-García C, Meseguer-de-Pedro M, Canteras- Jordana M, Martínez-Roche ME. Episiotomy and its relationship to various clinical variables that influence its performance. Rev. Latino-Am. Enfermagem. 2016;24:e2793.

[Access03/07/2016]; Available in: http://www.scielo.br/pdf/rlae/v24/es_0104-1169-rlae-24-02686.pdf . DOI: http://dx.doi. org/10.1590/1518-8345.0334.2686.

69. Carroli G, Mignini L. Episiotomía para el parto vaginal. Cochrane Database of Systematic Reviews 2012 Issue 11. Art. No.: CD000081. DOI: 10.1002/14651858.CD000081

70. Devane D, Lalor JG, Daly S, McGuire W, Smith V. Cardiotocography versus intermittent auscultation of fetal heart on admission to labour ward for assessment of fetal wellbeing. Cochrane Database Syst Rev. 2012 Feb 15; (5): CD005122.

71. Alfirevic Z, Devane D, Gyte GM. Continuous cardiotocography as a form of electronic fetal monitoring (EFM) for fetal assessment during labour. Cochrane Database Syst Rev. 2013 May 31; (5): CD006066.

72. Cahill AG, Spain J. Intrapartum fetal monitoring. Clinical Obstetrics and Gynecology. 2015 Jun; 58 (2): 263-8.

73. NICE guidelines. Intrapartum care pathway. Last updated: 09 December 2015

74. Michikata K, Sameshina H, Urabe H, Tokunaga S, Kodama Y, Ikenoue T. The regional centralization of Electronic Fetal Heart Rate Monitoring and its impact on Neonatal Acidemia and the Cesarean Birth Rate. J Pregnancy. 2016; 2016: 3658527.

75. Boatin AA, Wylie BJ, Goldfarb I, Azebedo R, Pittel E, Ng C, Haberer JE. Wireless vital sign monitoring in pregnant women: a functionality and acceptability study. Telemed J E Health. 2016 Jul; 22 (7): 564-71.

76. FAME. Iniciativa parto normal. Documento de consenso. 2007. FAME

77. Walter V, Nelly LF. Anales de la Facultad de Medicina. 2008; 69 (2): 127-9. Smyth RMD, Markham C, Dowswell T. Amniotomy for shortening spontaneous labour (Review). Cochrane Database of Systematic Reviews 2013, Isuue 6. Art. No.: CD006167.

78. Wei S, Wo BL, Qi HP, Xu H, Luo ZC, Roy C, Fraser WD. Early amniotomy and early oxytocin for prevention of, or therapy for, delay in first stage spontaneous labour compared with routine care (Review). Cochrane Database of Systematic Reviews 2013, Isuue 8. Art. No.: CD006794. Ghafarzadeh M, Moeininasab S, Namdari M. Effect of early amniotomy on dystocia risk and cesarean delivery in nulliparous women: a randomized clinical trial. Arch Gynecol Obstet. 2015 Aug; 292 (2): 321-5.

79. Reche DM, Guedes Ch, Galindo A. Factores que influyen en la distocia de un parto de inicio espontáneo. [monografía en Internet] *. Almería: Paraninfo Digital; 2015 [acceso 16 de Julio de 2016]. Disponible en: http://www.index- f.com/para/n22/129.php.

80. Harrison MJ, Kushner KE, Benzies K, Rempel G, Kimak C. Women's satisfaction with their involvement in health care decisions during a high-risk pregnancy. Birth. 2003; 30 (2): 109-15.
81. Biurrun-Garrido A, Goberna-Tricas J. La humanización del trabajo de parto: necesidad de definir el concepto (Revisión). Matronas Prof. 2013; 12 (2): 62-66.
82. Reveiz L, Gaitán HG, Cuervo LG.Enemas during labour. Cochrane Database Syst Rev. 2013 Jul 22;(7):CD000330. doi: 10.1002/14651858.CD000330.
83. Kelly AJ, Kavanagh J, Thomas J. Castor oil, bath and/or enema for cervical priming and induction of labour. Cochrane Database Syst Rev. 2013 Jul 24; (7):CD003099. doi: 10.1002/14651858.CD003099.
84. Kovavisarach E, Sringamvong W.Enema versus no-enema in pregnant women on admission in labor: a randomized controlled trial. J Med Assoc Thai. 2005 Dec; 88(12):1763-7.
85. Tzeng YL, Shih YJ, Teng YK, Chiu CY, Huang MY. Enema prior to labor: a controversial routine in Taiwan.J Nurs Res. 2005 Dec;13 (4):263-70.
86. Barbosa da Silva, Flora Maria et al. Prácticas para estimular el parto normal.
87. Index Enferm, Set 2011, vol.20, no.3, p.169-173. ISSN 1132-1296
88. Kovavisarach E, Jirasettasiri P. Randomised controlled trial of
89. perineal shaving versus hair cutting in parturients on admission in labor. J Med Assoc Thai. 2005 Sep;88 (9):1167-71.
90. Basevi V, Lavender T.Routine perineal shaving on admission in labour. Cochrane Database Syst Rev. 2014 Nov 14;(11):CD001236. doi: 10.1002/14651858.CD001236.pub2.
91. Gupta JK, Hofmeyr GJ. Posición de la mujer durante el período expulsivo del trabajo de parto (Revisión Cochrane traducida). En: La Biblioteca Cochrane Plus. 2008; 4. Oxford: Update Software Ltd.
92. Roberts CL, Algert CS, Olive E. Impact of first-stage ambulation on mode of delibery among women with epidural analgesia. Aust N Z J Obstet Gynaecol 2004;446:489-94.
93. Souza JP, Miquelutti MA, Cecatti JG, Makuch MY. Maternal position during the first stage of labor: A systematic review. Reproductive Health 2006; 310.
94. Lawrence A, Lewis L, Hofmeyr GJ, Dowswell T, Styles C. Maternal positions and mobility during first stage labour. Cochrane Database Syst Rev 2009

95. Ben Regaya L, Fatnassi R, Khlifi A, Fékih M, Kebaili S, Soltan K, Khairi H, Hidar S Role of deambulation during labour: A prospective randomized study. J Gynecol Obstet Biol Reprod (Paris). 2010 Dec;39(8):656-62. doi: 10.1016/j.jgyn.2010.06.007

96. Souza JP, Miquelutti MA, Cecatti JG, Makuch MY Maternal position during the first stage of labor: a systematic review.Reprod Health. 2006 Nov 30; 3:10.

97. O 'Sullivan G, Liu B, Hart D, Seed P, Shennan A. Effect of food intake during labour on obstetrics outcome: randomised controlled trial. BMJ 2009;338-b784.

98. Singata M,Tranmer J, Gyte GM. Restricting oral fluid and food intake during labour. Cochrane Database Syst Rev 2010;(1):CD003930.

99. Scheepers HC, De Jong PA, Essed GG, et.al. Carbohydrate solution intake during labour just before the start of the second stage: a double-blind study on metabolic effects and clinical outcome. BJOG 2004; 111(12):1382-7.

100. Smith RB, Toledano MB, Wright J, Raynor P, Nieuwenhuijsen MJ.Tap water use amongst pregnant women in a multi-ethnic cohort.Environ Health. 2009 Dec 21;8 Suppl 1:S7. doi: 10.1186/1476-069X-8-S1-S7.

101. Saxton A, Fahy K. Effects o skin-to.skin contact and brestfeeding at birth on the incidence of PPH: A physiologically bases theory.Volume 27, Issue 4, Desember 2014, Pages 250-253.

102. Redshaw M, BA, PhD, Hennegan J, Kruske S.Holding the baby: Early mother- infant contact after childbirth and outcomes. Midwifery 30 (2014) 177-187. doi: 10.1016/j.midw.2014.02.003. Epub 2014 Feb 22

103. Phillips R Sacred Hour: Uninterrupted Skin-to-Skin Contact Immediately After Birth Newborn and Infant Nursing Reviews 13 (2013) 67-72

104. Moore ER, Anderson Gc, Bergman N, Dowxwell T. Early skin-to-skin contact for mother s and their healthy nexborn infants (review)Cochrane Database of Systematic Reviews 2012, Issue 5. Art. Nº : CD 003519. DOI: 10.1002/14651858. CD003519. Pub3

105. Dois A C, Luchhchini C, Villarroel L, Uribe C. Efecto del contacto piel con piel sobre la presencia de síntomas depresivos post parto en mujeres de bajo riesgo obstétrico. Rev Chil Pediatr 213; 84 (3) : 285-292

106. Lucchini C, Marquez F , Uribe C.Efectos del contacto piel con piel del recién nacido con su madre.Index Enferm [Internet]. 2012 Dic [citado 2016 Sep 26] ; 21(4): 209-213. Disponible en: http://scielo.isciii.es/scielo.php?script=sci_arttext&pid=S1132-12962012003300007&lng=es

Annexes

Annex 1. Protocol study flow chart

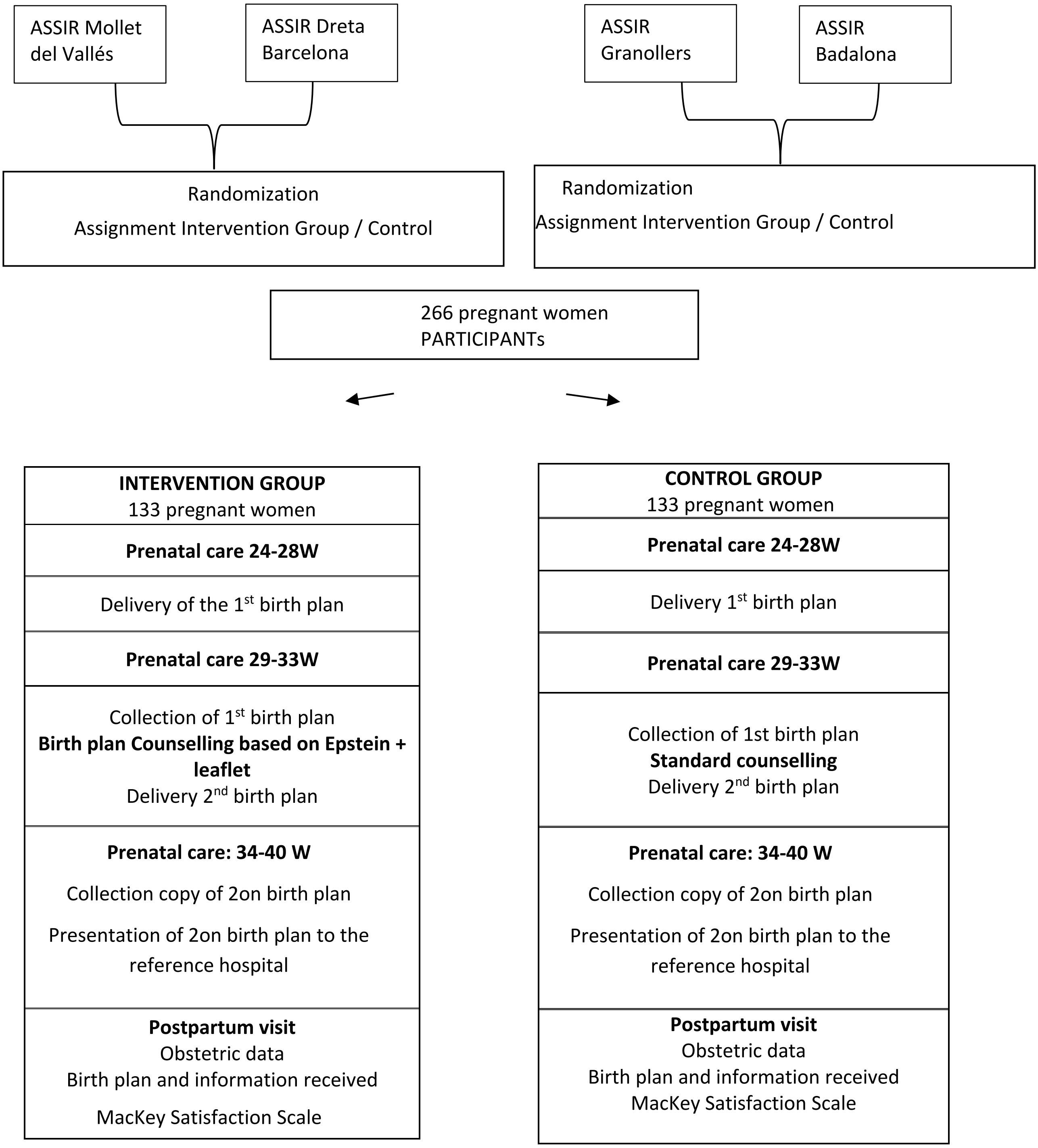

Annex 2. Information sheet for the participant in the study "Effectiveness of a birth plan counselling intervention in pregnant women"

This document is intended to provide you with information about a research study in which you are invited to participate. It is carried out in different Centres for Sexual and Reproductive Health Care (ASSIR) in the Region and was approved by the Ethical Committee in Clinical Research of the Jordi Gol Institute for Primary Care Research (IDIAP). If you decide to participate in it, you will be receiving personalized information from the midwife, read this document beforehand and ask all the questions that she needs to understand the details about it. If you wish, you can bring the document, consult it with others, and take the time to decide whether to participate or not. Participation in this study is completely voluntary. You can decide not to participate or, if you agree to do so, change her mind by withdrawing consent at any time without obligation to explain. We assure you that this decision will not affect the relationship with her midwife or the health care to which she is entitled.

What is the purpose of the study? This study has purpose to evaluate the effectiveness of a birth plan counselling intervention in pregnant women. You are invited to participate for evaluate the effectiveness of a counselling intervention in the birth plan in pregnant women. Collaboration and participation in this study are aimed at improving the care received from health services.

Why do you offer to participate to me? The selection of people invited to participate depends on criteria that are described in the research protocol. These criteria serve to select the population in which the research question will be answered. You are invited to participate because you meet these criteria. A total of 266 women are expected to participate.

What does my participation consist of? Your participation will consist of filling in the birth plans during pregnancy and anonymous surveys that will be delivered to you by the midwife who makes the postpartum visit on aspects related to your delivery and the birth plan. In no case will the protocolized control of your care in pregnancy and the puerperium diminish. In case new information is needed, we can contact you. The expected duration of your participation in the study will be approximately 8 months.

What risks or drawbacks does it have? Spend your time answering the surveys

Will I get any benefits for participating? You are not expected to derive direct benefit from participating in the study. The use of birth plans is unknown, so we want to investigate. The only benefit sought to know the situation of the use of birth plans in order to make possible improvements in health care in the future.

Will I receive the information obtained from the study? If you wish, a summary of the study results will be provided to you. You can also have the completed questionnaires, if you request it.

Will the results of this study be published? The results of this study will be published in scientific journals for dissemination, but no data that may lead to the identification of the participants will be transmitted.

How will the confidentiality of my data be protected? The treatment, communication and transfer of your data will be done in accordance with the provisions of Organic Law 15/1999, of December 13, on the protection of personal data. At any time, you can access your data, correct it or cancel it. Only

the research team, the study monitors on behalf of the promoter, and the health authorities, who have a duty to keep confidentiality, will have access to all the data collected by the study. Information that cannot be identified may be transmitted to third parties. In the event that any information is transmitted to other countries, it will be carried out with a level of data protection equivalent, at least, to that required by the regulations of our country.

What will happen to the questionnaire obtained? The questionnaires will be stored in a coded form and can only be accessed by members of the research team and participants in the collection of documentation and the health authorities. At the end of the study, the questionnaires will be kept. If you agree, the questionnaires will be retained for future research studies related to this. If the questionnaire could be used by other research groups, always in lines of research related to this one, your permission would be requested again.

Are there financial interests in this study? The researcher will not receive specific rewards for dedication to the study and you will not be rewarded for participating.

Who can give me more information?

Encarnación López Gimeno.

Midwife ASSIR Eixample Dret. Mobile.

Thank you very much for your help.

Annex 3. Informed consent for participation "Effectiveness of a birth plan counselling intervention in pregnant women"

I, (XXXXXXXXXX), with ID, XXXXXXXXX I have been informed about the study "Effectiveness of a birth plan counselling intervention in pregnant women" that is carried out in the ASSIR Granollers, Mollet del Vallés, Badalona and Barcelona Dreta

I have had the opportunity to ask questions about the study,I have received satisfactory answers, and I have sufficient information regarding the study.

I understand that participation is voluntary, and I can leave the study whenever I want and without having to give prior notice, without affecting the quality of my care. I have been informed in a clear, precise and sufficient way of the following points that affect the personal data contained in this consent and in the file or file that is opened for investigation: These data will be treated and guarded with respect to my privacy and current data protection regulations. I am assisted by the rights of access, rectification, cancellation, and opposition that I can exercise by request to the responsible researcher at the contact address that appears in this document. These data may not be transferred without my express consent and I do not grant it in this act.

I declare that I have read and know the content of this document, understand the commitments that I assume and expressly accept them. And, therefore, I sign this informed consent voluntarily to express my desire to participate in this RESEARCH study until I decide otherwise. By signing this consent, I do not waive any of my rights. I will receive a copy of this consent to keep for future reference.

Name of the woman　　　　Name of the researcher

ID and Signature　　　　ID i Signature
