# Supplementary material for: Effectiveness of birth plan counselling based on shared decision making: A cluster randomized controlled trial (APLANT)
Source: PLoS One. 2022 Sep 12;17(9):e0274240. doi: 10.1371/journal.pone.0274240 (PMC9467369; doi:10.1371/journal.pone.0274240)
Supplement: Attachment — Submitted filename: Response to Reviewers.docx [file pone.0274240.s006.docx]

**Response to reviewers**

PONE-D-21-03373

Effectiveness of birth plan counselling based on share decision making: a cluster randomized controlled trial (APLANT)

PLOS ONE
Dear Dr. López-Gimeno,
Thank you for submitting your manuscript to PLOS ONE. After careful consideration, we feel that it has merit but does not fully meet PLOS ONE’s publication criteria as it currently stands. Therefore, we invite you to submit a revised version of the manuscript that addresses the points raised during the review process.

The manuscript has been evaluated by three reviewers, and their comments are available below.

The reviewers have raised some minor concerns about the manuscript. Specifically, they feel the manuscript requires additional work in clarifying the theoretical basis for the study, particularly noting the need for greater attention to the conceptual definitions in the Introduction. In addition, they request further detail in the methodology and statistical analyses. Language editing is also recommended for this submission.

Could you please carefully revise the manuscript to address all comments raised?

Please submit your revised manuscript by **Nov 25 2021** 11:59PM. If you will need more time than this to complete your revisions, please reply to this message or contact the journal office at [plosone@plos.org](about:blank). When you're ready to submit your revision, log on to https://www.editorialmanager.com/pone/ and select the 'Submissions Needing Revision' folder to locate your manuscript file.
Please include the following items when submitting your revised manuscript:

- A rebuttal letter that responds to each point raised by the academic editor and reviewer(s). You should upload this letter as a separate file labeled 'Response to Reviewers'.
- A marked-up copy of your manuscript that highlights changes made to the original version. You should upload this as a separate file labeled 'Revised Manuscript with Track Changes'.
- An unmarked version of your revised paper without tracked changes. You should upload this as a separate file labeled 'Manuscript'.

If you would like to make changes to your financial disclosure, please include your updated statement in your cover letter. Guidelines for resubmitting your figure files are available below the reviewer comments at the end of this letter.
If applicable, we recommend that you deposit your laboratory protocols in protocols.io to enhance the reproducibility of your results. Protocols.io assigns your protocol its own identifier (DOI) so that it can be cited independently in the future. For instructions see: [https://journals.plos.org/plosone/s/submission-guidelines#loc-laboratory-protocols](about:blank#loc-laboratory-protocols). Additionally, PLOS ONE offers an option for publishing peer-reviewed Lab Protocol articles, which describe protocols hosted on protocols.io. Read more information on sharing protocols at [https://plos.org/protocols?utm_medium=editorial-email&utm_source=authorletters&utm_campaign=protocols](about:blank).
We look forward to receiving your revised manuscript.
Kind regards,
Avanti Dey, PhD
Staff Editor
PLOS ONE
Journal Requirements:

**Reviewers' comments:**
Reviewer's Responses to Questions

**Comments to the Author**
1. Is the manuscript technically sound, and do the data support the conclusions?
The manuscript must describe a technically sound piece of scientific research with data that supports the conclusions. Experiments must have been conducted rigorously, with appropriate controls, replication, and sample sizes. The conclusions must be drawn appropriately based on the data presented.

Reviewer #1: Partly

Reviewer #2: Yes

Reviewer #3: Yes

2. Has the statistical analysis been performed appropriately and rigorously?

Reviewer #1: Yes

Reviewer #2: Yes

Reviewer #3: Yes

3. Have the authors made all data underlying the findings in their manuscript fully available?
The [PLOS Data policy](about:blank#sharing) requires authors to make all data underlying the findings described in their manuscript fully available without restriction, with rare exception (please refer to the Data Availability Statement in the manuscript PDF file). The data should be provided as part of the manuscript or its supporting information or deposited to a public repository. For example, in addition to summary statistics, the data points behind means, medians and variance measures should be available. If there are restrictions on publicly sharing data—e.g. participant privacy or use of data from a third party—those must be specified.

Reviewer #1: Yes

Reviewer #2: Yes

Reviewer #3: Yes

4. Is the manuscript presented in an intelligible fashion and written in standard English?

PLOS ONE does not copyedit accepted manuscripts, so the language in submitted articles must be clear, correct, and unambiguous. Any typographical or grammatical errors should be corrected at revision, so please note any specific errors here.

Reviewer #1: Yes

Reviewer #2: Yes

Reviewer #3: Yes

5. Review Comments to the Author

Please use the space provided to explain your answers to the questions above. You may also include additional comments for the author, including concerns about dual publication, research ethics, or publication ethics. (Please upload your review as an attachment if it exceeds 20,000 characters)

**REBUTAL LETTER**

Dear Dr Avanti Dey, and reviewers

Thank you for the expert review of our manuscript entitled “Effectiveness of birth plan counselling based on share decision making: a cluster randomized controlled trial (APLANT)” PONE-D-21-03373.

We want to acknowledge to you and to the reviewers the positive and constructive comments on our paper. We have used the reviewers’ comments to improve our paper further and we are pleased to send you hereby our revised manuscript.

The reviewers’ comments and our response after each comment are provided below. The responses are highlighted in bold text. The actual changes in the manuscript are provided in bold text and yellow colour.

Please find below the responses to each of the comments and suggestions.

**Editor comments**

When submitting your revision, we need you to address these additional requirements.

1. Please ensure that your manuscript meets PLOS ONE's style requirements, including those for file naming. The PLOS ONE style templates can be found at 
https://journals.plos.org/plosone/s/file?id=wjVg/PLOSOne_formatting_sample_main_body.pdf and 
[https://journals.plos.org/plosone/s/file?id=ba62/PLOSOne_formatting_sample_title_authors_affiliations.pdf](about:blank)

***Response 1***

*We reviewed and formatted the manuscript according the PLOS ONE requirements.*

2. Please review your reference list to ensure that it is complete and correct. If you have cited papers that have been retracted, please include the rationale for doing so in the manuscript text or remove these references and replace them with relevant current references. Any changes to the reference list should be mentioned in the rebuttal letter that accompanies your revised manuscript. If you need to cite a retracted article, indicate the article’s retracted status in the References list and also include a citation and full reference for the retraction notice.

***Response 2***

*We have reviewed the references list and we have added the PMID or ISSN.*

*We have replaced the reference number 33 with a relevant current one.*

*Due to a suggestion of the reviewer 3, there have been changes in the discussion. This has implied eliminating the bibliographic reference 41 and 42 and a new reference with the number 41 has been added.*

3. While we appreciate that you have included a patient flow diagram in Figure 1, we request that you please use the CONSORT flow diagram to illustrate your study, available at [http://www.consort-statement.org/consort-statement/flow-diagram](about:blank)

***Response 3***

*Thanks for your proposal, we have described the flow diagram according to the Consort cluster randomised trial statement. We have changed the figure 2 file.*

4. Thank you for submitting your clinical trial to PLOS ONE and for providing the name of the registry and the registration number. The information in the registry entry suggests that your trial was registered after patient recruitment began. PLOS ONE strongly encourages authors to register all trials before recruiting the first participant in a study.

As per the journal’s editorial policy, please include in the Methods section of your paper:
a) your reasons for your delay in registering this study (after enrolment of participants started);
b) confirmation that all related trials are registered by stating: “The authors confirm that all ongoing and related trials for this drug/intervention are registered

***Response 4***

*You are right, so we have clarified this point in the methods section.*

*Page 7. Line 142-148.*

**The study was approved by the Ethical Committee of Clinical Investigation of the University Institute for Research in Primary Care, Barcelona in December 2016.**

**The study period lasted from 1 November 2017 to 8 July 2019.**

**Our clinical trial was registered in Clinical Trial.gov (NCT03744416) in November 2018. This happened because in that period we were not aware that this was the general practice for this type of intervention studies. The authors confirm that all ongoing and related trials for this intervention are registered.**

5. Thank you for stating in your Funding Statement: "The trial was funded partially by the “Nurse and Society Foundation” as part of the Nurse Research Projects Grants (PR-224/2017), Barcelona (Spain): EL, GS, MV, LB, GV, GF
https://www.infermeriaisocietat.cat/
As well by the “Grant XB. Barcelona, Research modules, 10th Edition”, Institut Català de la Salut”, Spain 2018: EL, GV <https://atencioprimariaicsbcn.files.wordpress.com/2019/05/jornada_de_recerca_resultats_ajuts_xb_2019.pdf>

The funders had no role in study design, data collection and analysis, decision to publish, or preparation of the manuscript."

Please provide an amended statement that declares *all* the funding or sources of support (whether external or internal to your organization) received during this study, as detailed online in our guide for authors at http://journals.plos.org/plosone/s/submit-now.  Please also include the statement “There was no additional external funding received for this study.” in your updated Funding Statement. 
Please include your amended Funding Statement within your cover letter. We will change the online submission form on your behalf.

***Response 5***

*We have added information concerning to the funders in the funding statement and in the cover letter.*

6. Thank you for stating the following financial disclosure: "The trial was funded partially by the “Nurse and Society Foundation” as part of the Nurse Research Projects Grants (PR-224/2017), Barcelona (Spain): EL, GS, MV, LB, GV, GF
https://www.infermeriaisocietat.cat/
As well by the “Grant XB. Barcelona, Research modules, 10th Edition”, Institut Català de la Salut”, Spain 2018: EL, GV [https://atencioprimariaicsbcn.files.wordpress.com/2019/05/jornada_de_recerca_resultats_ajuts_xb_2019.pdf](about:blank)

The funders had no role in study design, data collection and analysis, decision to publish, or preparation of the manuscript."

We note that one or more of the authors is affiliated with the funding organization, indicating the funder may have had some role in the design, data collection, analysis, or preparation of your manuscript for publication; in other words, the funder played an indirect role through the participation of the co-authors. If the funding organization did not play a role in the study design, data collection and analysis, decision to publish, or preparation of the manuscript and only provided financial support in the form of authors' salaries and/or research materials, please do the following:

a. Review your statements relating to the author contributions and ensure you have specifically and accurately indicated the role(s) that these authors had in your study. These amendments should be made in the online form.

b. Confirm in your cover letter that you agree with the following statement, and we will change the online submission form on your behalf: 
“The funder provided support in the form of salaries for authors [insert relevant initials] but did not have any additional role in the study design, data collection and analysis, decision to publish, or preparation of the manuscript. The specific roles of these authors are articulated in the ‘author contributions’ section.

[Note: HTML markup is below. Please do not edit.]
***Response 6***

*We also included this sentence in the cover letter.*

**Reviewer #1**:

A two-arm controlled randomized cluster designed trial was conducted to compare Mackey Satisfaction with Childbirth Rating Scale between intervention of shared decision making to standard midwife counselling. Satisfaction ratings of the birth plan were similar between the two groups.
Minor revisions:
**Comment 1.**

1-Lines 217, 262, 280, 308 & 315: Correct typographical error: “Fisher’s exact test.”

**Response 1.**

*Thanks for outlining these typographical errors and we amended all these.*

**Comment 2**
2- Line 219: Specify the statistical methods used for the multivariate analysis.

***Response 2.***

*Thanks for your indication. We revised the statistical analysis section, and we wrote a more accurate text, as well as the statistical methods used for the multivariate analysis.*

*Page 12 & 13. Line 250-276.*

**The data collected were entered to the SPSS Version 24 statistical programme and the data were only accessible to the investigative team. Descriptive analysis of all the variables was performed. For the childbirth satisfaction outcome women with caesarean section were excluded, because the scale was validated in Spanish for women with vaginal birth.**

**Bivariate analysis was performed between the intervention group and birth plan presentation to the hospital, childbirth satisfaction and obstetrical results. The analyses were performed per protocol basis, because we analysed only women who received intervention and followed in the study, according to the treatment group allocated at randomisation.**

**In addition, we analysed whether the presentation of the BP to the hospital was related to the obstetrics outcomes and the grade of satisfaction of women.**

**For comparison of categorical variables, the non-parametric test of Fisher’s exact test was used. To compare quantitative variables, parametric Student’s test or ANOVA test were used and non-parametric test Mann-Whitney U. A p value <0.05 was considered statistically significant.**

**To evaluate the effect of the intervention counselling with the BP presentation to the hospital multinomial logistic regression analysis model was performed, adjusted to the confounder demographic variables related to the intervention group.**

**To determine which obstetric variables (type of birth, epidural, methods of pain relief, breastfeeding in delivery room, early skin-to-skin contact, maternal and neonatal complications) could have a joint relation with presenting the birth plan, we carried out another logistic regression model. The model was adjusted by variables clinically relevant or who had a level of significance <0.05 in the bivariate analysis (belonging to the control group or intervention group, age, country of origin, education, previous births,). Finally, the same model was made, but making an automatic selection of variables (stepwise), to identify which variables had a statistically significant relation with the fact of presenting the birth plan. Adjusted odd ratio (OR) and the 95% CI were determined.**

**Comment 3**

3- Indicate the date range subjects were enrolled in the study.

***Response 3*** *The date range subjects were enrolled in the study was from 1 November 2017 to 8 July 8 2019 as its specified in line 144.*

**Comment 4**

4- State and justify the study’s target sample size with a pre-study statistical power calculation. The power calculation should include sample size, alpha level (indicating one or two-sided), minimal detectable difference and statistical testing method.

***Response 4***

*We included additional information about the sample size calculation*

*Page 8. Line 162-168.*

**Sample size calculation was based on the variable “presentation of the BP to the hospital” to detect a minimal difference of 20% between two groups, according to a previous descriptive pilot study (n=211 women) with a prevalence of BP presentation of 48%. A higher prevalence of around 68% was estimated in the intervention group (IG). α risk of 0.05 and β risk of 0.2 were accepted in the bilateral contrast. It was calculated that 133 women were needed in the control group (CG) and 133 in the intervention group (IG). A loss to follow-up of 15% was estimated.** The calculation was made using the macros of the SPSS Version 24 and the clusters design was contemplated.

**Comment 5**
5- Table 4: Clarify if the ANOVA method or logistic regression was used for the analysis.

***Response 5***

*Page 18 & 19. Line 353, 355-361.*

*We performed a bivariate analysis, between demographic and clinical factors and its relation to the hospital birth plan presentation. So, we used the ANOVA test to compare quantitative variables and the Fisher’s exact test to compare categorical variables. We deleted the Odds ratio in table 4*

*We have subsequently performed a logistic regression analysis to evaluate the effect of the intervention counselling with the presentation of the birth plan to the hospital, adjusted to the confounder demographic variables related to the intervention group. (Table 5).*

**Comment 6**
6- Clarify that results in Table 5 are derived from univariate models. Specify the statistical methods used to generate the results.

***Response 6***

*Page19-20. Line 372.*

*Now table 6. We performed a bivariate analysis, between presenting the birth plan to the hospital (yes/no) and its relation to the obstetric results and childbirth satisfaction. So, we used the Fisher’s exact test to compare categorical variables and the t Student’s test to compare quantitative variables. We also calculated the Odds ratio.*

**Comment 7**

7- Table 6: Specify the criteria for determining which variables were retained in the multivariate model.

***Response 7***

*Thanks for the comment, we have realised that some additional information was needed in the statistical methods. So, we amended the text, presented above in response 2. Now is table 7 (line 382).*

**Comment 8**
8- Indicate if any adverse events occurred during the course of the study.

***Response 8***

*Thanks for your indication. In the data collection section, we have specified that no adverse events occurred during the study.*

*Page 11. Line 248.*

**There were no adverse effects during the study.**

**Reviewer #2:**

Very well-designed research, with findings that correspond to the objectives of the study, especially with regard to the use of humanized care methodologies. Experimental research very well outlined in the method section. Very little possibility that there was no differentiation between the control and experimental groups, which validates the results.

***Response***

*Thank you for your assessment and dedication in reviewing our study.*

**Reviewer #3:**

Review Plos One-D-21-03373
Effectiveness of birth plan counselling based on share decision making: a cluster randomized controlled trial (APLANT)
Overall
This is an interesting article, which presents the findings of RCT on a relevant topic. More insight into the use of birth plans and SDM has the potential to improve women’s childbirth experiences, but limited research has been done. This study helps to fill this gap.
Overall, the research is sound. However, on certain points it needs more clarity and further explaining. This can partly be achieved by improving the language. The English used in this article needs improvement: certain choice of words is unclear; sentences are unfinished, and it is not always clear to what ‘they’ or ‘this’ is referring to. I will give some examples in my detailed feedback, but they are not all inclusive.
The article needs to be clearer on the analyses that have been done, especially with regard to intention-to-treat and per protocol analyses.

**Detailed feedback
Abstract**

**Comment 1**
A. The background needs more clarity.

B. What is exactly the aim of the study?
C. In the methods, it needs to be clear where the two approaches differ. That the CG also received the BP and counselling, but the counselling was not based on SDM and there was no leaflet.

D. I would state that the MSCRS was used to measure the primary outcome.

**Responses 1**

*We appreciate your suggestions, so we have made amendments in the text.*

*1****.* A. The background needs more clarity.**

*1.A Background.*

Page 3 Line 46.

**The birth plan (BP) is a written document in which the pregnant woman explains her wishes and expectations about childbirth to the health professionals and aims to facilitate the pregnant woman's decision-making. Midwives' support to women during the development of the BP is essential, but** it's unknown if shared decision making (SDM) is effective on birth plan counselling.

1.B What is exactly the aim of the study?

*Page 3. Line 51.*

**We hypothesized** **that women who received** counselling based on SDM during pregnancy more frequently present the BP to the hospital, are more satisfied with the childbirth experience, and have better obstetrics outcomes **than women who received standard counselling.** We also aimed to identify if **women who presented** BP to the hospital have better obstetric outcomes and **are more satisfied** with the childbirth experience.

1.C. In the methods, it needs to be clear where the two approaches differ. That the CG also received the BP and counselling, but the counselling was not based on SDM and there was no leaflet.

*Page 3. Line 59&60.*

The control group (CG) **only received standard counselling by midwives in relation to the BP**.

1.D. I would state that the MSCRS was used to measure the primary outcome.

*Page 3. Line 60-63.*

**The primary outcomes were birth plan presentation to the hospital, obstetrics outcomes and satisfaction with childbirth experience.** Mackey Childbirth Satisfaction Rating Scale (MCSRS) **was used to measure the childbirth satisfaction**.

**Comment 2**
I suggest replacing Findings with Results. In the article itself you also use the word Results, which is more appropriate for an RCT.

**Response 2**

*Thank you for your valuable contributions. We made the change*

*Page 4. Line 64.*

**Results**

**Comment 3**
‘That’ should be added in the sentence “The results showed that women of the IG …”
Add ‘in the IG’ to the ‘sentence “…, the satisfaction with childbirth experience was high in the IG and similar.

**Response 3**

*We have rectified the text.*

*Pag 4. Line 66&68.*

**That** the results showed women of the IG presented the BP less frequently to the hospital compared to CG (57.8% vs 75.1%; p <0.001). The satisfaction with childbirth experience was high **in the IG** and similar to the CG (150.2 vs 153.4; p=0.224)

**Comment 4**

I have difficulty with the firm statement: Women who presented BP had a greater probability of spontaneous vaginal birth aOR=2.07 (95% CI: 1.23-3.5) and early skin-to-skin contact aOR=2.29 (95% CI: 1.2-4.2). This is a per protocol analysis, where it is not known if groups are comparable. There is no correction for possible confounders, such as parity.

**Response 4**

*Thanks, for your contribution, we realised that some additional information is needed in the statistical methods.*

*In relation to the type of analysis, you are right, this is an analysis per protocol. So, we corrected the statistical analysis section. So, we performed again, the multinomial analysis, including the possible confounders, demographical and obstetric ones. We also, have rewritten the statistical analysis section. Now, the results are different.*

*Page4. Line 74-76.*

Women who presented BP had a greater probability of **using combined non-pharmacological and pharmacological methods for pain relief aOR=2.06 (95% CI: 1.30-4.30) and early skin-to-skin contact aOR=2.08 (95% CI: 1.07-4.04).**

Statistical analysis

*Page 12&13 Line 250-276.*

**The data collected were entered to the SPSS Version 24 statistical programme and the data were only accessible to the investigative team. Descriptive analysis of all the variables was performed. For the childbirth satisfaction outcome women with caesarean section were excluded, because the scale was validated in Spanish for women with vaginal birth.**

**Bivariate analysis was performed between the intervention group and birth plan presentation to the hospital, childbirth satisfaction and obstetrical results. The analyses were performed per protocol basis, because we analysed only women who received intervention and followed in the study, according to the treatment group allocated at randomisation.**

**In addition, we analysed whether the presentation of the BP to the hospital was related to the obstetrics outcomes and the grade of satisfaction of women.**

**For comparison of categorical variables, the non-parametric test of Fisher’s exact test was used. To compare quantitative variables, parametric Student’s test or ANOVA test were used and non-parametric test Mann-Whitney U. A p value <0.05 was considered statistically significant.**

**To evaluate the effect of the intervention counselling with the BP presentation to the hospital multinomial logistic regression analysis model was performed, adjusted to the confounder demographic variables related to the intervention group.**

**To determine which obstetric variables (type of birth, epidural, methods of pain relief, breastfeeding in delivery room, early skin-to-skin contact, maternal and neonatal complications) could have a joint relation with presenting the birth plan, we carried out another logistic regression model. The model was adjusted by variables clinically relevant or who had a level of significance <0.05 in the bivariate analysis (belonging to the control group or intervention group, age, country of origin, education, previous births,). Finally, the same model was made, but making an automatic selection of variables (stepwise), to identify which variables had a statistically significant relation with the fact of presenting the birth plan. Adjusted odd ratio (OR) and the 95% CI were determined.**

**Comment 5**

In the conclusion, pharmacological and non-pharmacological methods for pain relief are mentioned, but not the difference in epidural. Throughout the article, there is an inconsistency in presenting these variables. Sometimes only epidural is mentioned, sometimes only the combination of pharmaceutical and non-pharmaceutical. And occasionally both.
**Response 5**
*We appreciate your suggestion, and you are right, so we modified the text.*

*Page 4. Line 78-83.*

This counselling intervention was not effective to increase the presentation of the BP to the hospital and women’s satisfaction with childbirth **however, it was related to a lower usage of analgesia epidural, a** higher combination of pharmacological and non-pharmacological methods for pain relief and the initiation of breastfeeding in the delivery room. Presenting the BP to the hospital increased the likelihood **of using pharmacological and non-pharmacological methods for pain relief** and early skin-to-skin contact.

**Introduction**

**Comment 6**
Line 77: I would replace ‘aspects’ with the word ‘choices’.

**Response 6**

*Thank you again. We have made the change***.**

*Page 5. Line 86.*

related **choices** and promotes participation and improvement

**Comment 7**
Introduce abbreviation like SDM and BP by writing them in full when using them for the first time in the article.

**Response 7**

*You're right, it wasn't specified in the manuscript. We have introduced the meaning of the abbreviations into the text.*

*Page 5. Line 86 & 91.*

**of share decision making** (SDM) through a relationship of collaboration, information, and adequate advice [1].

One of the objectives of the **birth plan** (BP) is to facilitate..

**Comment 8**
Line 78-81: I suggest replacing the women (=line 79) with a woman as midwife is also single.

**Response: 8**

*We rectify the grammatical mistake.*

*Page 5. Line 88.*

relationship between the wom**a**n and the midwife

**Comment 9**
Line 83: replace ‘with’ with ‘in’.

**Response 9**

*Done.*

*Page 5. Line 92.*

The BP is a written document **in** which a woman states her wishes..

**Comment 10**
Line 84: replace ‘of’ with ‘about’.

**Response 10**

*Thanks for improving the language of the text.*

*Page 5. Line 93.*

the professionals who accompany her during childbirth **about** her preferences ..

**Comment 11**
Line 88: what is meant by ‘… for focusing discussion …”?

**Response 11**

We have changed by the word **dialogue**

*Page 5. Line 97.*

The professionals must adopt strategies for focusing **dialogue** and thereby ..

**Comment 12**

Line 89: it is Afshar et al.

**Response 12**

*We add et al.*

*Page 5. Line 98.*

Afshar **et al. [**12] proposed that professionals should use the Epstein

**Comment 13**

Line 90: Epstein model. There are many models for SDM, please explain this model a bit further, e.g. in a Box.

**Response 13**

*We have included additional information in the paragraph.*

*Page 5. Line 100-104.*

**Epstein el al, propose five steps to communicate the evidence for sharing decision making with patients and that includes: understanding the patient’s (and family member’s) experience and expectations, building partnership, providing evidence, (including a balanced discussion of uncertainties), presenting recommendations and finally, checking for understanding and agreement.**

**Comment 14**
Line 93: ‘… to the person …’, what person is meant?

**Response 14**

*The bibliography consulted on SDM refers mainly to “patient with a disease process”. We have thought it is more appropriate to write "person" as a broader concept since SDM can occur in other situations that are not exclusive disease processes.*

*Pregnancy is a physiological process that most of the time there is no deviation from the normality of the pregnant woman and is not considered a sick person since the patient. However, we changed “person” by “patient.”*

*Page 5. Line 105 & 106.*

SDM is characterised by the relationship of collaboration between the **patient** and the professional for deciding together what option adapts better to the **patient** considering..

**Comment 15**
Line 101: Here you refer to reference 16 and 17, in the discussion to 20 and 21. Please make this

consistent.

**Response 15**

*Thank you. We have clarified the sentence in the introduction.*

*Page 6. Line 114.*

Since then, the percentage of women presenting the BP to the hospital, **in Spain**, is variable and not well-known, ranging from 2.8% to 69% according to the studies

*We amended as well in the discussion section.*

*Page 21. Line 393 & 394.*

The BP was presented to the hospital by 65.9% of the women, being a higher value than that of other studies reporting 34.7% **in Netherland and 48.8% in USA [20,21], but being lower than a study conducted in Catalonia in 2015 [17].**

**Comment 16**
Line 103-105: Please, elaborate here what this means for the BP. And how women present their BP they go to the hospital when they are in labor.

**Response 16**

*Thank you for your valuable input. We have added information to make the text clearer.*

*Page 6. Line 116-119.*

**To enable women to express their choices about childbirth** midwives carry out the BP counselling during the prenatal care, in the third trimester of pregnancy, in primary health care. The birth is attended in the reference hospital. When **women are in labour, they give their BP to the hospital midwives.**

**Comment 17**

Line 107: words are missing after ‘… evaluates the …’.

**Response 17**

*Thank you again. We have rephrased the sentence to make it easier to understand*

*Page 6. Line 122-124.*

In addition, the **Department of Health** of Catalonia region (Spain) periodically evaluates the opinion of women about childbirth. **This department also assesses the degree of women's satisfaction with the information received from professionals** about pregnancy and childbirth. At the last inquiry the 67% of women considered that they received sufficient information related to their pregnancy and childbirth [18].

**Comment 18**
Line 112-114: make clearer that this accounts for the group who have actually presented the BP (with or without the intervention). This nuance is hard to capture when you are not familiar with the study.

**Response 18**

*We have modified these sentences.*

*Page 6. Line 129-131.*

We hypothesized that women who received counselling based on SDM during pregnancy more frequently present the BP to the hospital, are more satisfied with the childbirth experience, and have better obstetrics outcomes **than women who received standard counselling.** We also aimed to identify if **women who presented** BP to the hospital have better obstetric outcomes and **are more satisfied** with the childbirth experience.

**Comment 19**

Line 115: replace ‘make up’ with ‘are’.

**Response 19**

*We have made the proposed change
Page 6. Line 132.*

The results of this study **are** part of a larger study which also evaluated if the counselling intervention changes the preferences of the women in the BP.

Methods

**Comment 20**
Line 121-122: Contamination is a good point. Is it also very unlikely that women of the different groups are in touch with each other?

**Response 20**

*Thank you for your fine reflection. We modified the text.*

*Page 7. Line 140 & 141.*

**ASSIR health centres are placed in different cities and each one has a different referral hospital, thus minimising contact between women as well as between midwives.**

**Comment 21**
Line 133: how were the midwives of the CG group informed about the situation, were they aware that midwives in the IG received instruction around SDM?

**Response 21**

*We added some information.*

*Page 9. Line 201-203.*

**Furthermore, they were not informed about the training activity that midwives in the intervention group received, nor about the existence of the leaflet.**

**Comment 22**
Line 195-196: Who filled out the data collection form? This should be indicated.

**Response 22**

*Thanks, we added the information.*

*Page 10. Line 225.*

During recruitment of the study participants, demographic and obstetric data were collected**, by midwives**, in a data collection form, ....

**Comment 23**
Line 203: How did the women report these data and when? There should be information about this in the text.

**Response 23**

*Thank you for your contribution. We have added the explanatory sentences*

*Page 11. Line 228- 234.*

**In the postpartum check, between four and six weeks,** midwives of the Primary Care Units collected information about pregnancy (level of obstetric risk, antenatal maternal education, use of internet for the BP and childbirth) and childbirth (onset of labour, type of birth, episiotomy, methods of pain relief, early skin-to-skin contact, initiation of breastfeeding in the birthing room, maternal and neonatal complications). **At the same postpartum check, midwives provided a questionnaire to the woman to look for data about whether she had received** sufficient information about childbirth during pregnancy...

*Page 11. Line 246.*

**Woman answered the questionnaire and the MCSRS in the ASSIR’s waiting room and then handed it to the midwife.**

**Comment 24**

Line 212: add to the end of this sentence ‘…with higher scores indicating higher satisfaction’.

**Response 24**

*Thank you, Done.*

*Page11. Line 242.*

The overall satisfaction is the sum of all the subscales, with a maximum score of 175 points, **with higher scores indicating higher satisfaction**

**Comment 25**
Line 213: In this section, there should also be information about the type of analysis in this RCT: was it intention-to-treat? Apparently, there was also a per protocol analysis. This information needs to be in this section as well.

**Response 25**

*Page 14. Line 303.*

*Thank you for the advice. The analyses were performed per protocol basis, thus we analysed only women who received intervention and followed in the study (n=416), according to the treatment group allocated at randomisation. We have changed the table 1, and now we presented the baseline characteristics of the analysed participants (n=416). The differences found between groups are similar as the 482 enrolled women. We include below the removed table 1, (n=482) enrolled participants.*

**Table 1. Baseline characteristics of the participants (n=482)**

|  | **Total** | **Control Group** | **Intervention Group** | ***p*** |
| --- | --- | --- | --- | --- |
|  | N=482 | n=221 (45.9) | n=261 (54.1) |  |
|  | n (%) | n (%) | n (%) |  |
| **Age, mean (SD)** | 31.9 (5.5) | 31.2 (5.2) | 32.5 (5.7) | 0.008^1^ |
| **Country of origin** |  |  |  |  |
| Spain | 346 (71.8) | 175 (79.2) | 171 (65.5) | 0.001^2^ |
| Other | 136 (28.2) | 46 (20.8) | 90 (34.5) |  |
| **Education** |  |  |  |  |
| Primary school or less | 89 (18.5) | 52 (23.5) | 37 (14.2) | <0.001^2^ |
| High school | 204 (42.3) | 102 (46.2) | 102 (39.1) |  |
| University | 189 (39.2) | 67 (30.3) | 122 (46.7) |  |
| **Employment** |  |  |  |  |
| No | 109 (22.6) | 49 (22.2) | 60 (23) |  |
| Yes | 373 (77.4) | 172 (77.8) | 201 (77) | 0.831^2^ |
| **Partner** |  |  |  |  |
| No | 25 (5.2) | 11 (5) | 14 (5.4) |  |
| Yes | 457 (94.8) | 210 (95) | 247 (94.6) | 0.849^2^ |
| **Previous birth(s)** |  |  |  |  |
| No | 267 (55.4) | 116 (52.5) | 151 (57.9) |  |
| Yes | 215 (44.6) | 105 (47.5) | 110 (42.1) | 0.238^2^ |
| **Previous birth plan** | n= 212* | n=103 (48.6) | n=109 (51.4) |  |
| No | 117 (55.2) | 57 (55.3) | 60 (55) |  |
| Yes | 95 (44.8) | 46 (44.7) | 49 (45) | 0.966^2^ |

Data are expressed as n (%) 1= Student’s t test; 2= **Fisher’s exact test**

*women with previous births: 3 missing values

*We included the statistical analysis’s text in response 4.*

*Page 18. Line 360.*

*We also included a new table with the results of the multinomial logistic regression model of demographic factors related to the birth plan presentation and intervention group (table 5).*

**Table 5. Analysis of demographic factors related to the birth plan presentation and intervention group. Multinomial logistic regression model.**

| **Birth plan presentation to the hospital** | ***aOR (*95% CI)** | ***p*** |
| --- | --- | --- |
| **Intervention Group** |  |  |
| **Control** | **Ref.** | **<0.001** |
| **Intervention** | **0.45 (0.29-0.70)** |  |
| **Age Mean (Sd)** | **0.98(0.94-1.02)** | **0.286** |
| **Country of origin** |  |  |
| **Spain** | **Ref.** | **0.495** |
| **Other** | **0.85(0.53-1.36)** |  |
| **Education** |  |  |
| **Primary school or less** | **Ref.** | **0.351** |
| **High school** | **0.82(0.46-1.46)** |  |
| **University** | **1.16(0.62-2.19)** |  |

**aOR: adjusted odds ratio; 95 CI%: 95% confidence interval**

**Comment 26**
Line 2014: ‘introduced’ is not the right word

**Response 26**

*Done*

*Page 12. Line 250.*

The data collected were **entered** to the SPSS Version 24.

**Comment 27**
Line 222: how was level of obstetric risk defined?

**Response 27**

*The level of obstetric risk is based on the current protocol of Catalonia. However, for the statistical analysis obstetric risk level was grouped into “low” for low and medium and “high” for high and very high”*
*So, we have specified these levels on table 4.*

*Page 18. Line 353.*

**Results
Comment 28**

Line 251 and 252. Apparently, CS were excluded. This should be motivated in the methods section.

**Response 28**

*Thanks, we included additional information in statistical analysis.*

*Page 12. Line 252.*

**For the childbirth satisfaction outcome women with caesarean section were excluded, because the scale was validated in Spanish for women with vaginal birth.**

**Comment 29**

Line 258: replace ‘greater’ with ‘higher’.

**Response 29**

*Done*

Page 14. Line 300.

However, in the IG the age of the women was **higher (mean=32.4 vs. 31.2; p< 0.028)**

**Comment 30**

Line 259: elaborate from which countries these women came, e.g. from high income or middle/low income countries?

**Response 30**

*We did not collect the information regarding the country of origin, so we only had information about if women were native or foreign.*

**Comment 31**

Table 1: why no information on parity?

**Response 31**

*We collected the information regarding the number of previous births, subsequently we grouped in a new categorical variable previous birth (yes/no). We include below a table with the quantitative variable data, and we analysed if the groups were comparable. We used the u Mann Whitney test, and we did not find any significative difference.*

|  | | **Group** | | | | | | | | | | | **Total** | | | | | | |
| --- | --- | --- | --- | --- | --- | --- | --- | --- | --- | --- | --- | --- | --- | --- | --- | --- | --- | --- | --- |
|  |  | **Control** | | | | | | **Intervention** | | | | |  |  |  |  |  |  |  |
|  |  | **N** | | | **%** | | | **N** | | **%** | | | **N** | | | **%** | | | |
| **N previous births** | | 103 | | | 53.37 | | | 133 | | 59.64 | | | 236 | | | 56.73 | | | |
| 0 | |  |  |  |  |  |  |  |  |  |  |  |  |  |  |  |  |  |  |
| 1 | | 71 | | | 36.79 | | | 75 | | 33.63 | | | 146 | | | 35.10 | | | |
| 2 | | 14 | | | 7.25 | | | 14 | | 6.28 | | | 28 | | | 6.73 | | | |
| 3 | | 3 | | | 1.55 | | | 1 | | 0.45 | | | 4 | | | 0.96 | | | |
| 4 | | 2 | | | 1.04 | | | . | | 0.00 | | | 2 | | | 0.48 | | | |
| Total | | 193 | | | 100.00 | | | 223 | | 100.00 | | | 416 | | | 100.00 | | | |
|  | | | | **N** | | **Media** | **Sd** | | **IC95-Low** | | **IC95-Up** | | | **Min.** | **Median** | | | **Max.** | **IQR** |
| **N previous births** | Control | | | 193 | | 0.60 | 0.78 | | 0.49 | | 0.71 | | | 0.00 | 0.00 | | | 4.00 | 1.00 |
|  | Intervention | | | 223 | | 0.48 | 0.64 | | 0.39 | | 0.56 | | | 0.00 | 0.00 | | | 3.00 | 1.00 |
|  | Total | | | 416 | | 0.53 | 0.71 | | 0.47 | | 0.60 | | | 0.00 | 0.00 | | | 4.00 | 1.00 |
| **Variable** | | | **Test** | | | | | | | | | **P Value sign** | | | | | | | |
| **N previous births** | | | **U Mann-Whitney** | | | | | | | | | **0.147** | | | | | - | | |

**Comment 32**

Table 2: Participation in decision making in first and second stage. How was this measured? What do the given numbers mean? There is no information in the method section.

**Response 32**

*Thanks, you are right, so we included additional information in data collection*

*Page 11. Line 243-245.*

**The MCSRS also includes two questions about women’s satisfaction with the participation in the decision making in relation to the first and second stage of birth, using the same 5 points Likert scale.**

**Comment 33**
Line 287: ‘… differences were not statistically significant.’ And how clinical relevant is a difference of 3 points on a scale that scores up to 175? Emphasizing the difference is not relevant.

**Response 33**
*Thanks, we appreciate your indication, so we changed this text.*

*Page 16. Line 326 & 327.*

The mean global satisfaction **was high in the control group and similar to the intervention group (153.4 vs 150.2; p=0.224).**

**Comment 34**

Line 304-306: what do we know of women's intention to breastfeed? Does this perhaps explain the difference?

**Response 34**

*Thanks for your contribution, but still we cannot publish the results of the women preferences expressed in the birth plan. Because your question, we checked the data in the thesis manuscript and the women intention to breastfeed in delivery room, after receiving the counselling intervention, is similar in both groups: CG (n=133;70.7%;) and IG (n=168;74%; p=0.508).*

**Comment 35**

Table 4: why was parity not included as a possible confounder?

**Response 35**

*Thanks, for your contribution.*

*We have included the previous births (yes/no) as a possible confounder in the multinomial logistic regression (table 7). We have carried out the statistical analysis again since there were some mistakes.*

*Page 20. Line 375- 383.*

**Finally, the logistic analysis model adjusted for confounder and clinical variables showed that women in IG had a lower likelihood of presenting the BP compared to women in the CG (aOR=0.37; 95%CI: 0.24 to 0.58). Moreover, the women who presented the BP had double the probability to use non-pharmacological and pharmacological methods for pain relief (aOR=2.06; 95% CI:1.30 to 4.30), and to have early skin-to-skin contact (aOR =2.08; 95% CI: 1.07 to 4.04) with respect to those who did not present the BP (Table 7).**

*We also attach this table not included in the paper showing the inclusion of all the variables in the first model of logistic regression. But in the manuscript, we have included the results of the stepwise analysis. If you consider it is appropriate to include this table in the paper, we could add before table 7 or as a supporting additional table.*

| **Birth plan presentation to the hospital** | **Total** | **No**  n (%) | **Yes**  n (%) | ***p*** | ***OR*** |
| --- | --- | --- | --- | --- | --- |
|  | N=416 | n=142 (34.1) | n=274 (65.9) |  | **95%CI** |
| **Intervention Group** |  |  |  |  |  |
| Control | 193 (46.4) | 48 (33.8) | 145 (52.9) | <0.001 | Ref. |
| Intervention | 223(53.6) | 94 (66.2) | 129 (47.1) |  | 0.38(0.24-0.61) |
| **Age** Mean (Sd) | 31.9 (5.5) | 32.3 (5.4) | 31.7 (5.5) | 0.514 | 0.99(0.94-1.03) |
| **Country of origin** |  |  |  |  |  |
| Spain | 304 (73.1) | 99 (69.7) | 205 (74.8) | 0.298 | Ref. |
| Other | 112 (26.9) | 43 (30.3) | 69 (25.2) |  | 0.77(0.47-1.26) |
| **Education** |  |  |  |  |  |
| Primary school or less | 79 (19) | 24 (16.9) | 55 (20.1) | 0.614 | Ref. |
| High school | 177 (42.5) | 65 (45.8) | 112 (40.9) |  | 0.81(0.44-1.5) |
| University | 160 (38.5) | 53 (37.3) | 107 (39) |  | 1.03(0.53-1.99) |
| **Previous birth(s)** |  |  |  |  |  |
| No | 236 (56.7) | 76 (53.5) | 160 (58.4) | 0.351 | Ref. |
| Yes | 180 (43.3) | 66 (46.5) | 114 (41.6) |  | 0.79(0.49-1.29) |
| **Type of birth** |  |  |  |  |  |
| Spontaneous vaginal | 291(70) | 89(62.7) | 202(73.7) | 0.298 | Ref. |
| Operative vaginal | 48(11.5) | 17(12) | 31(11.3) |  | 0.62(0.33-1.18) |
| Caesarean section | 77(18.5) | 36(25.4) | 41(15) |  | 0.75(0.37-1.52) |
| **Epidural** |  |  |  |  |  |
| No | 50 (12) | 18 (12.7) | 32 (11.7) | 0.943 | Ref. |
| Yes | 366 (88) | 124 (87.3) | 242 (88.3) |  | 1.04(0.4-2.68) |
| **Methods of pain relief** |  |  |  |  |  |
| Pharmacological | 224 (53.8) | 87 (61.3) | 137 (50) | 0.032 | Ref |
| Non-pharmacological | 26 (6.3) | 10 (7) | 16 (5.8) |  | 1.08(0.31-3.81) |
| Both | 166 (39.9) | 45 (31.7) | 121 (44.2) |  | 1.9(1.16-3.11) |
| **Early skin-to-skin contact** |  |  |  |  |  |
| No | 43 (10.3) | 21 (14.8) | 22 (8) | 0.131 | Ref. |
| Yes | 373 (89.7) | 121 (85.2) | 252(92) |  | 1.89(0.83-4.3) |
| **Breastfeeding in delivery room** |  |  |  |  |  |
| No | 101 (24.3) | 35 (24.7) | 66 (24.1) | 0.989 | Ref. |
| Yes | 315 (75.7) | 107 (75.3) | 208 (75.9) |  | 1(0.58-1.73) |
| **Maternal complications** |  |  |  |  |  |
| No | 383 (92.1) | 130 (91.5) | 253(92.3) | 0.981 | Ref. |
| Yes | 33 (7.9) | 12(8.5) | 21 (7.7) |  | 0.99(0.44-2.25) |
| **Neonatal complications** |  |  |  |  |  |
| No | 382 (91.8) | 134 (94.4) | 248 (90.5) | 0.138 | Ref. |
| Yes | 34 (8.2) | 8 (5.6) | 26 (9.5) |  | 1.95(0.81-4.7) |

**Comment 36**
Table 5 and line 328-330: how comparable were the two groups? This is a per protocol analysis. Why is this not corrected for confounders, such as parity, etc.? This is really necessary to understand the meaning of these outcomes.
**Response 36**

*Thanks for your indications. We carry out the logistic regression analysis with the confounder’s variables.*

**Discussion**

**Comment 37**
Line 341-342: why is pharmaceutical and non-pharmaceutical pain relief not mentioned here?

**Response 37**

*Thank you for your valuable feedback. We corrected the text.*

*Page 21. Line 390.*

However, it was related to use less frequently epidural analgesia**, and higher combination of pharmacological and non-pharmacological methods for pain relief,**

**Comment 38**
Line 346: add ‘, which was 67% in 2016’ after Department.

**Response 38**

*Thank you again. Done.*

*Page 21. Line 396.*

The percentage of women having received sufficient information about childbirth during pregnancy was higher (around 95%) than the survey carried out by the Catalan Health Department **which was** 67% **in 2016** [18].

**Comment 39**
Line 348: what is meant by shared based counselling? Is it double with based on SDM?

**Response 39**

*We have rephrased the sentence*

*Page 21. Line 397 & 398.*

**This** higher percentage can be explained because all women in **the present study received some BP counselling, standard or based on SDM.**

**Comment 40**
Line 348: replace ‘would be’ with ‘is’.

**Response 40**

*We replaced “would be” with “is”*

*Page 21. Line 398.*

This result **is** in accordance with the Camacho study [22],

**Comment 41**

Line 351-352: sentence is not complete.

**Response 41**

*Thanks. We amended the sentence.*

*Page 21. Line 402-404*

**The women’s intention of using BP in a subsequent pregnancy was high and similar in the two groups and it is consistent with the results of Pennell study [23].**

**Comment 42**
Line 352-356: why is not mentioned that it was one hospital especially where BP were not presented?

**Response 42**

*Thanks for your comment. In fact, we have included the supplementary tables 1 and 2 to show the results by hospitals. Now we have rephrased the sentence.*

*Pag 21. Line 406.*

Despite this, a third of the women did not present BP to the hospital, with a higher number of women who did not present it among those in the IG, **particularly in hospital III where more than half of the women did not present it.**

**Comment 43**

Line 364: elevated compared to what? If you mean high, that give information on what this qualification is based. We know from other research that women score high on satisfaction directly after birth (compared to later in time) and that is influenced by culture.

**Response 43**

*Thanks, you are right, so we reworded the text.*

*Page.22 Line 417 & 418.*

**The grade of** overall satisfaction with the childbirth experience was **high (151.9 of a maximum of 175 points)** and similar in both groups, **and consistent** with the results of the studies by Goodman [28]and Farahat [29].

**Comment 44**
Line 367: add ‘such’ after ‘[30],’.

**Response 44**

*Done*

*Page 22. Line 420.*

There are different dimensions of satisfaction with childbirth and the aspects predicting these dimensions [30]**such** as, the personal control.

**Comment 45**
Line 368-370: sentence doesn't run well, it now suggests that sdm is related with greater satisfaction with sdm process.

**Response 45**

*Thanks for your indication.*

*Page 22. Line 423.*

Some studies suggest that SDM and the use of support elements for their implementation are related to greater satisfaction with the **decision making** [33] in contrast to other studies that do not [34,35]

**Comment 46**
Line 375: replace ‘they’ with ‘the participating women’.

**Response 46**

*Thank you. We have made the change*

*Page 22. Line 429 & 430.*

Possibly, by receiving this counselling, **the participating women** had more knowledge about the alternatives to pain relief and the benefits of early breastfeeding and decided to choose these options

**Comment 47**

Line 384-386: This needs some further explaining. This is a per protocol analysis and the groups might be different. I think it is debatable if these findings are due to the presentation of the BP, other mechanism might be at work like parity. Also early skin-to-skin and breastfeeding might be explained by more vaginal births.

**Response 47**

*Thanks.*

*We modified the text, with the results of the multinomial logistic regression.*

*We also changed reference number 41.*

*Page 23. Line 439-442.*

Likewise, presenting the BP was also associated with a higher probability of using **combined non-pharmacological and pharmacological methods for pain** and early skin-to-skin contact. **These results differ from another study [41], but are consistent with Suarez et al.[16] study in which women with a BP more frequently performed skin-to-skin.**

**Comment 48**

Line 388-391: how is this related to your findings?

**Response 48**

*Thank you for your comment. We have reworded the paragraph and focused on contrasting our results of presentation the BP to the hospital with satisfaction with childbirth with two other similar studies. Reference number 42 has been deleted.*

*Page 23. Line 443-445.*

**The satisfaction childbirth was similar between women who provided the BP and those who did not. This finding is similar to the study by Jolles et al. [20] but differ from Asfhar et al, [21] in which women who used the birth plan expressed significant less satisfaction.**

**Comment 49**
Line 412: where does PtADs stand for?

***Response 49***

*We have removed this acronym.*

**Comment 50**

Line 417: and epidurals

**Response 50**

*We have changed the text*

*Page 24. Line 469- 475.*

An SDM-based counselling intervention during pregnancy was not effective in increasing the presentation of BP in the hospital however it was related to **lower usage of analgesia epidural, a higher combination of pharmacological and non-pharmacological methods for pain relief and initiation of breastfeeding in the delivery room**. Satisfaction related to the birth experience of women was high and similar in both groups. **In addition, the presentation of BP was associated with use pharmacological and non-pharmacological methods for pain relief and early skin-to-skin contact.**
