# Supplementary material for: Effectiveness of birth plan counselling based on shared decision making: A cluster randomized controlled trial (APLANT)
Source: PLoS One. 2022 Sep 12;17(9):e0274240. doi: 10.1371/journal.pone.0274240 (PMC9467369; doi:10.1371/journal.pone.0274240)
Supplement: Attachment — Submitted filename: REBUTT~1.DOC [file pone.0274240.s007.DOC]

Dear editor, and reviewers

We thank you and the reviewers for your further revisions to improve our manuscript entitled “Effectiveness of birth plan counselling based on shared decision making: a cluster randomized controlled trial (APLANT)” PONE-D-21-03373.

We have used the reviewers' comments to improve our article and are pleased to submit the revised manuscript to you.

The reviewers’ comments and our response after each comment are provided below. The responses are highlighted in bold text. The actual changes in the manuscript are provided in bold text and yellow colour.

Please find below the responses to each of the comments and suggestions.

**Reviewer #1: Minor revisions:**
**Comment 1**

Lines 68 and 326: Provide a measure of dispersion for the values 150.2 and 153.4 and identify the type of summary statistics these numbers represent, ie., mean, median, etc.

Response

*Thank for your indication. We provide a measure of dispersion and specified the type of summary statistics*

Line 67- 68: **Mean** satisfaction with childbirth experience was high in the IG and similar to the CG 150.2 **(SD:22.6) vs. 153.4 (SD:21.8)**

Line 345: The mean global satisfaction was high in the control group and similar to the intervention group [153.4 **(SD:21.8)** vs 150.2 **(SD:22.6);** p=0.224)].

**Comment 2**

Lines 254 and 259: Clarify the type of statistical method used for the bivariate analysis and whether the presentation of BP to the hospital was related to the obstetrics outcomes and grade of satisfaction of women.

**Response**

*To clarify the statistics methods and according to your indications and the reviewer's 3, we reviewed and reordered the text. Now Line 260 to 289.*

**Comment 3**

Line 261: Drop “non-parametric test of” from the statement, “For comparison of categorical variables, the non-parametric test of Fisher’s exact test was used.”

***Response***

*Thanks for you indication. We have drop “non-parametric”*

Line 260-261: **For comparison of categorical variables, Fisher’s exact test was used**

**Comment 4**

Line 262: Consider replacing the sentence at line 262 with the following one. To compare quantitative variables, Student’s t-test, ANOVA test, or the Mann-Whitney U test was used.

***Response***

*We have made the modifications according to your recommendations and the advice of reviewer 3.*

Line 260-263**: For comparison of categorical variables, Fisher’s exact test was used; and for the ordinal ones, the Mann-Whitney U test was used. The Student’s t-test was used to compare quantitative continuous variables. A p value <0.05 was considered statistically significant.**

**Comment 5**

Line 268: Clarify if this statement implies that these are univariate analyses. “To determine which obstetric variables (type of birth, epidural, methods of pain relief, breastfeeding in delivery room, early skin-to-skin contact, maternal and neonatal complications) could have a joint relation with presenting the birth plan, we carried out another logistic regression model.

***Response***

*Thanks, this is multinomial logistic regression, so we added the word “multinomial”*

Line 277-280: “to determine which obstetric variables (type of birth, epidural, methods of pain relief, early skin-to-skin contact, breastfeeding in delivery room, maternal and neonatal complications) could have a joint relation with presenting the birth plan, we carried out another **multinomial** logistic regression model”.

**Comment 6**

Line 271: Clarify if the following statement implies that a multivariate analysis was conducted.
“The model was adjusted by variables clinically relevant or who had a level of significance <0.05 in the bivariate analysis (belonging to the control group or intervention group, age, country of origin, education, previous births,).”

***Response***

*Thanks, you are right, so we reworded the previous sentence, adding “multinomial”*

Line 277-283: “To determine which obstetric variables (type of birth, epidural, methods of pain relief, breastfeeding in delivery room, early skin-to-skin contact, maternal and neonatal complications) could have a joint relation with presenting the birth plan, we carried out another **multinomial** logistic regression n model”. The model was adjusted by variables clinically relevant or who had a level of significance <0.05 in the bivariate analysis (belonging to the control group or intervention group, age, country of origin, education, previous births).

**Comment 7**

Line 272: Typically, variables with univariate p-values < 0.20 are included as candidate predictors in multivariate logistic regression models where perhaps stepwise regression is conducted to reduce the model to the most predictive factors.

***Response***

*Thanks for your comments. In fact, we did 2 multinomial logistic regression models. Now we have decided to include the first one in a new table (table 7), as we proposed in the first rebuttal letter (comment 35). Finally, we decided to carry out another multinomial logistic regression model but making an automatic selection of* *variables (stepwise) (Now table 8). We hope this could help to clarify how the analysis was carried out.*

Line:284-289 *:***Additionally, a multinominal logistic regression model was performed modelling the presentation of the birth plan, depending on the variable intervention groups and the variables age, country of origin, education, previous births, type of birth, epidural, methods of pain relief, early skin-to-skin contact, breastfeeding in delivery room, and maternal and neonatal complications, because they were bivariates related to the birth plan presentation, or as adjustment variables for their clinical interest**.

Line 414. **Table 7. Multinominal analysis of the birth plan presentation to the hospital and the intervention groups, demographic and obstetrical variables**

| **Birth plan presentation to the hospital** | ***OR*** | ***P*** |
| --- | --- | --- |
|  | **95%CI** |  |
| **Intervention Group** |  |  |
| Control | Ref. | <.001 |
| Intervention | 0.38 (0.24-0.61) |  |
| **Age** Mean (SD) | 0.9 9(0.94-1.03) | 0.514 |
| **Country of origin** |  |  |
| Spain | Ref. | 0.298 |
| Other | 0.77 (0.47-1.26) |  |
| **Education** |  |  |
| Primary school or less | Ref. | 0.613 |
| High school | 0.81(0.44-1.5) |  |
| University | 1.03(0.53-1.99) |  |
| **Previous birth(s)** |  |  |
| No | Ref. | 0.351 |
| Yes | 0.79(0.49-1.29) |  |
| **Type of birth** |  |  |
| Spontaneous vaginal | Ref. | 0.298 |
| Operative vaginal | 0.62(0.33-1.18) |  |
| Caesarean section | 0.75(0.37-1.52) |  |
| **Epidural** |  |  |
| No | Ref. | 0.943 |
| Yes | 1.04 (0.4-2.68) |  |
| **Methods of pain relief** |  |  |
| Pharmacological | Ref | 0.032 |
| Non-pharmacological | 1.08 (0.31-3.81) |  |
| Both | 1.9 (1.16-3.11) |  |
| **Early skin-to-skin contact** |  |  |
| No | Ref. | 0.131 |
| Yes | 1.89(0.83-4.3) |  |
| **Breastfeeding in delivery room** |  |  |
| No | Ref. | 0.989 |
| Yes | 1(0.58-1.73) |  |
| **Maternal complications** |  |  |
| No | Ref. | 0.981 |
| Yes | 0.99(0.44-2.25) |  |
| **Neonatal complications** |  |  |
| No | Ref. | 0.137 |
| Yes | 1.95 (0.81-4.7) |  |

aOR: adjusted odds ratio; 95% CI: 95% confidence interval

**Comment 8**

Line 297: Consider replacing “basal” with “baseline.”

***Response***

*We replaced the word "basal" with "baseline".*

Line 313 and 322. The **baseline** characteristics of the women are shown in Table 1.

**Comment 9-** Table 1:
A- Define SD. Keep consistent notation throughout the manuscript.
B- Express p-values of 1 as 1.0, in Table 1 and throughout the manuscript.
C- Typographical error for p < 0.0001 for comparing proportions in various education categories.

***Response***

*Thank you for your valuable contributions*

A. *We have added the SD definition at the bottom of the table 1, 2,5 and 6*
B. *We expressed p-values of 1 as 1.0 in the manuscript*
C. *We amended typographical error*

**Comment 11**

Table 4: Perhaps the statistical testing method for comparing age in the two groups can be referred to as a Student’s t-test. ANOVA is typically used when comparing means in more than two groups and it reduces to a Student's t-test when comparing two groups.

***Response***

*Thanks for your indication, we performed a Student’s t-test and we changed the text.*

**Comment 12**

Explain why stepwise logistic regression was used. Perhaps candidate predictors as identified by univariate analyses were selected using stepwise logistic regression. However, this point has not been clearly communicated.

**Response**

*Thanks again for the comment. We explain the analysis process in comment 7. We hope this could help to clarify how the analysis was carried out.*

***Note that line numbering refers to those in the tracked changes version of revision 1.

**Reviewer #2:** I have not comments to your article that is very good and meaningful for midwifery care. The birth plan has proved to be an instrument of great value, especially in countries (such as Brazil) where the care model is still interventionist and therefore does not consider the wishes of the woman.

***Response***

*Thank you for assessment and dedication in reviewing our study.*

**Reviewer #3**: The authors have addressed the comments adequately and the article is now much clearer. There are still some issues that need consideration.

**Comment 1**

Line 58: Add here that midwives did the counseling in the IG.

***Response***

*Thank you for your valuable comment. We modified the text*.

Line 57-60: **Midwives provided BP counselling based on SDM to the women in the intervention group (IG) during their pregnancy, along with a leaflet with evidence-based recommendations.** **Women** in the control group (CG) only received the standard **birth plan** counselling **from midwives.**

**Comment 2**

Line 66-67: This sentence does not read well.

***Response***

*We have rephrased the sentence*

Line 66-67: **Fewer women in the intervention group presented their BP to the hospital compared to those in the control group (57.8% vs 75.1%; p <0.001).**

**Comment 3**

Line 70: ‘Likewise’ is not correct here.

*Thank you. We have removed the word likewise*

Line 70: **Fewer women in the IG used less analgesia epidural (84.7 vs 91.7%; p=0.034), …**

**Comment 4**

Line 86: A ‘d’ is missing after ‘share’.

***Response***

*Thank you again. The error has been corrected*

**Comment 5**

Line 146-147: be clearer what is meant by ‘this’. Suggestion: ‘This later registration …’

***Response***

*Thanks, you are right, we add your suggestion*

Line 145-146: **This later registration** happened because we were not aware at the time whether this was the general practice for this type of intervention studies.

**Comment 6**

Line 190: It should be SDM not SMD.

***Response***

*Done*

**Comment 7**

Line 217: Here the actual obstetric outcomes should be named and why those were chosen. Also, the combined non-pharmacological and pharmacological methods’ should be explained more. What does this involve?

***Response***

*Thanks, we added the methods analysed in the text.*

Line 221-227. Other secondary outcomes, related to the participants, were the obstetric outcomes **(onset of labour, type of birth, episiotomy, early skin to skin contact, initiation of breastfeeding in delivery room, and neonatal and maternal complications), the methods of pain relief [non-pharmacological methods (relaxation and breathing techniques, massage, water use, local heat, birthing ball, others); pharmacological methods (epidural, intravenous analgesia, nitrous oxide, local and general anaesthesia), and combination of both methods]**

**Comment 8**
Line 254-258: The description is not very clear

***Response***

*To clarify the statistics methods and according to your indications and the reviewer's 1, we reviewed, reworded, and reordered the text. Now Line 260 to289.*

**Comment 9**

Line 262: ‘quantitative variables’ is not the right term, also categorical variables are quantitative.

***Response***

*Thanks, done. We have made the modifications according to your recommendations and the advice of reviewer 1.*

Line 260-263: **For comparison of categorical variables, Fisher’s exact test was used; and for the ordinal ones, the Mann-Whitney U test was used. The Student’s t-test was used to compare quantitative continuous variables. A p value <0.05 was considered statistically significant.**

**Comment 10.**

Line 375: There needs to be an ‘s’ at the end of ‘confounder’.

***Response***

*Done. Line 417.*

**Comment 11**

Line 380: I suggest using ‘compared’ instead of ‘with respect’.

***Response***

*Thank you. We have replaced the words*

Line 419- 422: Moreover, the women who presented the BP had double the probability to use non-pharmacological and pharmacological methods for pain relief (aOR=2.06; 95% CI:1.30 to 4.30), and to have early skin-to-skin contact (aOR =2.08; 95% CI: 1.07 to 4.04) **compared** to those who did not present the BP

**Comment 12**

Line 409: It is Elwyn not Ewlyn.

***Response***

*Thank you. The error has been corrected*

Line 450. As stated by **Elwyn**

**Comment 13**

Line 447: Knowledge does not need a capital K.

***Response***

*Thank you. The error has been corrected*

**Comment 14**
Line 470: I suggest starting a new sentence after hospital.

**Response**

*Done*

**Comment 15**

The language issues I mention are just an indication. The article still can use further improvement of the English language. I suggest asking a native speaking (with a science background) to have a look at it.

***Response***

*To ensure the accuracy of the language, grammar and avoid errors related to these aspects, the manuscript has been reviewed by Editage with its corresponding guarantee certificate.*

7. PLOS authors have the option to publish the peer review history of their article ([what does this mean?](about:blank)). If published, this will include your full peer review and any attached files.
[NOTE: If reviewer comments were submitted as an attachment file, they will be attached to this email and accessible via the submission site. Please log into your account, locate the manuscript record, and check for the action link "View Attachments". If this link does not appear, there are no attachment files.]

While revising your submission, please upload your figure files to the Preflight Analysis and Conversion Engine (PACE) digital diagnostic tool, [https://pacev2.apexcovantage.com/](about:blank). PACE helps ensure that figures meet PLOS requirements. To use PACE, you must first register as a user. Registration is free. Then, login and navigate to the UPLOAD tab, where you will find detailed instructions on how to use the tool. If you encounter any issues or have any questions when using PACE, please email PLOS at [figures@plos.org](about:blank). Please note that Supporting Information files do not need this step.
